# Facet Engineering Boosts Interfacial Compatibility of Inorganic‐Polymer Composites

**DOI:** 10.1002/advs.202405175

**Published:** 2024-09-04

**Authors:** Kun Yu, Guangli Ye, Jun Zhang, Liangjie Fu, Xiongbo Dong, Huaming Yang

**Affiliations:** ^1^ Engineering Research Center of Nano‐Geomaterials of Ministry of Education China University of Geosciences Wuhan 430074 China; ^2^ Faculty of Materials Science and Chemistry China University of Geosciences Wuhan 430074 China; ^3^ Laboratory of Advanced Mineral Materials China University of Geosciences Wuhan 430074 China; ^4^ Hunan Key Laboratory of Mineral Materials and Application School of Minerals Processing and Bioengineering Central South University Changsha 410083 China

**Keywords:** Anhydrite, facet engineering, inorganic‐polymer, interfacial compatibility, mechanical properties

## Abstract

The interfacial compatibility between inorganic particles and polymer is crucial for ensuring high performance of composites. Current efforts to improve interfacial compatibility preferentially rely on organic modification of inorganic particles, leading to their complex process, high costs, and short lifespans due to aging and decomposition of organic modifiers. However, the fabrication of inorganic particles free from organic modification that is highly compatible in polymer still remains a great challenge. Herein, a novel facet‐engineered inorganic particle that exhibit high compatibility with widely used polymer interface without organic modification is reported. Theoretical calculations and experimental results show that (020) and (102) facets of inorganic particles modulate local coordination environment of Ca atoms, which in turn regulate d‐orbital electron density of Ca atoms and electron transfer paths at interfaces between polymer and inorganic particles. This difference alters the molecular diffusion, orientation of molecular chains on surface of inorganic particles, further modulating interfacial compatibility of composites. Surprisingly, the facet‐engineered inorganic particles show exceptional mechanical properties, especially for tensile strain at break, which increases by 395%, far superior to state‐of‐the‐art composites counterparts. Thus, the method can offer a more benign approach to the general production of high‐performance and low‐cost polymer‐inorganic composites for diverse potential applications.

## Graphical abstract

The potential of utilizing facet engineering is effectively demonstrated to optimize the interfacial compatibility of IPCs without the use of organic modifiers. The findings revealed the distinctive electron transfer interactions between the (102) facet of AH and the PP molecules. It offers valuable insights into how facet engineering can selectively customize the mechanical properties of composites.

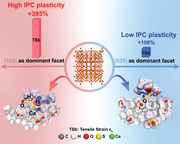

## 1 Introduction

Polymer composites dispersed with inorganic particles (IPCs) possess markedly enhanced mechanical properties compared to pure polymers, which has attracted considerable interest in the field of aerospace, automotive, electrical and electronic, construction, et al.^[^ ^1^ ^]^ It is generally believed that the mechanical properties of the IPCs, especially for plasticity, represent the most critical performance metric, with interfacial compatibility between inorganic and polymer materials being the pivotal determinant of these properties. The interface compatibility between inorganic particles and organic molecules is frequently suboptimal, necessitating organic modification to improve interfacial compatibility.^[^ ^2^ ^]^ However, the interface organic modifiers are prone to aging and decomposition, significantly reducing the compatibility of the composite interface, thus shortening the lifespan of composite materials.^[^ ^3^ ^]^ In addition, the cost of organic modifiers and the modification process accounts for nearly 20% of inorganic particles in IPCs, which limit their sustainable applications.^[^ ^4^ ^]^ In this regard, if inorganic particles without requiring organic modification could be highly compatible with polymers, the aging degradation and high‐cost issues of IPCs might be effectively addressed.

In fact, the significant role of organic modification is to govern the surface polarity and surface energy of IPCs interface via modulating the orientation and diffusion behavior of polymer molecules on the surface of inorganic particles, thus significantly impacting their interface compatibility.^[^ ^5^ ^]^ Previous studies have substantiated that the surface polarity and surface energy of the IPCs interface predominantly stem from the interfacial electron transfer between inorganic particles and polymer molecules.^[^ ^6^ ^]^ Inspired by this understanding, the precise modulation of electron transfer between inorganic particles and polymer molecules may emerge as a promising strategy for enhancing the interface compatibility of IPCs. The electron transfer of IPCs interface is highly related to the inherent structure of inorganic materials.^[^ ^7^ ^]^ Specifically, the variances in the spatial coordination environment of elements in inorganic particles significantly affects the pathways and efficiency of electron transfer between interfaces in composite materials.^[^ ^8^ ^]^ It is theoretically feasible to precisely regulate spatial coordination environment of elements in inorganic particles to optimize the electron transfer at the organic molecule‐inorganic particles interface by modulating the intrinsic structure of the inorganic particles, although it remains a challenging endeavor.^[^ ^9^ ^]^

Facet engineering is a fundamental approach for regulating the interfacial coordination environment of inorganic particles.^[^ ^10^ ^]^ The ratio of surface atoms to total atoms varies with the subtle changes in cell size induced by different degrees of the facet exposure.^[^ ^11^ ^]^ Surface atoms are inherently more unstable than internal atoms due to their relatively low coordination with neighboring atoms. When the crystal is internally doped with trace impurity elements or experiences changes in temperature or pressure during the growth process, radially inward forces are exerted on the surface atoms, resulting in changes in bond lengths between surface atoms and internal atoms. Structurally, changes in bond lengths can induce lattice strain of the crystal on a microscopic scale. This lattice strain also alters bond strength, leading to significant variations in electron density and the surrounding coordination environment of a particular atom, ultimately influencing the macroscopic properties of materials.^[^ ^12^ ^]^ In theory, leveraging facet engineering is viable for adjusting interfacial electron transport in composites through the design of variances in inorganic crystal structures and coordination environments. However, to our knowledge, despite the crucial importance of facets in interfacial electron transport in composites, no attempts have been made to understand the role of inorganic particle facets in modulating the interface compatibility behaviors of IPCs.

Here, as a proof of concept, we report for the first time the facet‐engineered inorganic particles (anhydrite, AH) that exhibit high compatibility with the widely used polymer composite (polypropylene, PP) interface without requiring organic modification. Above facet‐engineered AH shows exceptional mechanical properties in terms of their application in polypropylene (PP) composite, surpassing the state‐of‐the‐art counterparts. By combining theoretical calculations and experiments, we reveal that facet engineering affects electron transfer paths at the interfaces between polymer molecules and AH via regulating the d‐band electron density of AH, thus significantly improving the interfacial compatibility of the IPC. Direct observation of the interfacial behavior between polymer and inorganic particle by advanced analytical methods provides solid proof for the validation of the proposed theory. This work paves an unconventional way for precise regulation of interfacial compatibility between interfaces in composite materials and will enable rational design of inorganic particles to boost the mechanical properties of IPCs.

## 2 Results and Discussion

### 2.1 Design and Optimization of Inorganic Particles by Facet Engineering

**Figure 1:**
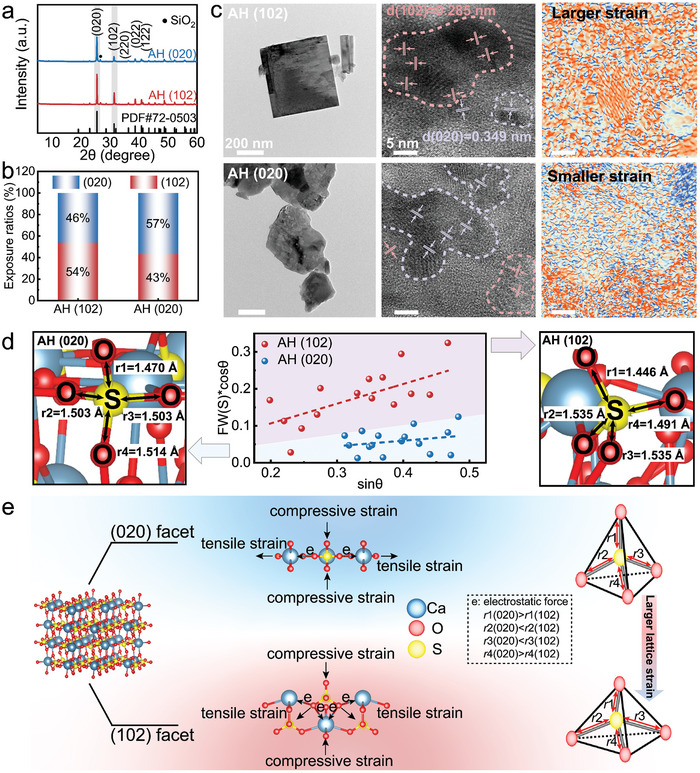
Characterization of AH with different exposure ratios of (020)/(102) facets. a) The XRD patterns of the AH particles. b) The facet exposure ratios of the AH particles. c) The HRTEM images of the AH particles and e_yy_ strain component using geometric phase analysis (GPA) algorithm (The color represents the intensity of strain, with red for highest and blue for lowest). d) The bond lengths of the S─O bond in SO_4_ tetrahedra of (020) and (102) facets by DFT calculations and the Williamson–Hall plot of XRD patterns. e) Schematic diagram of compressive strain and tensile strain in SO_4_ tetrahedra of (020) facet and (102) facet.

The AH particles were synthesized from pretreated phosphogypsum (pre‐PG). Considering the difference in binding energies (E_int_) between the major facets of AH and PP molecules as well as the feasibility of the facet modulation process (Discussion S1; Figures S1–S5, Supporting Information), we chose to modulate the (020) and (102) facets. Two AHs with different exposure ratios were prepared by using different methods (Experimental Section). The diffraction peak intensities of the (020) and (102) facets in AH particles exhibit noticeable differences (**Figure** **1**a). The results of the exposure ratios P of the two synthesized AH particles are shown in Figure 1b, where AH (102) has more (102) facet exposed surfaces, while AH (020) has more (020) facet exposed surfaces. The orientation index M and the relative texture coefficients (RTC) also appear to follow a consistent trend (Discussion S2; Figures S6–S8, Supporting Information). The surface energy and cohesive energy of the (020) and (102) facets were obtained by density functional theory (DFT) (Figure S9, Supporting Information). The (102) facet with higher surface energy is unstable and easily transformed into the (020) facet, which can also be observed from scanning electron microscopy (SEM) (Figure S10, Supporting Information) and high‐resolution transmission electron microscopy (HRTEM) (Figure 1c). The morphology of AH (102) nanoparticle is regular rectangular, while AH (020) nanoparticle shows random grains, where the (020) facet exposed to a high fraction of the AH grows more in the direction of the irregular particles reflecting the increase in entropy. The lattice spacing d_hkl_ of 0.349 and 0.285 nm can be observed, corresponding to the characteristic of (020) and (102) facet in AH particles, respectively. The surface with a higher mesh density has a larger interplanar spacing, resulting in a weaker attractive force (chemical bonds) between adjacent meshes. Hence, the surface with a higher mesh density is inclined to have a lower surface energy (Discussion S3, Supporting Information). The values of surface energies from DFT calculation and d_hkl_ from HRTEM basically comply with this rule. In addition, the corresponding energy dispersive spectroscopy (EDS) mapping confirms the existence and uniform distribution of Ca, S, and O in AH (Figure S11, Supporting Information). The E_int_ of the different facets of AH particles with water molecules were also obtained by using MD calculations (Figure S12, Supporting Information). The E_int_ between water molecules and (102) facet is significantly higher than that between (020) facet and water molecules from Table S4 (Supporting Information). The combination of the contact angle test between AH particles and water molecules (Figure S13, Supporting Information) and the above MD calculations also verified the variation of (020)/(102) facet exposure ratios in the samples.

After optimization of the geometry by the CASTEP module, it was confirmed that the bond lengths *r* in the SO_4_ tetrahedron in the (020) and (102) facets indeed produce a significant difference. This variation in bond length also reflects sufficient lattice strain and is verified in the standard Williamson‐Hall plot (Figure 1d). Distinct lattice strains manifest on the (102) facet compared to the (020) facet. Figure 1e illustrates a schematic of the strain of the SO_4_ tetrahedra in these two facets. The SO_4_ tetrahedra within the (102) facet may experience larger tensile strain owing to the adjacent Ca atoms on both sides, potentially resulting in elevated lattice strain. The Raman spectra (Figure S14, Supporting Information) further demonstrate that a higher exposure ratio of the (102) facet increases the S─O bond stretching force constants, resulting in distortion of the SO_4_ tetrahedra, further verifying the higher lattice strain.^[^ ^13^ ^]^ The variances in lattice strain could modulate the spatial coordination environment of AH, which might affect their inherent electron density.^[^ ^14^ ^]^

### 2.2 Prediction of Facet Engineering in Regulating Interface Compatibility

**Figure 2:**
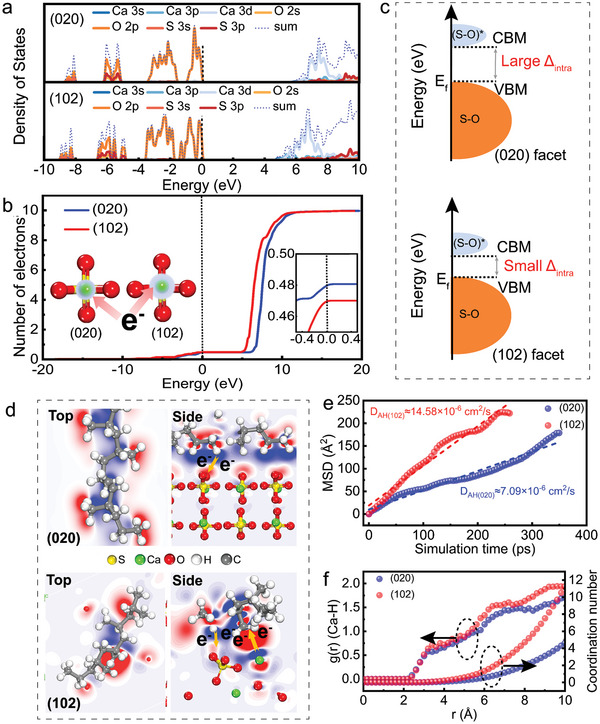
Theoretical calculations predict the mechanism of facet engineering in regulating interface compatibility. a) LDOS of (020) and (102) facets. b) Corresponding number of electrons in the 3d orbital per Ca atom in (020) and (102) facets. c) Schematic illustration of modification of DOS by p orbitals of O and S atoms. d) The EDD between AH and PP polymer of the slices in top and side views (blue and red colors represent charge depletion and accumulation, respectively). e) The MSD plots of the Ca atoms in (020) and (102) facets of the AH/PP composites at 493 K. f) The RDF and C‐N curves of Ca‐H in AH‐PP composites.

To verify the relationship between lattice strain and inherent electron density of AH, the total density of state (TDOS) and partial density of state (PDOS) for (020) and (102) facets were shown in Figures S15 and S16 (Supporting Information). The local density of states (LDOS) of the s, p, and d orbitals of Ca, S, and O were further analyzed as shown in **Figure** **2**a. The degree of orbital overlaps in partial density of state (PDOS) of S_(102)_‐O is larger than that of S_(020)_‐O, which implies that the S 3p orbitals in (102) facet are more likely to hybridize with the O 2p orbitals of the O atoms close to the surface side to form covalent bonds.^[^ ^15^ ^]^ It explains the shorter bond length of the S─O bond close to the surface side, and on the other hand, shows that this hybridization phenomenon is more intense in the (102) facet. The DOS of the Ca 3d orbital for (102) facet shows a negative shift compared with that for (020) facet. To give the accurate number of electrons in the 3d orbitals for each Ca atom in these structures, DOS profiles were integrated the DOS as shown in Figure 2b. The number of electrons in the 3d orbitals of the Ca atoms in the (020) facet is lower than that in the (102) facet. The higher number of electrons suggests that the change in the coordination environment of S─O around Ca atoms can contribute to more electrons being available to Ca atoms on the (102) facet.^[^ ^16^ ^]^ The FTIR spectra of the AH particles also verified the above calculations (Figure S17, Supporting Information). The absorptions at ≈1100, 669, and ≈610 cm^−1^ are attributed to the asymmetric and symmetric S─O stretching vibrations of SO_4_^2−^ and the O‐S─O bending vibrations of SO_4_^2−^. The band at 798 cm^−1^ is attributed to the vibration of Si‐O‐Si.^[^ ^17^ ^]^ The S─O vibrational absorption bands at ≈669 cm^−1^ shifts to a lower wavenumber as the exposure ratio of the (102) facet decreases, and it shifts to a higher wavenumber as the exposure ratio of the (102) facet increases, which is attributed to the electron absorption induced effect of O atoms in CaSO_4_ on different facets. Meanwhile, the DOS of the O 2p and S 3p orbitals for (102) facet shows a negative shift compared with that for (020) facet (Figure S18, Supporting Information), the mechanism is shown in Figure 2c. The conduction band minimum (CBM) level of S─O in the (102) facet produces a downward shift, while the valence band maximum (VBM) of both two facet remains essentially unchanged, resulting in a decrease in the intramolecular energy gap (Δ_intra_) between CBM and VBM. The electronic interactions are facilitated by lowering the CBM level, which reduces the energy barrier required for the transfer of electrons from the VBM to the CBM and is more easily captured by Ca atoms.^[^ ^18^ ^]^ Therefore, Ca atoms on the (102) facet may be more susceptible to electron transfer to the outside,^[^ ^19^ ^]^ and this idea is verified by the fact that the work function of the (102) facet of the AH crystal (7.016 eV) is smaller than that of the (020) facet (7.359 eV) (Figures S19 and S20, Supporting Information).

Usually, the groups of organic modifiers can often physically adsorb or chemically react with the surface of polymers or inorganic fillers, but the importance of alkyl groups of polymer chain on the compatible interfaces between polymer and inorganic filler has been largely ignored at this stage of research. The EDD analyses were carried out using the system consisting of the decylic acid (DA), PP polymer on the (020) facets of AH (Figure S42f, Supporting Information). It can be observed that the alkyl groups on the side of the organic modifier structure near the polymer molecule show a similarly dissolved, covalent‐like state with the polymer (red dashed line), whereas the alkyl groups on the side of the organic modifier structure near the surface of the inorganic material show an electron‐transferred state (blue dashed line), which is also consistent with the phenomenon found by other researchers, which arise and affect parameters of the phases such as the interaction energy between the phases, the dissociation energy, and the stacking density of the ligand.^[^ ^20^, ^21^ ^]^ Therefore, the organic modifier serves as a bridge for charge transfer at the interface between the polymer and inorganic phases. In this study, we achieve this function by modifying the coordination environment of the inorganic phase and regulating the electron transport pathway without the use of organic modifiers. The EDD (Figure 2d) validated the differences in electron transfer pathways in the AH/PP system with different facet exposure ratios. Due to its weak interactions, the Ca atoms in AH (020) show almost no electron transfer with the PP polymer. In contrast, the Ca atoms in AH (102) presented an enhanced electron transfer with the alkyl group (‐CH_3_). The possible electron transfer pathways were depended on the gain and loss of electrons in the AH/PP composite, for example, in the direction of Ca (AH)→H (‐CH_3_)→O (AH). The differences in the electron transfer paths of the molecules on the two facets were further verified by XPS analysis (Figures S21 and S22, Supporting Information). The electrons around the Ca atoms on the (102) facet are more likely to be transferred to the alkyl group, which result in the decrease of the electron density. The weakening of the shielding effect around the Ca atoms and leads to an increase of the binding energy of Ca 2p. The alkyl groups on PP can provide electrons and the electrons around the alkyl group are transferred to S─O,^[^ ^20^ ^]^ which leads to an increase in the electron density around O and an increase in the shielding effect, thus decreasing the binding energy of O 1s in the AH before and after the preparation of composite. The Ca atoms on the (102) facet play the role of charge transfer bridges similar to that of the organic modifiers.

The difference in the electronic structure and coordination environment of Ca in the above two facets further affects the binding properties with PP molecules at the macroscopic scale. The MSDs and diffusion coefficients plotted after MD simulation at 493 K are shown in Figure 2e. It can be found that the diffusion coefficient of Ca atoms in the (102) facet is significantly higher than that of Ca atoms in the (020) facet, which is about twice as high as the latter. This indicates that the movement between the AH (102) and PP molecules is enhanced at the same processing temperature, thus imparting higher fluidity to the PP molecules. In addition, the RDF of Ca or O atoms in the SO_4_ tetrahedra with H atoms on the alkyl group of the PP molecule was used as a characterization parameter to analyze the aggregation position of AH in the composite, as shown in Figure 2f and Figure S23 (Supporting Information). In the cutoff of 10 Å range, the RDF peaks of both facets are greater than 1, which implies that there is a strong interaction between the AH particles and PP molecules,^[^ ^22^ ^]^ and at the same time, the coordination numbers (CNs) of Ca and O atoms in the AH (102)/PP system are significantly higher than that in the AH (020)/PP system. The lower the coordination number, the lower the number of AH combined with PP molecules in the AH/PP system. The MSD, RDF, and CN analyses show that the electron transfer paths between (102)/(020) facets and alkyl groups play a significant role in the orientation and diffusion behavior of PP molecules on inorganic particles, further optimizing the interfacial compatibility of composites.

### 2.3 Direct Observation of Interfacial Features Around Facet‐Engineered Particles

We further investigated the bonding state of polymers and AH between the interfaces by mapping the interfacial regions using atomic force microscopy infrared spectroscopy (AFM‐IR) spectroscopy, in which the AFM tip was used to locally detect the thermal expansion of the samples induced by infrared radiation (Figure S24, Supporting Information). The diffraction peaks at 1378 and 1442 cm^−1^ are the symmetric variable angle absorption of methyl (‐CH_3_) and 1460 cm^−1^ is the symmetric variable angle absorption of methylene (‐CH_2_‐) in PP polymer.^[^ ^23^ ^]^ The AH particles do not produce IR spectral absorption peaks in the above bands.

**Figure 3:**
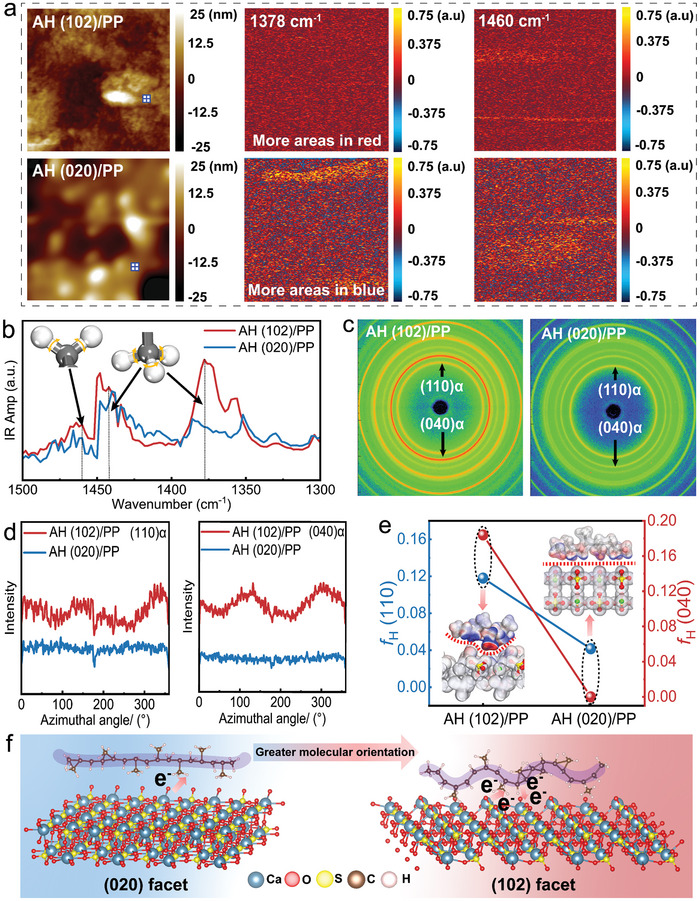
Direct observation of changes in molecular orientation of polymer. a) The topography image (2.5×2.5 µm^2^) and the AFM‐IR chemical maps with irradiation by a laser at 1378 and 1460 cm^−1^ of AH (102)/PP and AH (020)/PP composite. b) Local spectra of the sites marked in blue marked points of a). c) The 2D‐WAXS patterns of the AH/PP composites. d) Azimuthal‐integrated intensity distribution curves of the 2D WAXS patterns. e) The Herman's orientation parameter *f*_H_ of the AH/PP composites with different charge transfer paths. f) Schematic representation of the charge transfer paths at the interface between the two facets and the PP molecule.

The topography images of the composites are shown in **Figure** **3**a. The chemical maps obtained by irradiating the samples simultaneously at 1378 and 1460 cm^−1^ using an infrared laser. Regions with strong AFM‐IR signals are shown in red, while those with reduced intensity are shown in blue. Based on the localized spectra (Figure 3b), the strong localized IR variations of the two composites at 1378, 1442, and 1460 cm^−1^ also verify the local conformational differences of the PP molecules in the two composites. Specifically, the size of the red region at 1460 cm^−1^ in the AH (102)/PP composite increases significantly, which is also consistent with the percentage of colors in the chemical maps calculated using Python (Figure S25, Supporting Information), reflects the fact that the PP molecules are more locally stabilized in the composite containing AH (102) particles. In contrast, the chemical map of the AH (020)/PP composite at 1378 cm^−1^ shows no significant difference relative to the AH (102)/PP composite other than presenting a partially stronger yellow region, which reaffirms the fact that the difference in the two conformations stems from the chemical composition rather than surface artifacts.^[^ ^24^ ^]^

These spectral features of the AH (102)/PP composite are significantly enhanced compared to the corresponding bands of the AH (020)/PP composite, and this change in band intensity is shown to be evidence of interactions between the molecules and the surface of AH. It is shown that the ─CH_3_ and ─CH_2_‐ groups in the PP molecule interact more strongly with AH (102). In addition, these functional groups are likely to be perpendicular to the surface of the AH particles and directly involved in the interaction process. This also perfectly corresponds to the results of the XPS experiments and EDD calculations above. In addition, the stronger relative strength for AH (102)/PP composite may also indicate the higher molecular orientation of the PP molecules due to the buckled geometry.^[^ ^25^ ^]^

The orientation structure of the PP molecules in the composites was further verified using 2D‐Wide‐angle X‐ray scattering (WAXS) (Figure 3c), and the corresponding 1D‐WAXS curves are shown in Figure S26 (Supporting Information). Since the PP composites in this study were obtained in a twin‐screw extruder by heating and mixing, stretching and drawing and cutting, there is a high probability that lamellar crystal structures were produced during the preparation process, while the results of differential scanning calorimetry (DSC) as shown in Figure S27 and Table S5 (Supporting Information) provide the information that the PP composites possessed a significant degree of crystallinity. lamellar crystal structures usually exhibit distinct melting peaks, which is different from the thermal behavior of amorphous or other crystalline forms. Therefore, it can be inferred that the basic crystalline unit of the PP composites in this study is lamellar crystal. The main scattering rings of the composites associated with corresponding the (110)α, (040)α, (130)α, (111)α, and (−131)α facets of PP molecule. The scattering rings of (040)α facet and (110)α facet in AH (102)/PP composite are significantly higher in reflectance brightness and peak intensity than those of AH (020)/PP composites, which suggests that the corresponding lamellar crystals are better oriented in AH (102)/PP composites. In contract, the corresponding lamellar crystals are darker in brightness and weaker in peak intensity in AH (020)/PP composites, and the scattering rings are increasingly fuzzy and blunted, suggesting that the orientation of the samples are getting worse. Figure 3d shows the azimuthal‐integrated intensity distribution curves according to the 2D‐WAXS patterns. The azimuthal‐integrated intensity curves demonstrate varying peak intensities as the azimuthal angle increases.^[^ ^26^ ^]^ Higher peak intensities indicate a greater degree of orientation of lamellar crystals.^[^ ^27^ ^]^ We further calculated the Herman's orientation parameter (*f*_H_) for the(110)α and (040)α facets (Figure 3e). The results show that the *f*_H_ of AH (102)/PP composites are significantly higher than that of AH (020)/PP composites on the (110)α and (040)α planes. This phenomenon demonstrates that the higher surface fraction of the (102) facet remarkably increase the orientation of PP molecules on the surface of AH, which might be caused by the optimal electron transfer path. As a result, as shown in Figure 3f, PP molecules is prone to distribute along the main chain regular arrangement on the (020) facet of AH. In contrast, Ca (AH)→H (─CH_3_)→O (AH) dominated electron transfer path results in the main chain orientation of PP on the surface of (102) facet of AH. The arrangement difference of PP molecules on the surface of AH further plays a crucial role on the mechanical properties of IPCs.

**Figure 4:**
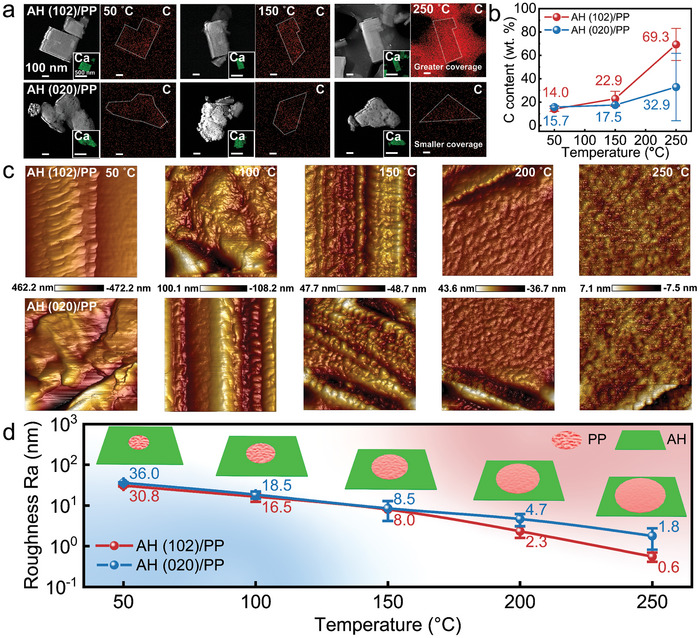
Direct observation of changes in diffusion behavior of polymer. a) The HRTEM images and Ca and C element mapping images of AH (102) particles and AH (020) particles with PP molecules at different temperatures, respectively. b) The relative mass fraction of the C from EDS analysis. c) The AFM topography images (1×1 µm^2^) of PP molecules diffused on the surface of AH (102) particles and AH (020) particles treated at 50, 100, 150, 200, and 250 °C, respectively. d) Surface roughness Ra of the PP molecules diffusion on the surface of AH at different temperatures.

To further observe the diffusion behavior of the PP molecules on the AH particles dominated by the (102) facet and the (020) facet, the HRTEM was utilized to observe the diffusion rate of the PP molecules on the surface of the AH particle under different temperatures. As shown in **Figure** **4**a, the Ca element mapping images show the morphological characteristics and distribution location of the AH particles, and the C element mapping images show that the PP molecules present a uniform distribution on the surface of the AH particles. The distribution of PP molecules on the surface of the two kinds of facet‐dominated AH particles is sparse and the difference between these is less obvious after keeping at 50 °C for 2 min. As the temperature increases, the PP molecules diffuse more on the surface of AH (102) than that of AH (020). When the temperature reaches 250 °C (exceeding the melting temperature of the PP), the mobility of the PP molecules gradually enhanced. The PP molecules covered the surface of the AH (102) particles completely and are more densely distributed. The HRTEM images are also corroborated by the relative mass fraction of C in Figure 4b. Interestingly, the ratio of the relative mass fraction of C on the surfaces of the two AH particles at 250 °C is similar to the ratio of the diffusion rate of the PP molecules on the two facets in the theoretical calculations above. When the PP molecules are just in contact with the surface of the AH, the contact area is relatively small. With the intensification of the diffusion behavior, the PP molecules become more mobile and gradually diffuse in all directions and finally cover the surface of the AH. This diffusion rate of the molecules also tends to be negatively correlated with their surface roughness.^[^ ^28^ ^]^ Therefore, we further observed the diffusion state of the PP molecules using AFM. Figure 4c illustrate a progressive reduction in the roughness of PP molecules at the AH surface with rise temperatures. Following treatment at identical temperatures, the surface roughness of PP after diffusion on AH (102) is notably lower than that observed after diffusion on AH (020), resulting in a smoother surface. Figure 4d shows the difference in surface roughness Ra between the two samples after treatment at 50, 100, 150, 200 and 250 °C. As the temperature increases, the area covered by the PP molecules expands along the planar direction, and the surface wrinkling and Ra decreases consequently. The Ra of AH (102) combined to PP is lower than that of AH (020) combine to PP, which further validates the HRTEM and DSC test (Figures S27 and S28; Table S5, Supporting Information). Combined with the discussion in MSD, RDF, and CN analyses, it is revealed that Ca (AH)→H (‐CH_3_)→O (AH) dominated electron transfer path is beneficial for the diffusion of PP molecules on (102) facet of AH particles, further optimizing the interfacial compatibility between AH particles and PP polymers.

### 2.4 Facet Engineering of (102)‐Dominated AH to Boost Mechanical Properties

**Figure 5:**
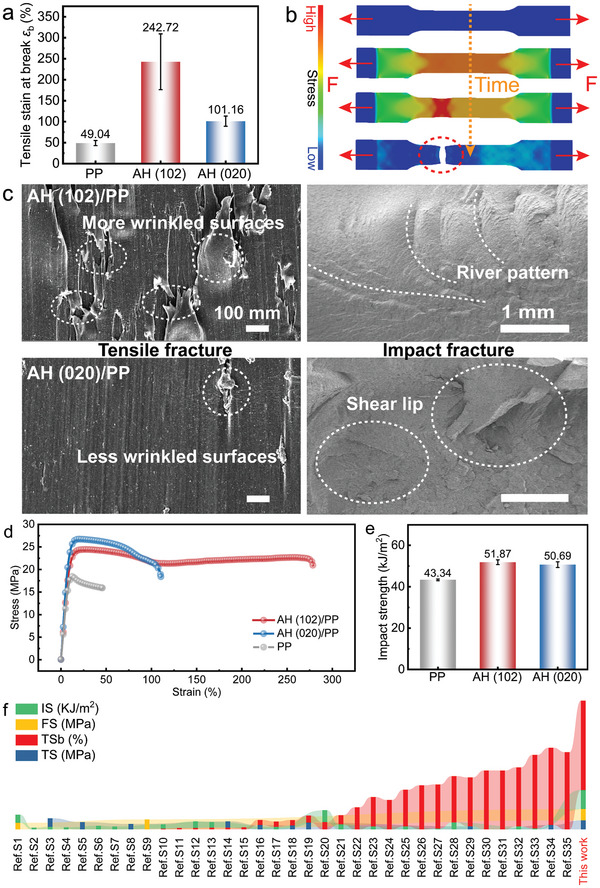
Mechanical property. a) The tensile strains at break ε_b_ of the AH/PP composites. b) The stress distribution of the material during tensile testing. c) The fracture surfaces after tensile failure (left) and impact test (right) of the AH/PP composites. d) The stress‐strain curve during tensile test. e) The impact strength of the AH/PP composites. f) Comparison of the mechanical properties among this work and other composite materials.

The (102) facet of AH favors higher compatibility with PP polymer interface, which is expected to improve the mechanical properties of IPCs. Given the typically high hardness and intrinsic brittleness of inorganic particles, it is widely acknowledged that enhancing the plasticity of inorganic particle‐polymer composites represents one of the most challenging mechanical aspects.^[^ ^29^ ^]^ Surprisingly, the composites with (102) facet‐dominated AH particles dispersed in PP matrices exhibited exceptional their mechanism properties, with the tensile strain at break ε_b_ (TSb) of 242.3% (**Figure** **5**a,d), which increase by ≈395% than that of pure PP. In contrast to (020) facet‐dominated AH particles, the tensile strain at break ε_b_ of (102) facet‐dominated AH particles dispersed in PP matrices is more than 2.4 of magnitude compared to that of (020) facet‐dominated AH. It is worthwhile mentioning that the facet‐engineered AH achieved here surpasses state‐of‐the‐art composites counterparts.

Here, the potential factors, such as composition, hardness, specific surface area, and particle size of AH, that might affect mechanical properties of IPCs are first excluded (Discussion S4, Supporting Information), revealing the significant role of facet engineering in boosting the mechanical properties of inorganic‐polymer composites. Furthermore, we have delved into the micro‐mechanisms of facet engineering to enhance mechanical properties. The specimens for tensile testing were securely clamped at both ends using a fixture throughout the testing process. In the course of composite preparation, microscopic defects or cracks are typically inevitable within the material, both internally and on its surface. These imperfections can detrimentally impact tensile fracture by amplifying or concentrating stresses at these flaws or within the narrower regions of the structure of materials during stretching by the fixtures (Figure 5b). This observation is further corroborated by the occurrence of wrinkled surfaces (Figure 5c) induced by necking in the vicinity of tensile fracture. In contrast to the AH (020)/PP composite, the AH (102)/PP composite exhibit more pronounced folds, indicating a greater resistance to extraction from the PP matrix during the damage process, thereby enabling enhanced energy absorption. As shown in the impact fracture of Figure 5c, the composites with (020) facet‐dominated AH particles show fatigue fracture with transient zones displaying skid marks and shear lips, lacking notable macroscopic plastic deformation. Conversely, the AH (102)/PP composite exhibits a riverine pattern, suggesting a blend of brittle‐ductile fractures, facilitating gradual energy dissipation through the transition from brittle to ductile behavior and enhancing composite plasticity. It is further verified that (102) facet is more favorable to enhance the plasticity of composites. Moreover, additional mechanical properties revealed that the impact of facet engineering on these properties was not notably significant (Figures S29–S32, Supporting Information). Nevertheless, it is noteworthy that tensile strength (TS), flexural strength (FS), and impact strength (IS) of the AH (102)/PP composite exceed those of the relevant national standards (GB/T 20 186.2‐2021, GB/T 38288‐2019, GB/T 35 451.2‐2018, GB/T 36941‐2018, GB/T 24 149.3‐2017, GB/T 24 149.1‐2009 and GB/T 11 016.3‐2009). In addition, the mechanical properties of the polymer composites with (102) facet‐dominated AH particles far exceed those of the AH with organic modification (Discussion S5, Supporting Information). In particular, we first utilize facet engineering to fabricate inorganic‐polymer composite, which is superior to the mechanical properties of the majority of reported organically modified inorganic‐polymer composite counterparts (Figure 5g). As we expected, the 0–96 h UV aging test (at 340 nm wavelength) also demonstrated excellent aging resistance of this composite (Figure S33, Supporting Information), which was prepared using the facet engineering method without the need for organic modifiers. We further applied AH (102) and AH (020) to linear low‐density polyethylene (LLDPE) and found that these facet‐modulated particles could also significantly improve the mechanical properties of LLDPE composites (Figure S34, Supporting Information). This suggests that the present study is generalizable and has broad application prospects.

## 3 Conclusion

In summary, we have effectively demonstrated the potential of utilizing facet engineering to optimize the interfacial compatibility of IPCs without the use of organic modifiers. Our findings revealed that heightened exposure ratios of (102) facets of AH particles markedly bolstered the mechanical properties, especially the plasticity. Surprisingly, the tensile strain at break ε_b_ of the polymer composites with (102) facet‐dominated AH particles surpass that of pure PP by ≈395% and even surpass those of the majority of reported organically modified inorganic‐ polymer composites of a similar kind. These enhancements stem from the distinctive electron transfer interactions between the AH facets and the PP molecules: Variances in electron density of the Ca atoms on the (020) and (102) facets, along with the intramolecular energy gap between the CBM and VBM of S─O, engender diverse coordination environments. Consequently, distinct electron transfer pathways are established with the alkyl groups of the PP molecules, thereby influencing the diffusion and orientation behaviors of the PP molecular chains within the composite. Our findings offer valuable insights into how facet engineering can selectively customize the mechanical properties of composites to meet specific application requirements and for the development of stable, high‐performance composites.

## 4 Experimental Section

### Materials

PG was obtained from Xinyangfeng Agricultural Technology Co., Ltd., China. Its chemical composition is shown in Table S2 (Supporting Information). PP (RA130E) was purchased from Borealis AG Co., Ltd., Austria. Ethyl alcohol (AR) was purchased from Sinopharm Chemical Reagent Co.Ltd., China.

### Pretreatment of Phosphogypsum

Based on the preliminary experimental conditions, a high‐speed crusher was used at 18,000 rpm to crush the PG block for 60 s, resulting in uniform PG powder. Then, we mixed the PG with deionized water at a mass ratio of 1:5 and added 360 g of ZrO_2_ balls to make a slurry. The slurry was then milled using a planetary ball mill at a speed of 400 rpm for 240 min. The refined slurry after ball milling was washed with ethyl alcohol and vacuum‐filtered into filter cake, which was dried for 24 h at 60 °C. Subsequently, the filter cake was ground by three‐head grinder for 15 min to obtain pre‐treated ultrafine PG powder named pre‐PG.

### Preparation of Anhydrite

In this experiment, the pre‐PG powder obtained above was used as raw material to prepare AH with different exposed ratio in two ways:

1) AH (102): Taken 3.75 g of pre‐PG powder and added 75 mL of deionized water to obtain a mixture. The mixture was stirred for 15 min in an ultrasonic environment, then placed into a Teflon vessel (100 mL), sealed in a stainless‐steel autoclave, and kept at 180 °C for 2 h. After the solution temperature dropped to room temperature, the filter cake was obtained by vacuum filtration and washed with room‐temperature deionized water and ethanol. The filter cake was dried at 60 °C for 24 h and ground to obtain AH particles;
2) AH (020): Taken 10 g of pre‐PG powder and placed it in an alumina crucible, appreciate it from room temperature to 600 °C at a heating rate of 5 °C min^−1^, keep it at 600 °C for 2 h, and then naturally cool it down to obtain AH particles.

### Preparation of Polypropylene Composites

According to the previous work foundation (please refer to the supporting documents), PP and filler were blended in a mass ratio of 90:10. To achieve uniform mixing, a twin‐screw extruder was used. The screw diameter of the twin‐screw extruder is 21.7 mm, with an L/D ratio of 40. There are six different heating zones from the starting point to the extrusion endpoint, and the temperature range set is between 215–235 °C. The screw pre‐forming temperature is set at 215 °C, and the screw speed is set at 100 rpm. In the twin‐screw extruder, uniform mixing is achieved by mixing for 10 min and then extruding it at a speed of 10 mm ^−1^s through a steel wire die with a size of 1 mm. The composite particles were soaked in water after cooling and then sent into a granulator through a traction device for production. A vacuum dryer was used to dry the particles at 60 °C for 12 h before storing them in sealed polyethylene bags. An injection molding machine was used to process the particles at 210 °C into standardized specimens.

## Conflict of Interest

The authors declare no conflict of interest.

## Author Contributions

K.Y. performed investigation, methodology, formal analysis, visualization, and wrote‐original draft. G.Y. performed investigation, methodology, and formal analysis. J.Z. performed investigation, methodology, and formal analysis. L.F. performed investigation, methodology, formal analysis, and Supervision. X.D. performed conceptualization, supervision, resources, Wrote‐reviewed and edited. H.Y. performed conceptualization, funding acquisition, supervision, resources, wrote‐reviewed and edited.

## Supporting information

Supporting Information

## Data Availability

The data that support the findings of this study are available from the corresponding author upon reasonable request.
