# Supplementary material for: Facet Engineering Boosts Interfacial Compatibility of Inorganic‐Polymer Composites
Source: Adv Sci (Weinh). 2024 Sep 4;11(41):2405175. doi: 10.1002/advs.202405175 (PMC11538667; doi:10.1002/advs.202405175)
Supplement: Supplementary file 1 — Supporting Information [file ADVS-11-2405175-s001.docx]

**Supporting Information**

**Facet Engineering Boosts Interfacial Compatibility of Inorganic-Polymer Composites**

*Kun Yu^1,2,3^, Guangli Ye^4^, Jun Zhang^4^, Liangjie Fu^1,2,3^, Xiongbo Dong^1,2,3*^, Huaming Yang^1,2,3,4*^*

1 Engineering Research Center of Nano-Geomaterials of Ministry of Education China University of Geosciences, Wuhan 430074, China

2 Faculty of Materials Science and Chemistry, China University of Geosciences, Wuhan 430074, China

3 Laboratory of Advanced Mineral Materials, China University of Geosciences, Wuhan 430074, China

4 Hunan Key Laboratory of Mineral Materials and Application, School of Minerals Processing and Bioengineering, Central South University, Changsha 410083, China

* Corresponding authors

Huaming Yang: hmyang@csu.edu.cn, hm.yang@cug.edu.cn

Xiongbo Dong: dongxiongbo@cug.edu.cn

**This file includes:**

Supplementary Methods

Supplementary Discussions 1 to 5

Supplementary Figures 1 to 43

Supplementary Tables 1 to 8

Supplementary References

# Table of Contents

1. Supplementary Methods 3

2. Supplementary Discussions 10

3. Supplementary Figures 21

4. Supplementary Tables 68

5. Supplementary References 76

1. **Supplementary Methods**

**1.1 Theoretical simulations**

All simulation calculations were run by the FORCITE module and CASTEP module in the Materials Studio (MS) 2020 software. The COMPASS III force field were used in geometry optimization (GO) and molecular dynamics (MD) simulation.^[1]^

**1.2 Molecular dynamics simulation**

The atomic charge was assigned by Forcefield. To calculate non-bonding interactions in periodic systems, the electrostatic energy and Van der Waals forces were estimated using the atom-based technique and the Ewald addition method, respectively. The crystal structures of PG, AH, and the molecular structures of PP were established via Visualizer modulus of MS 2020 software. Before molecular dynamics calculation, the structure usually needs to be optimized by Geometry optimization module. The Smart algorithm was used to optimize the crystal plane model. The calculating accuracy is 4.1868 × 10^-5^kJ/mol.

According to the XRD patterns of PG and AH in **Figure S1**, (020), (-121), (031), (-141) and (002) facets of PG were used as the facets of calculation model. (020), (022), (102), (122) and (220) facets of AH were used as facets of calculation model. The PG unit cell was cut towards (020), (-121), (031), (-141) and (002) facets, and AH unit cell was cut towards (020), (022), (102), (122) and (220) facets.

The E_int_ is a measure of the intermolecular interactions between components and the stability of the system. It can be calculated using **Equation (1)**. A more negative value for E_int_ indicates greater stability in the system, making adsorption or binding more likely to occur. This suggests that additives are more likely to act on crystal surfaces when E_int_ is highly negative. Conversely, when E_int_ is 0 or positive, it becomes difficult for additives to adsorb onto crystals.

$$\begin{aligned} \Delta E=E_{int}=E_{A-B}-{(E}_{A}+E_{B})\#\left( 1. \right) \end{aligned}$$

where $E_{A-B}$ is the total energy of A-B combination, $E_{A}$ and $E_{B}$ refer to the energy of A and B, respectively. These systems utilized the Forcefield COMPASS III and the isothermal isovolumetric (NVT) system. The temperature was maintained at 298 K using the Nose Hoover-Langevin (NHL) method, with a cut-off radius of 0.95 nm and a time step of 0.5 ps. The total simulation time for models is 500 ps. The total simulation steps were 1.0 million, and all models save their output data every 1000 steps.

The surface properties and geometrical parameters of the filler particles, as well as environmental variables such as polymer chain dynamics and free volume, may affect the molecular mobility of the polymer.^[2]^ Several studies have calculated the diffusion coefficients (D) of fillers in polymer using molecular dynamics simulations with mean square displacement (MSD),^[3]^ the D is calculated by using the following **Equation (2)**:^[4]^

$$\begin{aligned} D=\frac{1}{2}\lim_{\Delta t\to\infty} \frac{dMSD}{d\Delta t}=\frac{1}{6N}\lim_{\Delta t\to\infty} \frac{d}{dt}\sum_{i=1}^{N} \left( \left[ r_{i}\left( t \right)-r_{i}\left( 0 \right) \right]^{2} \right)\#\left( 2 \right) \end{aligned}$$

where $N$ is the total number of model, $t$ is the time, $r_{i}\left( t \right)$ is the Cartesian position vector of atom $i$ at time $t$ and $r_{i}\left( 0 \right)$ is the initial position.

The radial distribution function (RDF) is a useful method to describe the aggregation state of molecules. In a statistical mechanical multiparticle system, the g(r) function represents the probability of finding other particles within a distance r around a central particle. It reflects the mode and nature of interactions between nonbonded atoms.^[5]^ The g(r) and coordination number (CN) can be obtained through the **Equation (3)** and **Equation (4)**:^[6]^

$$\begin{aligned} g\left( r \right)=\frac{Vn\left( r,\Delta r \right)}{N4\pi r^{2}\Delta r}\#\left( 3 \right) \end{aligned}$$

$$\begin{aligned} CN=4\pi\rho\int_{0}^{r} g\left( r \right)r^{2}dr\#\left( 4 \right) \end{aligned}$$

where $V$ is the total volume of the simulated system; N is the total number of the simulated system; $n\left( r,\Delta r \right)$ is the number of particles from $r$ to $r+\Delta r$, $r$ is the bulk density (ratio of the number of particles to the bulk volume) and $\rho$ is the number density of the coordination atom (or molecular).

Therefore, the MSD and RDF analytical methods can be used to assess the effect of fillers on the mobility of polymer molecules. For this part of the molecular dynamics calculations: we utilized the Forcefield COMPASS III, the isobaric isothermal (NPT) system and NVT system. The Nose method was used to maintain the temperature at 493 K (close to the extrusion temperature of the composites), with a cutoff radius of 0.95 nm and a time step of 0.5 ps. The total simulation time for the model was 500 ps, the total simulation steps were 1 million, and the output data were saved every 1000 steps for all models

**1.3 DFT calculation**

The density function theory (DFT) was performed using generalized gradient approximation (GGA) and Perdrew–Burke–Ernzerhof (PBE) exchange correlation functional in CASTEP module. The cutoff energy with the value of 571.40 eV based on the OTFG ultrasoft pseudopotentials was used in all of the calculations. The the geometry optimization was set to be 0.01 eV Å^-1^ on force. The total energy difference was less than 5 × 10^-5^ eV atom^-1^. For the surface and interface calculations, the self-consistent-field (SCF) convergence accuracy was set to 1.0 × 10^-6^ eV atom^-1^. The (020) surface and (102) surface and a vacuum thickness of 20 Å was applied.

The surface energy is the reversible work needed to create a unit surface area. A lower value indicates greater stability and accuracy of the surface structure. To simplify our experiment, we utilized the CASTEP tool to calculate the energy of two main exposed surfaces of anhydrite: (020) and (102). The atomic layer thickness ranges from 6.669 Å to 11.394 Å, while the vacuum layer thickness ranges from 15 Å to 20 Å. The size of (020) surface slab is 12.476 Å × 13.982 Å × 20.895 Å (a × b × c), while the size of (102) surface slab is 16.833 Å × 13.992 Å × 26.461 Å (a × b × c). The surface energy (E_surf_) of a slab model was calculated by **Equation (5)**:

$$\begin{aligned} E_{surf}=\frac{E_{slab}-\left( \frac{N_{slab}}{N_{bulk}} \right)E_{bulk}}{2A}\#\left( 5 \right) \end{aligned}$$

where $E_{slab}$ and $E_{bulk}$ are the total energies of the surface slab and bulk unit cell, respectively; $N_{slab}$ and $N_{bulk}$ are the numbers of atoms in the slab and bulk unit cells, respectively; A is the unit area of the surface; 2 denotes two surfaces along the z-axis in the surface slab. Low values indicate surfaces that are thermodynamically stable and have low reactivity. On the other hand, high values suggest fewer stable surfaces that tend to lower their surface energy by reacting with their surroundings or undergoing surface structure re-ordering.

The cohesion energy is the energy released by free atoms to form a compound; the higher the cohesion energy, the more stable the compound. It is well known that the cohesive energy is calculated by the ab-initio method. The cohesive energy per atom was calculated by **Equation (6)**:

$$\begin{aligned} E_{coh}=\frac{\sum E_{atom}-E_{total}}{N}\#\left( 6 \right) \end{aligned}$$

where $E_{coh}$ is the cohesive energy, $E_{atom}$ is the total energy of a neutral atom, $E_{total}$ is the total energy of the material, and $N$ is the number of atoms in the material.

**1.4 Characterizations method**

The phase composition was determined by X-ray powder diffraction spectrometry (XRD, Bruker D8 advance, Germany) with Cu Kα radiation (λ =1.5406 Å, V = 40.0 kV, and I = 40.0 mA) at a scanning rate of 2 °/min for a 2θ range of 5−90°.

The functional groups of IPCs were characterized by using Fourier transform infrared spectrometer (FTIR, Nicolet iS 50, Thermo Fisher Scientific Inc., USA).

The chemical structure and molecular interactions of the IPCs were investigated using Raman spectroscopy (WITec Confocal Raman microscope alpha300 R, Germany).

The particle sizes and specific surface area of the samples were measured using a laser particle sizer (Bettersize 3000, Bettersize Instruments ltd., China).

The compositions of the samples were investigated using a wavelength-dispersive X-ray fluorescence spectrometer (XRF, Thermo Scientific ARL PERFORM’X, USA).

The PG and AH particles were compressed into cylindrical sheets with dimensions of 13 mm × 2 mm. The hydrophilicity of the PG and AH were measured using contact angle analyzer (DSA100, Kruss, Germany).

The morphologies and structures of the samples were investigated by scanning electron microscopy (SEM, ZEISS Gemini SEM 300, Germany), and energy-dispersive spectrometry (EDS) was carried out at the same time.

The samples were further observed using transmission electron microscopy (TEM, Tecnai G2-F20) and high-resolution TEM (HRTEM), and selected area electron diffraction (SAED) was performed in conjunction with the TEM measurements.

The elemental states were analyzed using X-ray photoelectron spectrometry (XPS, Thermo Scientific KAlpha, USA).

The thermal decomposition and thermal stability of PG, AH, and AH/PP composites were analyzed by TG and DSC evaluations (STA 449F5, and DSC 214, Netzsch, Germany). All samples were tested under nitrogen. The thermal decomposition of PG and AH was evaluated by heating the samples from room temperature to 1200 °C at a heating rate of 10 °C/min. The thermal stability of the composites was evaluated in three test phases. (1) The first heating stage: the temperature was ramped up from 20 °C to 200 °C at a rate of 10 °C/min, and was held at 200 °C for 10 min. (2) The first cooling stage: the temperature from 200 °C to 20 °C at a cooling rate of 10 °C/min. (3) The second heating stage: the temperature was ramped up from 20 °C to 200 °C at a rate of 10 °C/min.

AM-IR was performed using an Anasys nanoIR3, Bruker, USA Resolution: XY0.2nm Z<0.1nm Spatial resolution of the infrared spectral image 10nm Surface roughness was tested by Burker Dimension FastScan/Icon.

Two-dimensional wide-angle X-ray Scattering (2D-WAXS, Xeuss 2.0, Rigaku Xenocs, France) was carried out with an X-ray wavelength of 0.154 nm. The scattering pattern was collected by a detector (Pilatus 3R 300K) with a resolution of 2048 × 2048 pixels and a pixel size of 172 × 172 μm^2^. The sample-to-detector distance and acquisition time of WAXS measurement were 88 mm and 300 s, respectively. The 2D scattering images were analyzed with Fit2D software from the European Synchronization Radiation Facility. The Herman's orientation parameter (*f*_H_) from the azimuthal-integrated intensity distribution curves of the X-ray scattering patterns were calculated by using the following equations:^[7]^

$$\begin{aligned} f_{H}=\frac{3\left\langle{cos}^{2}\varphi\right\rangle-1}{2}\#\left( 7 \right) \end{aligned}$$

$$\begin{aligned} \left\langle{cos}^{2}\varphi\right\rangle=\frac{\int_{0}^{\pi/2} I(\varphi){cos}^{2}\varphi sin\varphi d\varphi}{\int_{0}^{\pi/2} I(\varphi)sin\varphi d\varphi}\varphi\#\left( 8 \right) \end{aligned}$$

where $\varphi$ is the angle between the normal direction of the specific crystal lattice and the reference axis, $I(\varphi)$ is the intensity distribution along with $\varphi$. When *f*_H_ equals to 1, it represents that the molecular chains are ideally parallel to the stretching direction, while *f*_H_ = 0 indicates isotropic.

The tensile and flexural performance were tested on a 10 KN microcomputer-controlled electronic universal testing machine (MTS Industrial Systems CMT4104, China.) according to GB/T 1040.2-2006 and GB/T 9341-2008. The impact strengths of the composites were measured using an Izod impact tester (YF-8012, Yangzhou Yuanfeng Testing Machine Factory, China) according to the GB/T 1843–2008 standard.

Considering the variability of the samples, at least seven samples were tested for each group. The heat deflection temperature (HDT) was tested using a thermal deformation vicat testing machine (Shenzhen SUNS Technology VTM1200, China). The rectangular specimen size was 80 mm×10 mm×4 mm according to GB/T 1634-2019 standard, and the load size was selected as 0.45 MPa according to the B method in GB/T 1634.2-2004. The starting temperature of the heating device should be in 20 °C-23 °C. The heating speed is 2 °C/min.

The shore hardness of the PG/PP and AH/PP composites was measured using a shore durometer (Naibo Testing Technology. Co., Ltd, China).

1. **Supplementary Discussions**

**2.1 Supplementary Discussion 1**

**The MD calculation between the facets of AH and PP**

Till now, the mechanical properties of IPCs have mainly been improved by regulating filler morphology, aspect ratio, particle size,^[8]^ but there are few reports discussing the effect of different exposed facets of fillers on interfacial compatibility. From this point of view, if we can predict the performance of the material with different exposed facets through theoretical calculations in advance, material design could be efficient and target-oriented. The phase with obvious differences in the crystal structure can be synthesized by simple calcination or chemical method of PG,^[9]^ so based on theoretical calculation, it has been realizable to get the specific structure with the expected properties. To predict the crystal structure with satisfying mechanical properties inverse way, we used MD to calculate E_int_ between the five major facets of PG and PP, as well as between the main five facets of AH and PP (**(Figure S2**). The model maintains good temperature and energy stability over simulation time (**Figure S3** and **Figure S4**). As shown in **Table S3**, the trends of the E_int_ between different facets of PG and PP are as follows: (020)<(-141)<(031)<(-121)<(002), the trends of the interaction energy between different facets of AH and PP are as follows: (122)<(020)<(102)<(220)<(022). By comparing the mean and median values of the E_int_ between the facets and PP, the E_int_ between the major facet of AH particles and PP molecule is significantly lower than that of PG, which may lead to a stronger attraction between AH and PP and thus improve the mechanical properties of the IPC. The mechanical experimental data (**Figure S5**) is also in good agreement with the conclusions of MD simulation. The tensile strength, flexural strength, impact strength, flexural modulus, and tensile strain at break ε_b_ were significantly improved, especially the ε_b_ was improved by about 419.2%. We use the AH to execute following facet engineering since the terminal goal of this research is to obtain the IPC with high mechanical performance. **Table S3** shows that the (122) facet has the lowest E_int_ with PP molecules (E_intAH(122)_PP_=-69.6173 kcal/mol), indicating that the (122) facet is easier to tightly interact with PP molecules, leading to the better mechanical properties. However, it is hard to control the exposure of this facet in our practical synthesis routes. Relatively, (020) and (102) facets have higher content in crystal and their exposure could be easier to implement. The E_int_ of the (020) facet with PP molecules is -67.9419 kcal/mol which is close to E_intAH(122)_PP_.

**2.2 Supplementary Discussion 2**

**The calculations of orientation index M, the relative texture coefficients (RTC) and the exposure ratios P of the (020) and (102) facets of AH particles**

To quantitatively assess the orientation of these facets, the orientation index *M* was calculated as follow:^[10]^

$$\begin{aligned} M_{\left( hkl \right)}=\frac{{I\left( hkl \right)}/{\sum I\left( h^{'}k^{'}l^{'} \right)}}{{I_{0}\left( hkl \right)}/{\sum I_{0}\left( h^{'}k^{'}l^{'} \right)}}\#\left( 9 \right) \end{aligned}$$

where $I\left( hkl \right)$ is the X-ray diffraction intensity from the experimental data, $I_{0}\left( hkl \right)$ is X-ray diffraction intensity from JCPDS cards. $\sum I\left( h^{'}k^{'}l^{'} \right)$ in the present case is the sum of the intensities of five independent peaks: (020), (102), (220), (022) and (122). **Figure S6** displays the $M_{\left( hkl \right)}$ of the AH particles. The $M$ values of (020) and (102) facets exhibit significant variations across all AH particles, whereas the fluctuations in the $M$ values for (220), (022), and (122) facets are comparatively minor. To further demonstrate the variations in the orientation and exposure of facets, the relative texture coefficients ($RTC$) of each facet were calculated using the following formula:^[11]^

$$\begin{aligned} {RTC}_{\left( hkl \right)}=\frac{{I\left( hkl \right)}/{I_{0}\left( hkl \right)}}{\sum\left( {I\left( hkl \right)}/{I_{0}\left( hkl \right)} \right)}\times100\#\left( 10 \right) \end{aligned}$$

**Figure S7** displays the $RTC$ of the AH crystals on (020) and (102) facets. For the (020) facet, the order is AH (020) (30.34)>AH (102) (20.77), while for the (102) facet, the order is AH (102) (27.33)> AH (020) (20.50). These findings are consistent with the calculation data for these facet orientations, which confirms the successful synthesis of AH particles with varying orientation. To determine the percentage of exposed facets, the current international standard surface area ratio method was used.^[12]^

However, there is limited literature available on calculating the exposure ratios of (020) and (102) facets for the AH particles. Inspired by the research work on calculating the fact exposure ratios such as TiO_2_,^[13]^ we have derived the formula for calculating the (020) facet and (102) facet exposure ratios for the AH particles. The key to the calculation of the exposure ratio is to calculate the surface area of (020) and (102) faces. As we know, the AH is usually an orthogonal crystal system, and the AH particles synthesized in this experiment are also a common orthogonal crystal system. Therefore, the area of (020) facet ($S_{020}$) is *b*×*c*, and the area of (102) facet ($S_{102}$) is *b*×*l*. How to calculate *l*? When the crystal structure was rotated until the y-axis is perpendicular to the paper surface (**Figure S8**). The angle θ between the (102) facet and the (002) facet is calculated by the following equation:

$$\begin{aligned} cos\theta=\frac{\frac{h_{1}h_{2}}{a^{2}}+\frac{k_{1}k_{2}}{b^{2}}+\frac{l_{1}l_{2}}{c^{2}}}{\sqrt{\left( \frac{h_{1}^{2}}{a^{2}}+\frac{k_{1}^{2}}{b^{2}}+\frac{l_{1}^{2}}{c^{2}} \right)\left( \frac{h_{1}^{2}}{a^{2}}+\frac{k_{1}^{2}}{b^{2}}+\frac{l_{1}^{2}}{c^{2}} \right)}}\#\left( 11 \right) \end{aligned}$$

where a, b and c are the theoretical cell parameters of CaSO_4_ (JCPDS card no.72-0503). By calculation, θ is obtained as about 24.04°, so $S_{102}$= *b*×*l*=*ab*/cosθ.

The exposure ratios P of the (020) and (102) facets were calculated using the following formula:

$$\begin{aligned} P_{S_{020}}=\frac{S_{020}}{S_{020}+S_{102}}\times100\%=\frac{c}{c+\frac{a}{cos\theta}}\times100\%\#\left( 12 \right) \end{aligned}$$

$$\begin{aligned} P_{S_{102}}=\frac{S_{102}}{S_{020}+S_{102}}\times100\%=\frac{\frac{a}{cos\theta}}{c+\frac{a}{cos\theta}}\times100\%\#\left( 13 \right) \end{aligned}$$

where the value of θ is 24.04°, the $S_{020}$ and $S_{102}$ correspond the areas of the (020) and (102) facets of the AH particles, respectively.

**2.3 Supplementary Discussion 3**

**The surface energy and cohesive energy of two facets**

The difference in surface energy $E_{surf}$ comes mainly from the difference between the atoms constituting the surface and their coordination environment. If lattice strain and bond length changes occur during crystal growth, the $E_{surf}$ would change, so from the energy point of view, their surface charge environments would also be different if two substances with differences in $E_{surf}$ can be regulated. Surface energy and cohesive energy were calculated for the (020) and (102) facets of AH particles, as shown in **Figure S9**. The surface energy of the (020) facet is significantly lower than that of the (102) facet. It is obvious that the (020) facet is more stable and is the most commonly exposed facet compared to the (102) facet. It also shows that the (020) facet has a very thermodynamic stable. The cohesive energy shows an opposite pattern to the surface energy, with the cohesive energy of the (020) facet being significantly higher than that of the (102) facet. The cohesive energy is usually inversely related to the thermal expansion coefficient.^[14]^ The thermal expansion coefficient of PP is about 3-4×10^-4^ K^-1^ in the 300-450 K temperature range,^[15]^ while the thermal expansion coefficient of AH in this study is much smaller than that of PP.^[16]^ The thermal expansion coefficient of polymer composites is usually determined by each component, and when the difference in the thermal expansion coefficient between the materials is large, the shear effect due to the temperature change is stronger, and thus the degree of crystallinity increases. The thermal expansion coefficient of the (020) facet is more different from that of PP, and the crystallinity obtained by the subsequent DSC test also verifies the above conclusion.

**2.4 Supplementary Discussion 4**

**Effect of the composition, hardness, specific surface areas, particle size on mechanical properties of materials**

From the XRF analysis (**Table S2**), it is known that the main composition of PG and AH particles is CaSO_4_, followed by SiO_2_, and contains trace amounts of metal oxides, therefore the interference of impurities in PG/AH on the mechanical properties can be excluded. In general, hardness reflects to some extent the mechanical properties of a material. However, for most materials, no theory or equation establishes an exact relationship between hardness and other more purely physical quantities, such as yield strength or tensile strength. **Figure S35** shows that AH/PP composites exhibit extremely similar shore hardness, thus the effect of hardness on the above mechanical properties also can be excluded. The mechanical properties are often also related to the contact area between phases, and the AH particles have very close specific surface areas (standard deviation of about 0.314, **Figure S36**), so this effect can also be excluded.

The maximum particle size difference Δ between the AH (102), AH (020), were found by particle size testing to be 0.024 μm, 0.209 μm, and 1.183 μm for D10, D50, and D90, respectively (**Figure S37** and **Table S6**). To verify whether it is the difference in particle size at this level that leads to the large differences in the mechanical properties of the composites, we prepared various AH by controlling the ball milling time, and tested the mechanical properties of the composites. The different particle sizes of AH by controlling the ball milling time are shown in **Table S7** and the mechanical properties of the composites are shown in **Figure S38**.

Experimental method: Based on our preliminary experimental conditions, we used a high-speed crusher at 18,000 rpm to crush the PG block for 60 s, resulting in uniform PG powder. Then, we mixed the PG with deionized water at a mass ratio of 1:5 and added 360 g of ZrO_2_ balls to make a slurry. The slurry was then ground using a planetary ball mill at 400 rpm for 60, 90., 120, 240 and 480 min respectively. The refined slurry after ball milling was washed with ethyl alcohol and vacuum-filtered into filter cake, which was dried for 24 h at 60 °C. Subsequently, the filter cake was ground by three-head grinder for 15 min to obtain pre-treated ultrafine PG powder named pre-PG. The pre-PG with different particle sizes was then synthesized into AH with different particle sizes according to the synthesis method of AH (020), and then composited with PP to make sample to test the mechanical properties.

It can be observed that the mechanical properties of the samples improved when the ball milling time was increased from 120 min to 480 min, at which time the differences in particle size between the two were: 0.0645 μm, 0.6750 μm, and 3.1310 μm for D10, D50, and D90, respectively (**Table S7**). The difference in particle size at this level was significantly higher than that of the samples of AH (102) and AH (020), but the enhancement of the mechanical properties of the composites is much lower than that of AH (102)/PP and AH (020)/PP composites, especially tensile strain at break ε_b_.

Therefore, the differences in particle sizes of AH (102) and AH (020) affect the mechanical properties of the composites, however, the degree of this effect is relatively low and is not the primary reason for the significant differences in mechanical properties shown in **Figure 5**. To further support this conclusion, we conducted simulation analysis.

The finite element method (FEM) provides a new approach to the study of the tensile process of composite. The ANSYS Workbench has obvious advantages in the establishment FEM and result processing, and the LS-DYNA program can quickly solve dynamic problems such as large deformation, fracture, etc. generated in the tensile process, as well as a variety of complex nonlinear contact problems.^[17]^ The integration of the two makes the mechanical analysis, which was originally extremely complex and tedious, much less difficult while reducing the experimental cost and time.^[18]^ The purpose of this part is to verify the effect of AH with particle sizes of 1 μm, 2 μm,3 μm, and 4 μm on the tensile properties of the composites through finite element numerical simulations with ANSYS/LS-DYNA to exclude the differences in the mechanical properties in the main text are caused by the small differences in particle size.

The first step is to use the Design Modeler software to design a test model that was the true size of the actual tensile test specimen.

The second step is to select the material parameters of PP and AH and build the AH/PP composite using Material Designer software. The experimental model with the mesh and a representative volume element (RVE) (random particle) model (**Figure S40a**) were developed using PP as the matrix and AH with different particle sizes as the particles, the parameters shown in **Table S8**.

The third step is based on the explicit dynamics analysis-LS-DYNA of the model including meshing, mesh quality control, the definition of materials used, load settings, and actual experimental conditions. From meshing analysis, the results for the number of nodes are 1680 and 1560 elements respectively. In LS-DYNA analysis, boundary requirements are used. These parameters are the initial requirements for the simulation. Total simulation period = 0.3 s, the left side of the model is fixed, and the right side is set to be displaced by 20 mm in the x-direction while constraining the model in the z-direction (**Figure S38**). The following criteria analyzed for tensile properties of composites: equivalent stress and strain.

**Figure S40b** and **S40c** shows the last frames of equivalent stress and strain of the sample in the tensile stage respectively, the fracture region is consistent with the actual fracture region. In addition, **Figure S40d** shows the graphs of stress and strain as a function of simulation time. It can be observed that samples with particle size intervals of 1 μm apart do affect the mechanical properties of the composites. In particular, when the particles are increased from 1 μm to 2 μm, the tensile strength and tensile strain at fracture of the composites are most obviously enhanced from 17.693 MPa and 60.888% to 19.196 MPa and 85.681%, respectively, but this enhancement is much weaker than that shown in **Figure 5**. From the above experimental data on particle size and this part of the simulation analysis, we can determine that the small differences in particle size between AH (102) and AH (020) are not the main reason for the great differences in the mechanical properties of their composites. Hence, it can be inferred that the changes in mechanical properties is attributed to the facets of AH.

**2.5 Supplementary Discussion 5**

**Mechanical properties of samples of AH modified with organic modifiers and PP composites and EDD analysis between materials after addition of modifier**

Currently, commonly used organic modifiers can be classified into coupling agents, fatty acids, and phosphates.^[19]^ For example, after the modification of AH with palmitic acid (PA), the flexural strength of AH@PA/PP is about 13.8% higher than that of pure PP, the tensile strain at break ε_b_ is about 286.2% higher than that of pure PP, the tensile strength, impact strength, and flexural modulus have also been improved to various levels (**Figure S43a-e**). However, it still did not reach the mechanical properties of AH/PP composites modified without modifier in this study. In addition, we modified AH with coupling agent KH550, coupling agent KH590, trimethoxy(octadecyl)silane (OTS), dimethyldimethoxysilane (DMDMS), tetra-n-butyl titanate (TBT), and stearic acid (SA), and none of the composites reached the tensile strains at break ε_b_ of the unmodified AH/PP composites in this study. (**Figure S44**).

Usually, the groups of organic modifiers can often physically adsorb or chemically react with the surface of polymers or inorganic fillers, but the importance of alkyl groups of polymer chain on the compatible interfaces between polymer and inorganic filler has been largely ignored at this stage of research. Since the difference between PA and decylic acid (DA) is mainly the carbon chain length, to save calculation time, the EDD analyses were carried out using the system consisting of the DA, PP polymer on the (020) facets of AH (**Figure S43f**). It can be observed that the alkyl groups on the side of the organic modifier structure near the polymer molecule show a similarly dissolved, covalent-like state with the polymer (red dashed line), whereas the alkyl groups on the side of the organic modifier structure near the surface of the inorganic material show an electron-transferred state (blue dashed line), which is also consistent with the phenomenon found by other researchers, which arise and affect parameters of the phases such as the interaction energy between the phases, the dissociation energy, and the stacking density of the ligand.^[20]^ Therefore, the organic modifier serves as a bridge for charge transfer at the interface between the polymer and inorganic phases. In this study, we achieve this function by modifying the coordination environment of the inorganic phase and regulating the electron transport pathway without the use of organic modifiers.

1. **Supplementary Figures**


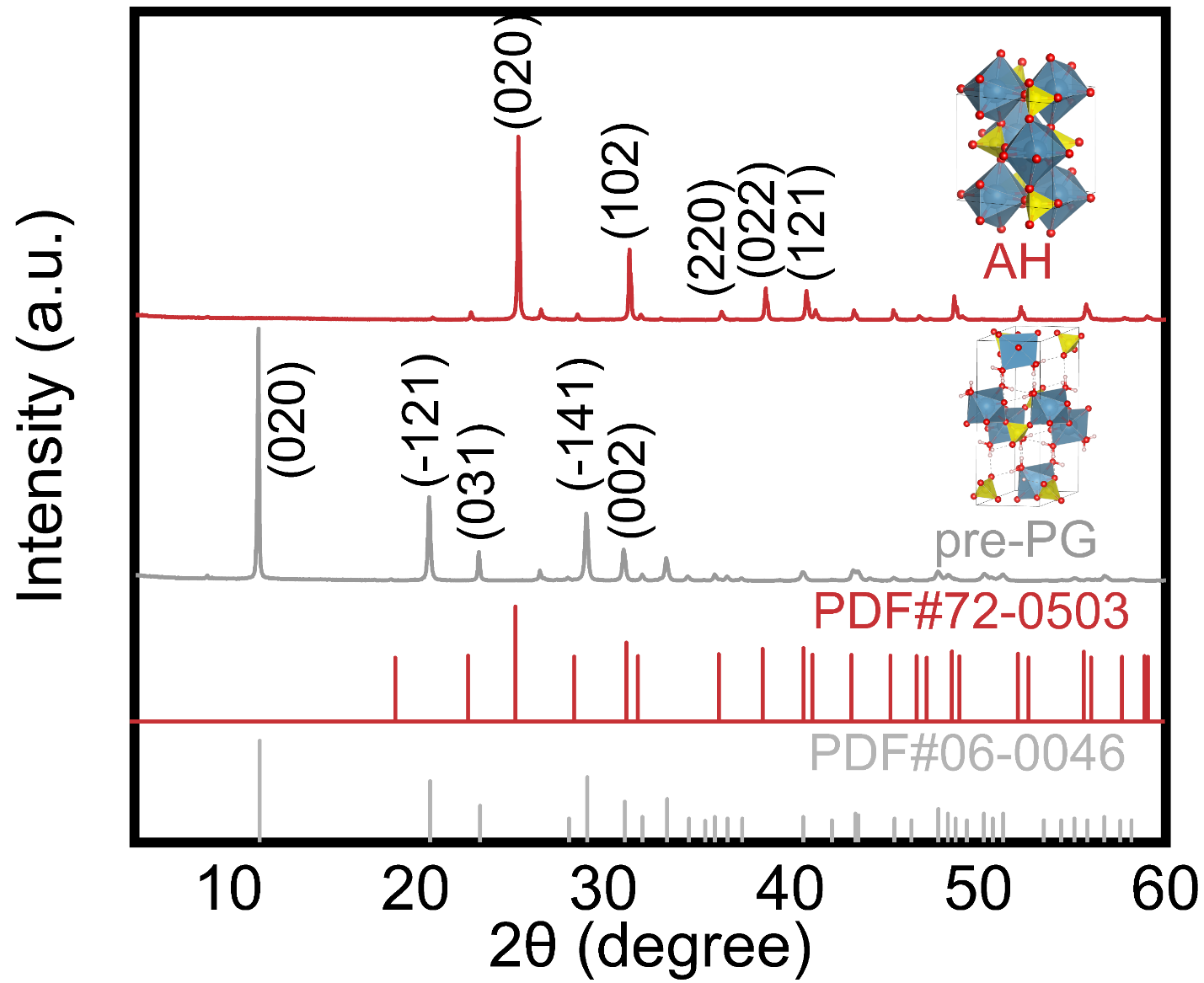


**Figure S1.** XRD patterns and crystal structures of the pre-PG and AH particles. pre-PG shows a typical monoclinic lattice system that matches well with PDF#06-0046, and AH shows a typical orthorhombic lattice system that matches well with PDF#72-0503.


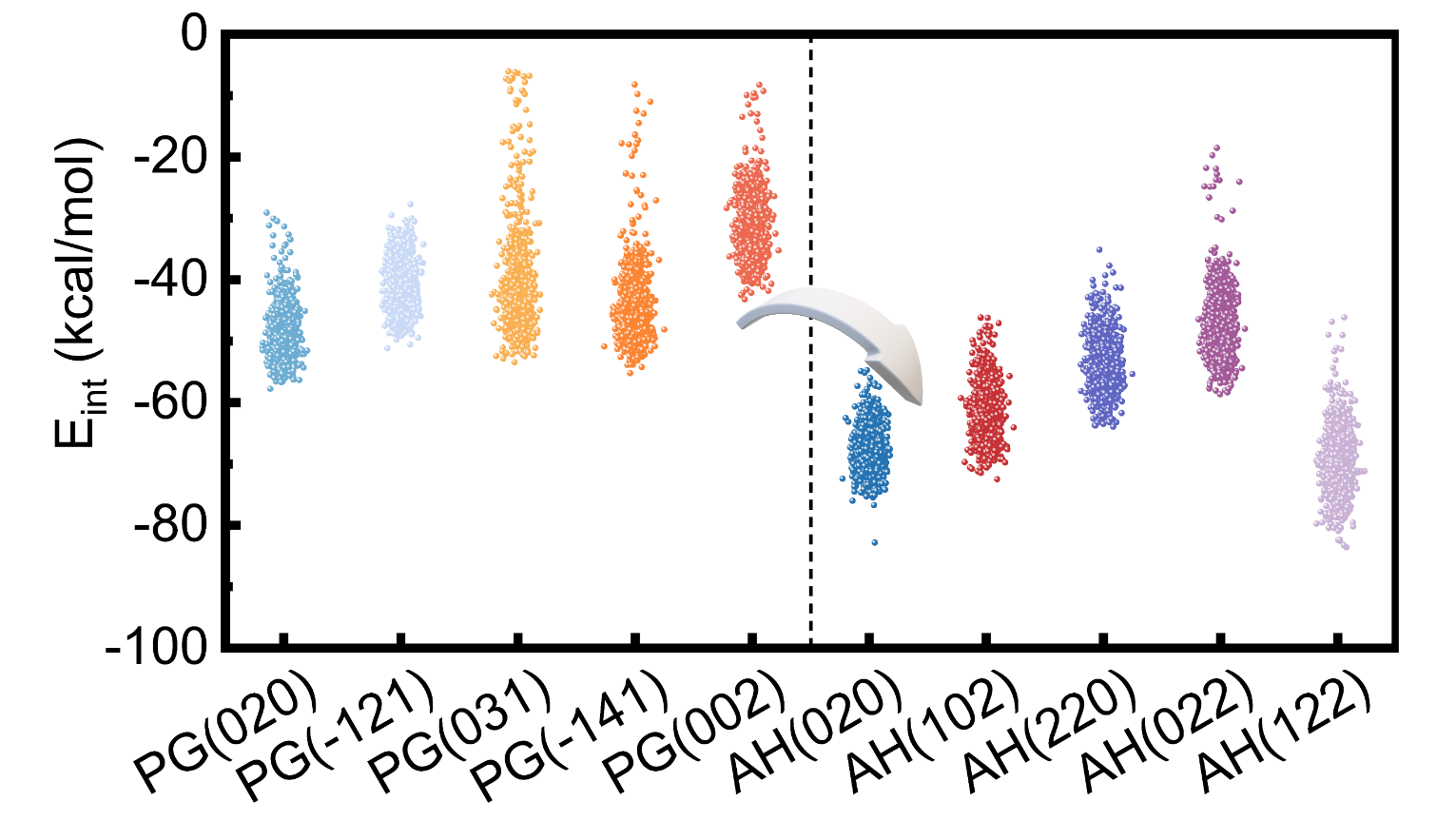


**Figure S2.** the interaction energy values of PG and AH with PP at different facets. The E_int_ of AH major facets with PP molecules are generally higher than those of PG major facets with PP molecules.


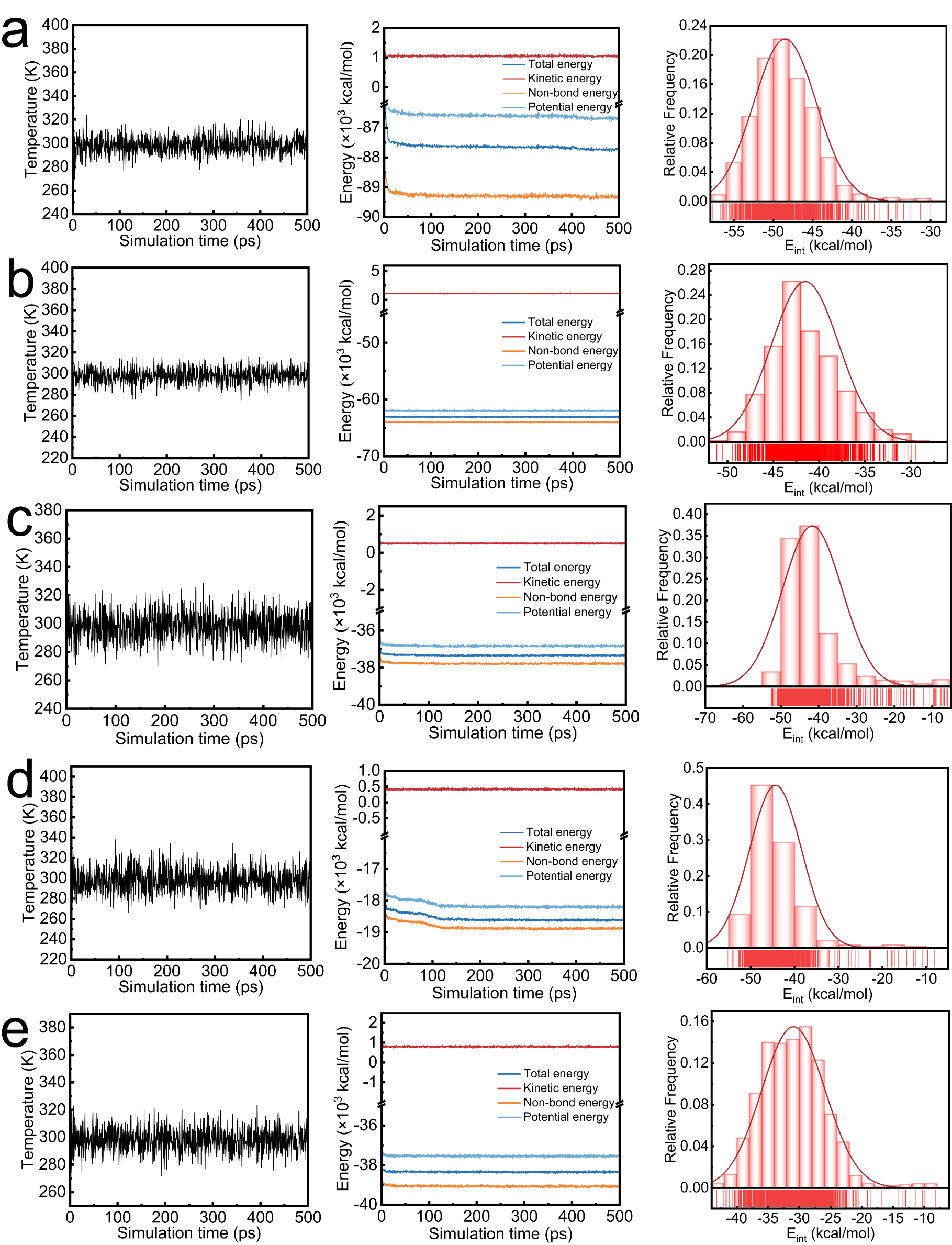


**Figure S3.** The relationship between temperature, different types of energy and simulation time, the relative distribution frequency of E_int_: (a-e) for the (020), (-121), (031), (-141), (002) facets of PG, respectively.


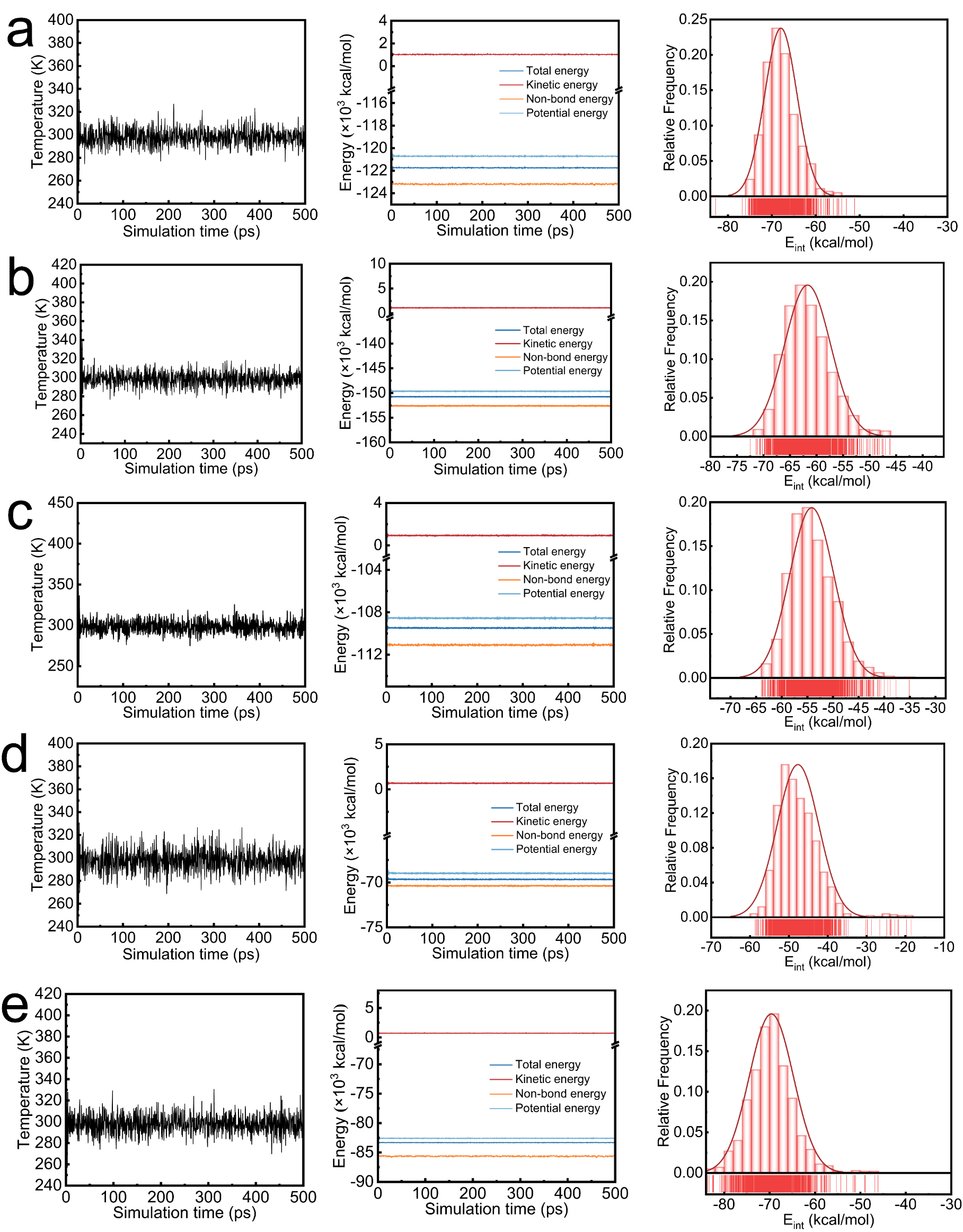


**Figure S4.** The relationship between temperature, different types of energy and simulation time, the relative distribution frequency of E_int_: (a-e) for the (020), (102), (220), (022), (122) facets of AH, respectively.


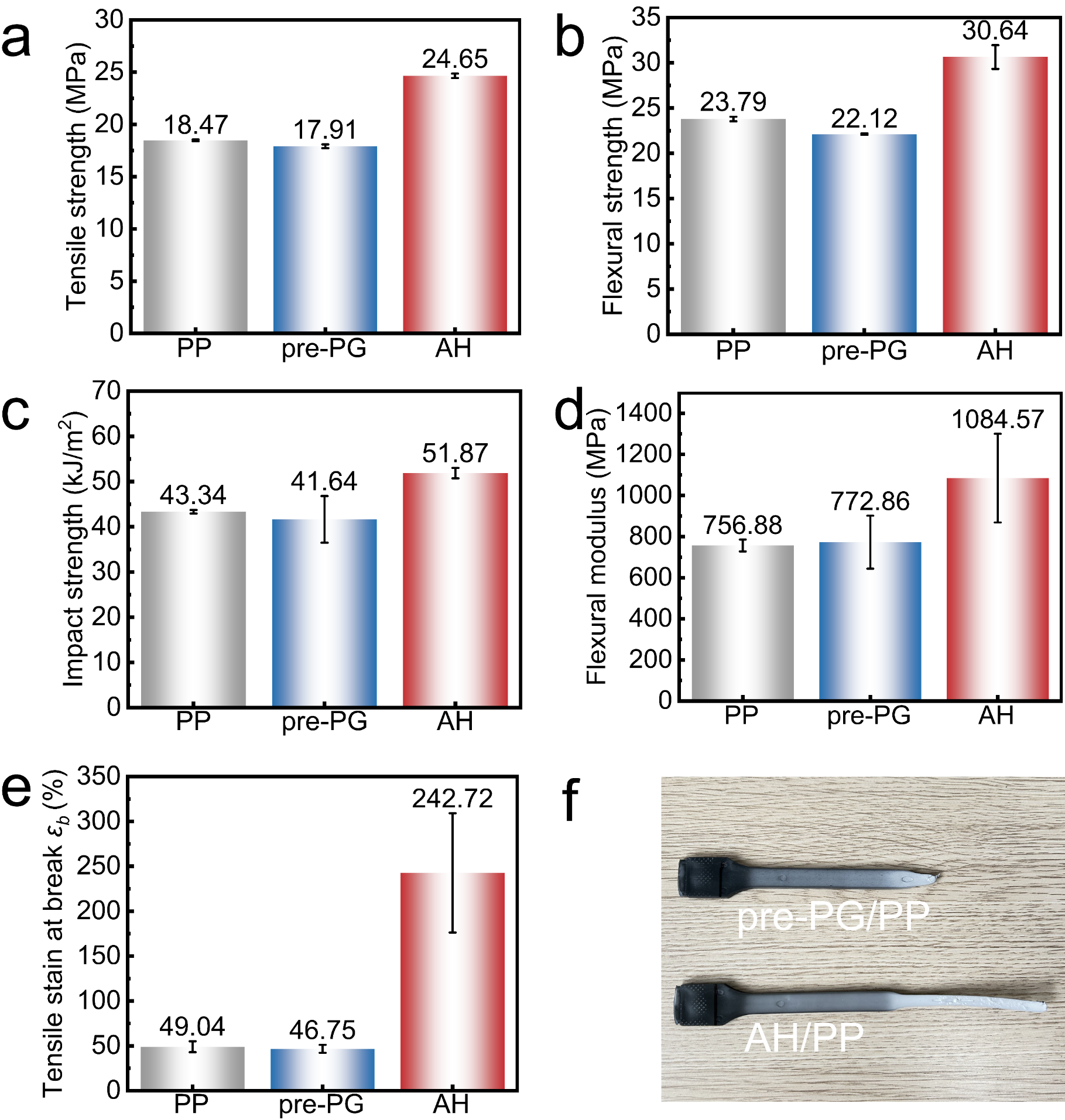


**Figure S5.** The mechanical properties of PG/PP, AH/PP composites and the appearance of tensile fracture.


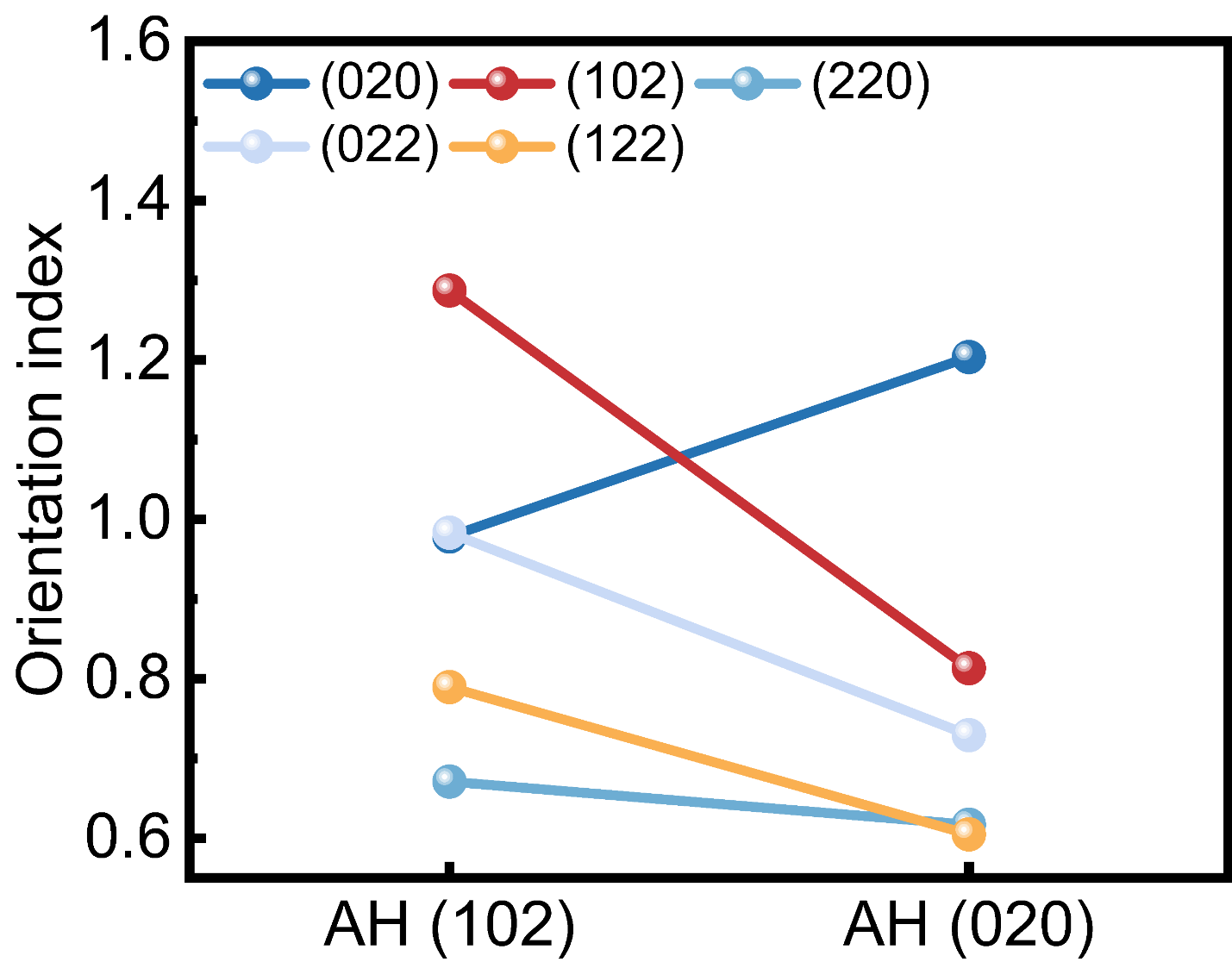


**Figure S6.** The orientation index M of all AH particles. The orientation index M can be used to describe the degree of orientation in a crystal in a particular direction or plane. The larger of the orientation index M, the more pronounced the orientation is in that direction or plane of the crystal. It can be observed that AH (102) has a higher (102) facet orientation and AH (020) has a higher (020) facet orientation.


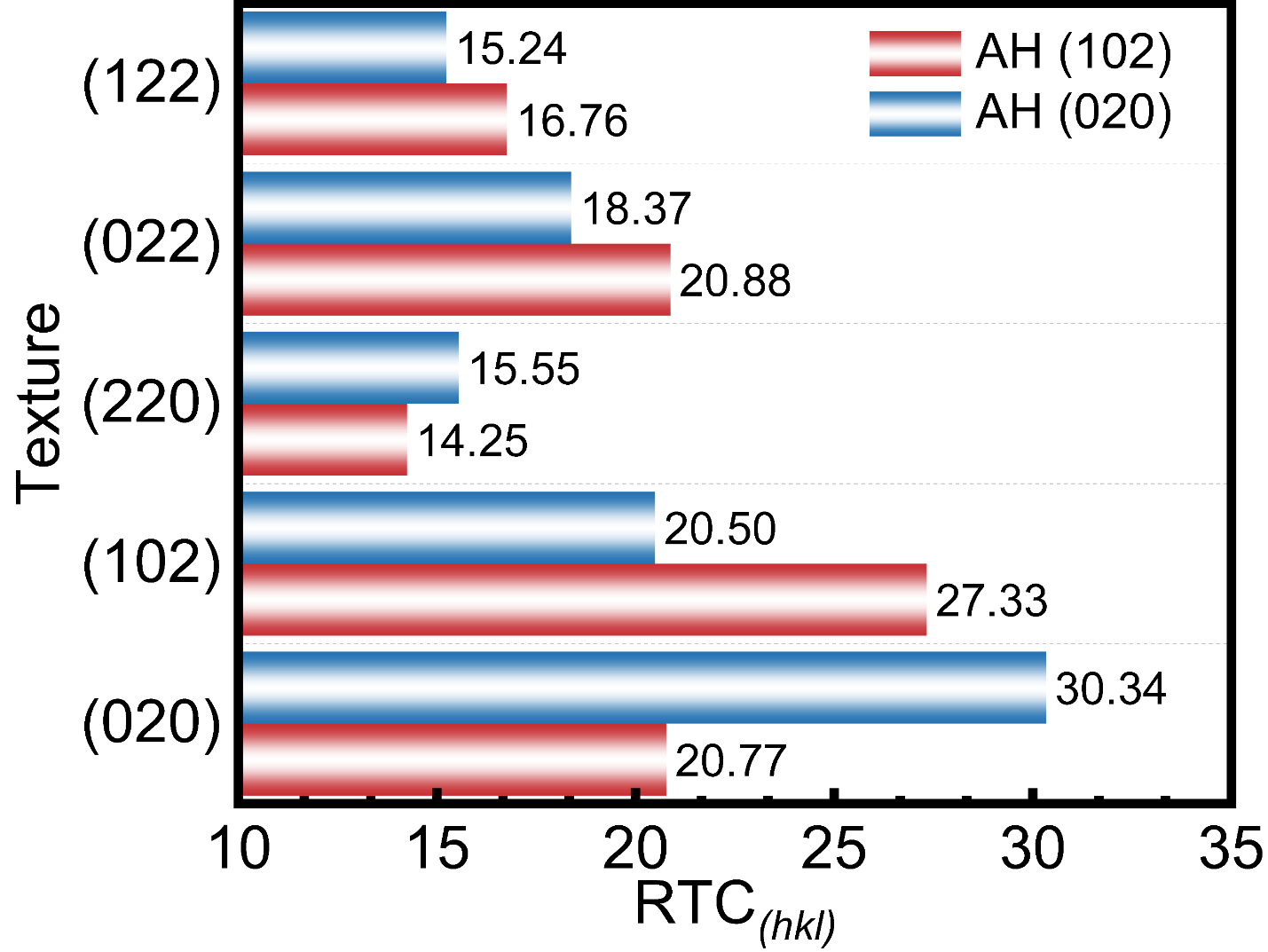


**Figure S7.** The relative texture coefficients ($RTC$s) of all AH particles. The $RTC$ is also an important parameter in describing the orientation distribution of a crystal. It is commonly used to indicate the degree of orientation preference for a particular direction or crystal facet in a crystal. A larger value of the $RTC$ indicates a more pronounced orientation preference for that direction or facet in the crystal. It can be observed that AH (102) has a higher $RTC$ in the (102) facet, and AH (020) has a higher $RTC$ in the (020) facet.


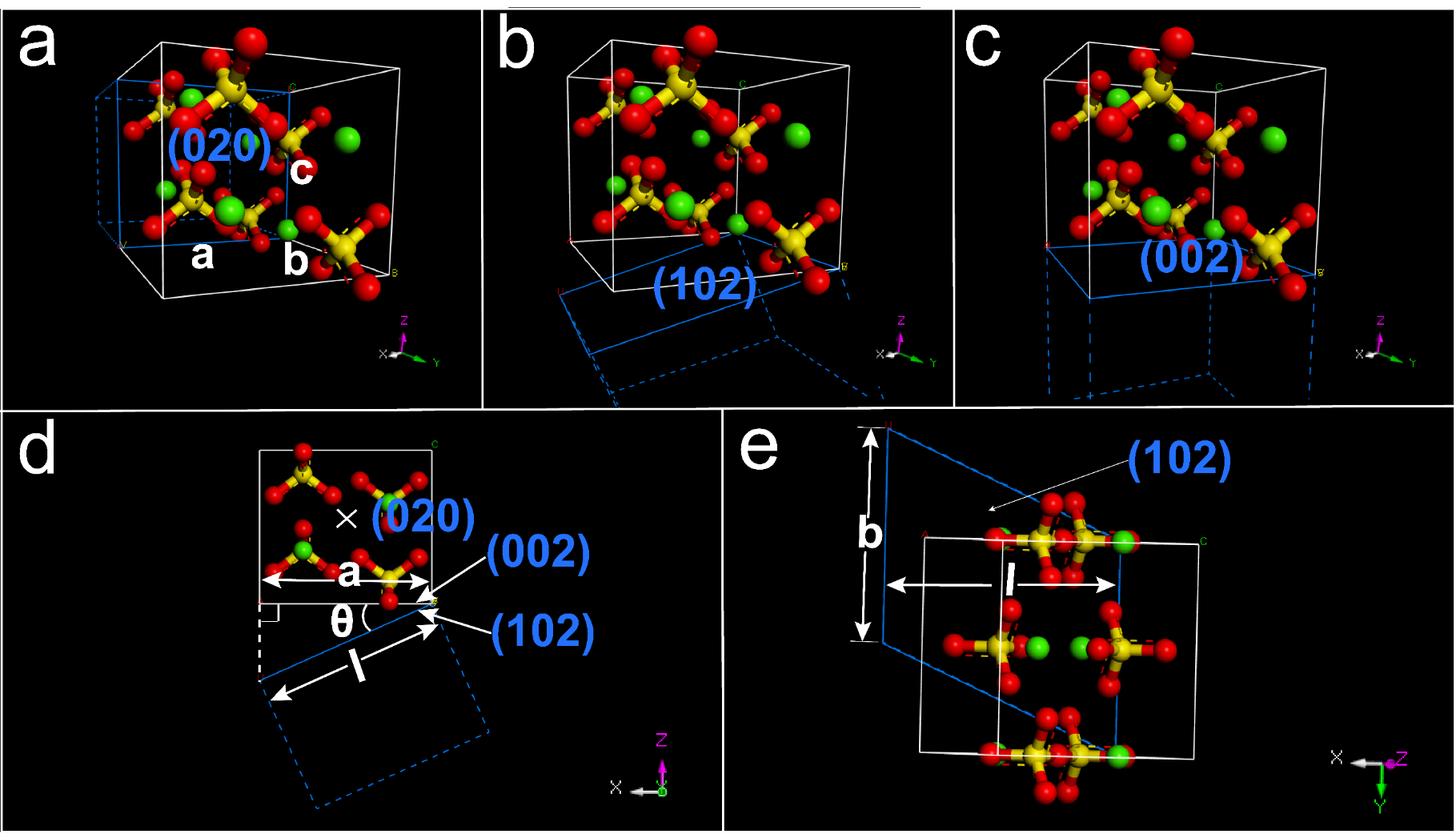


**Figure S8.** (a), (b), (c) are the schematic diagrams of (020), (102) and (002) facets, respectively, (d) The schematic diagram of the calculation of *l* and (e) The schematic diagram of the calculation of $S_{102}$.


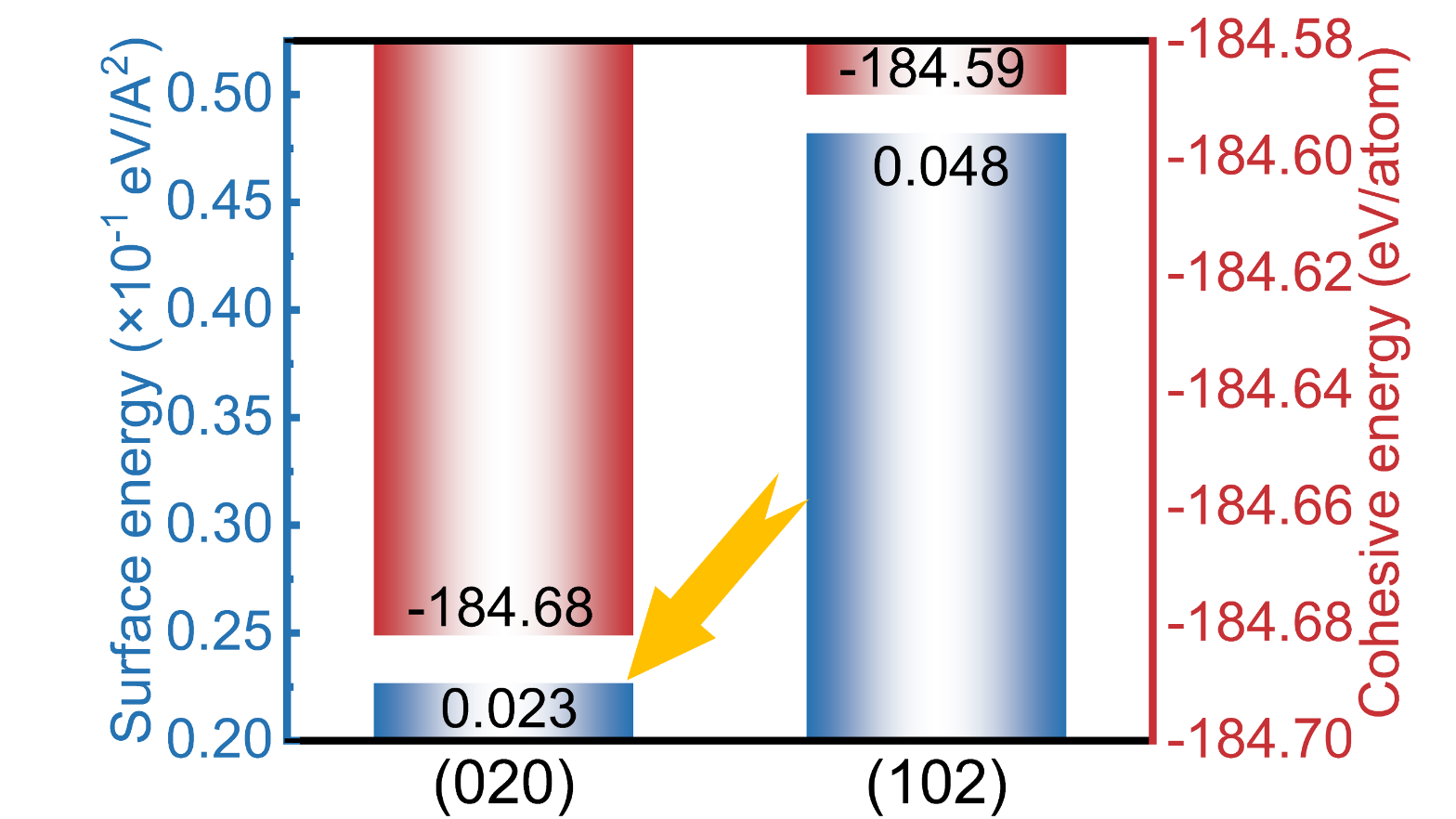


**Figure S9.** The surface energy and cohesive energy of (020) and (102) facets. When the surface energy is high, the crystals will tend to lower the surface energy, which may lead to changes in the surface structure of the crystals or even the formation of surface defects or changes in morphology to lower the total energy. Under natural conditions, the fact that the (102) facet has a higher surface energy means that the (102) facet in AH may be more inclined to transform to the (020) facet, which is why AH with the (102) facet as the dominant facet rarely exist in nature. The magnitude of the cohesive energy of a crystal depends on factors such as the structure, composition, and lattice parameters of the crystal. The level of cohesive energy directly affects the physical properties of crystals such as melting point, hardness, modulus of elasticity, as well as the thermal and chemical stability of crystals. The higher cohesive energy of the (020) facet confirms the above conclusion that the (102) facet is more unstable and easily converted into the (020) facet in natural conditions.


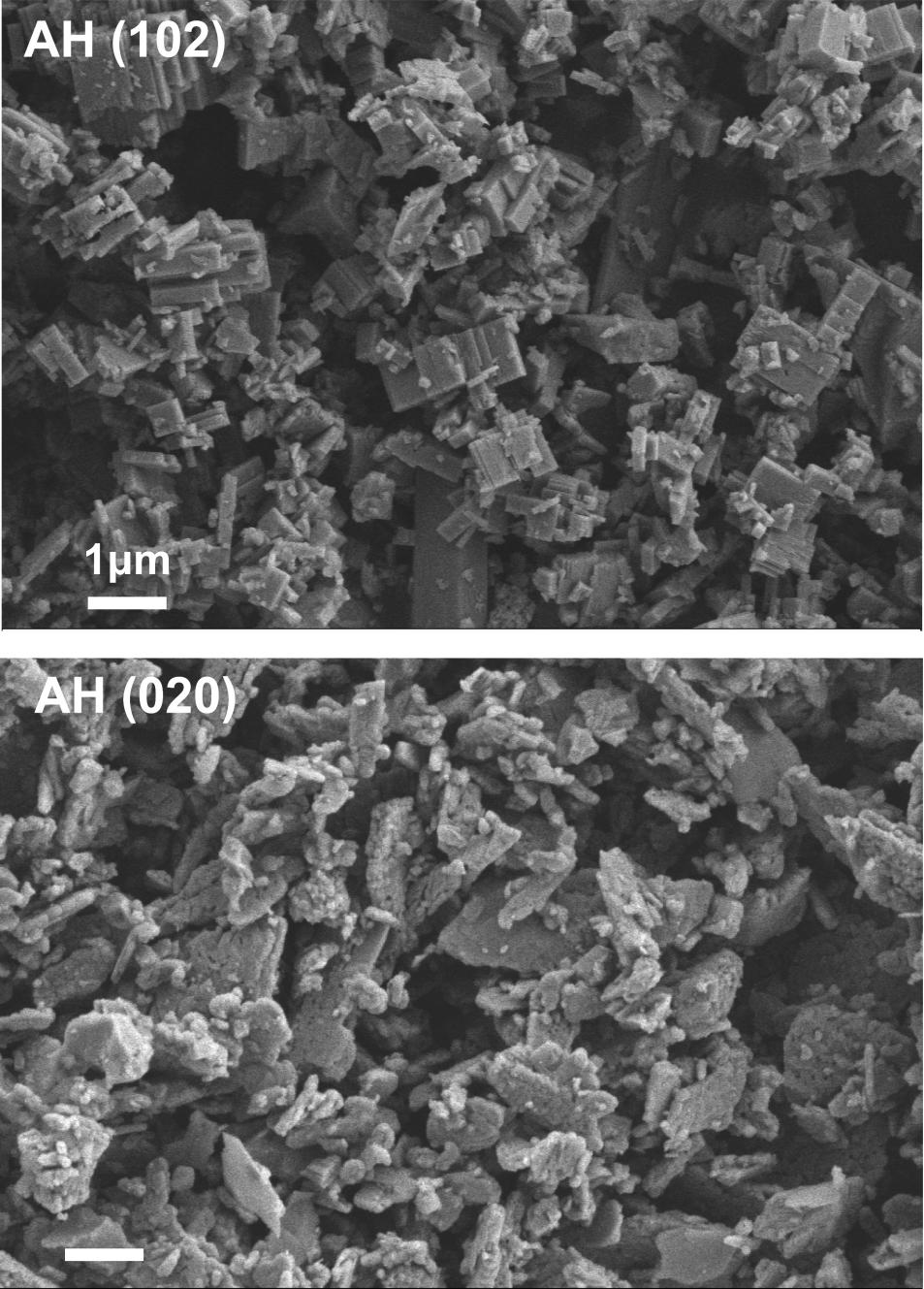


**Figure S10.** The SEM images of the AH particles. As with the results shown by TEM, the AH (102) sample presents a more regular rectangular shape while the AH (020) sample presents a random shape.


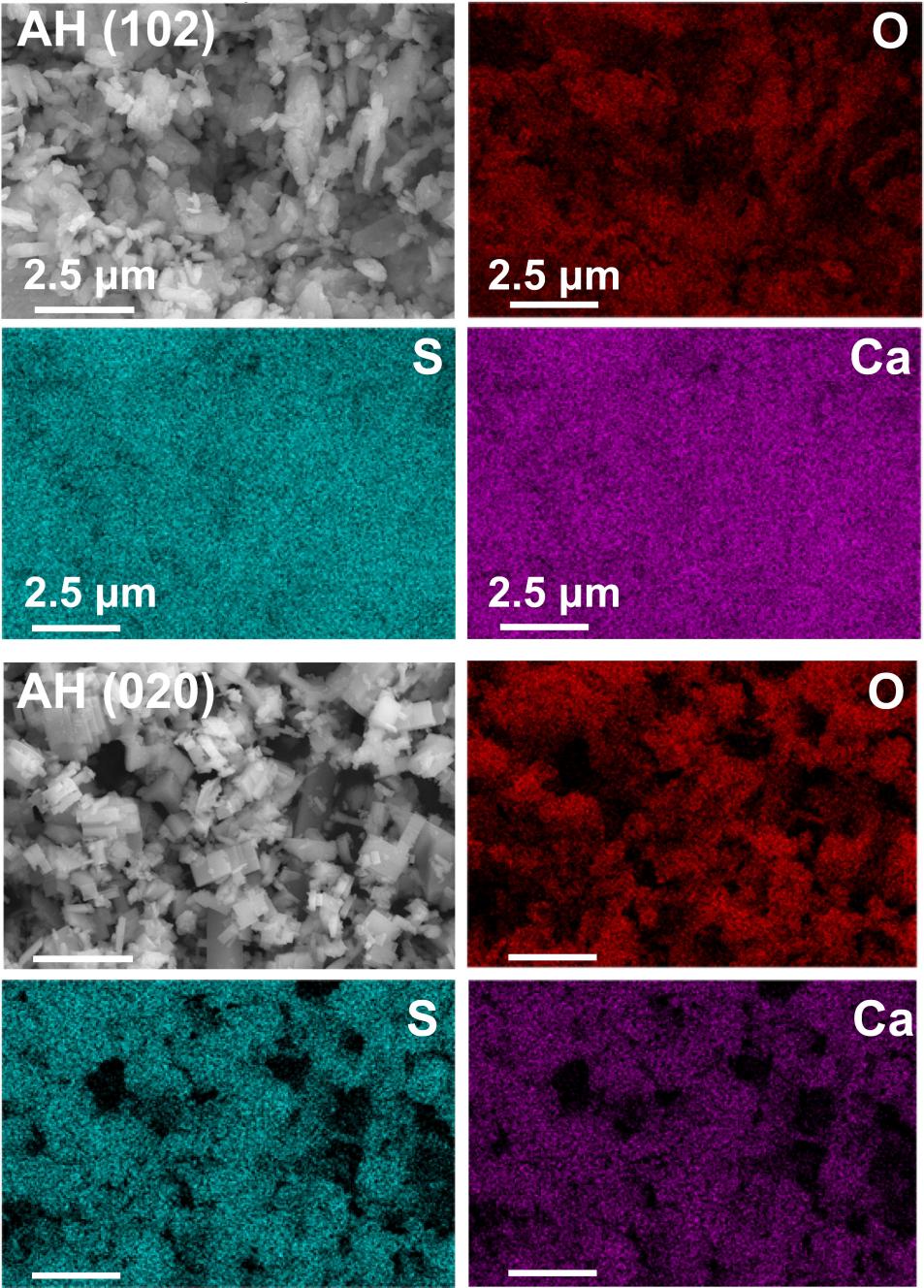


**Figure S11.** The SEM-EDS images of the AH particles. The EDS images show good distribution of elements within the samples.


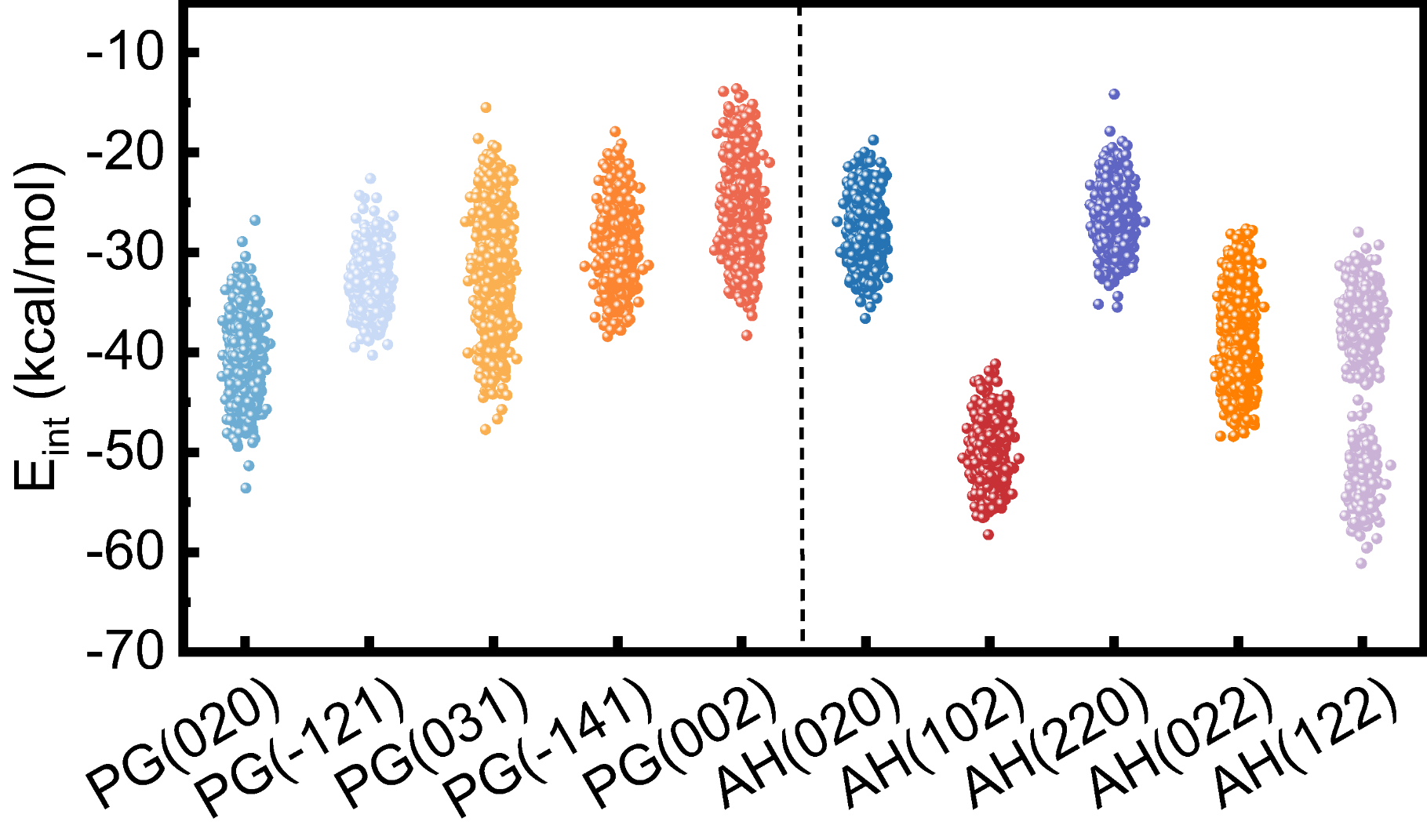


**Figure S12.** The E_int_ between the facets of PG and AH with H_2_O molecule in the simulation time of 500 ps. The E_int_ between water molecules and the primary facets of AH, particularly the (102) facet, are notably greater than those between water molecules and the facets of PG. Moreover, they surpass the E_int_ between water molecules and the (020) facet of AH.


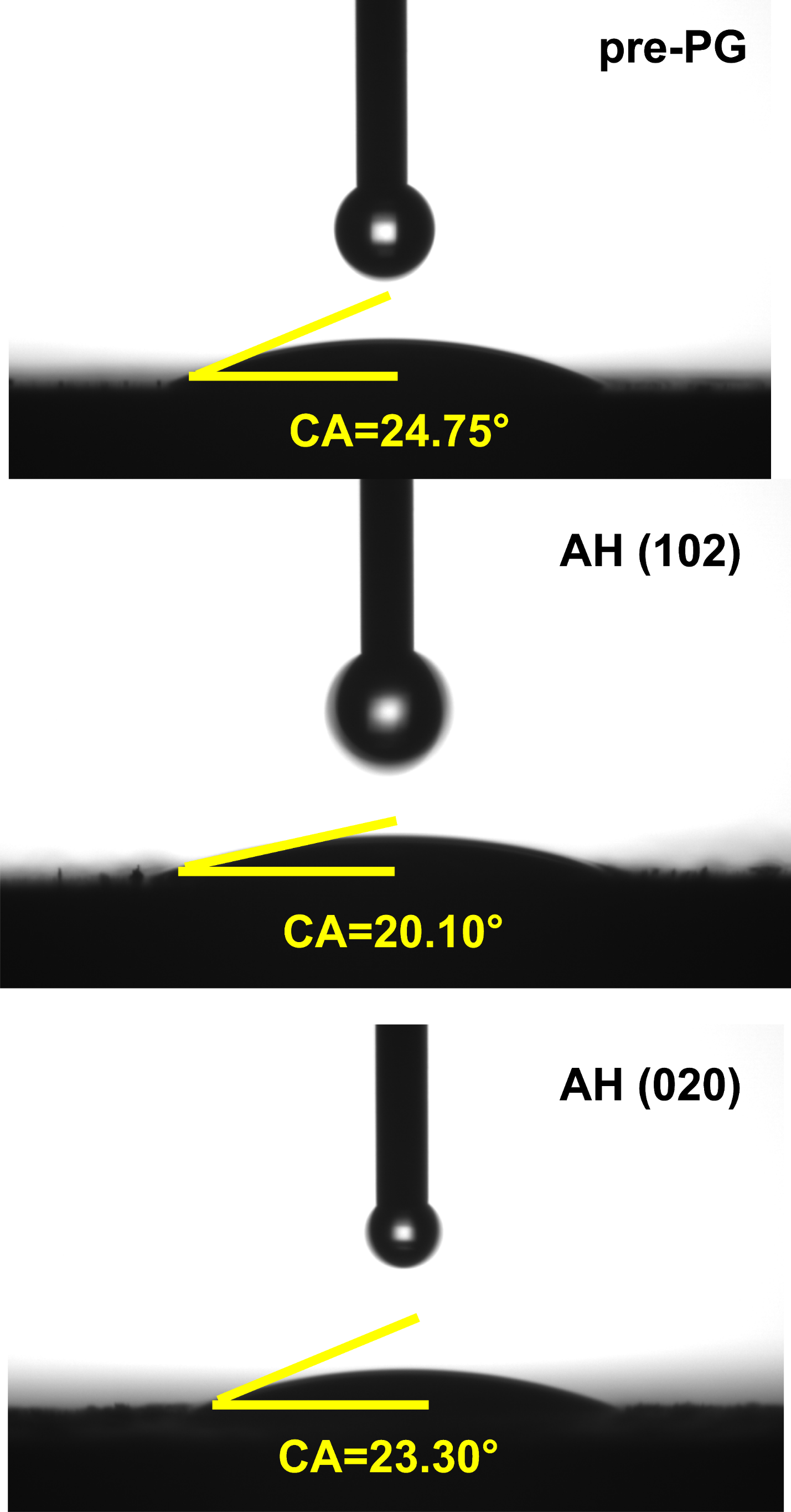


**Figure S13.** The contact angle of flakes made of pre-PG, AH (102) and AH (020) particles with H_2_O. The minimal contact angle observed on the AH (102) sample in water validates the precision of the E_int_ calculations for each facet with water molecules. This observation further supports the notion that the AH (102) exhibits a higher proportion of (102) facet exposure.


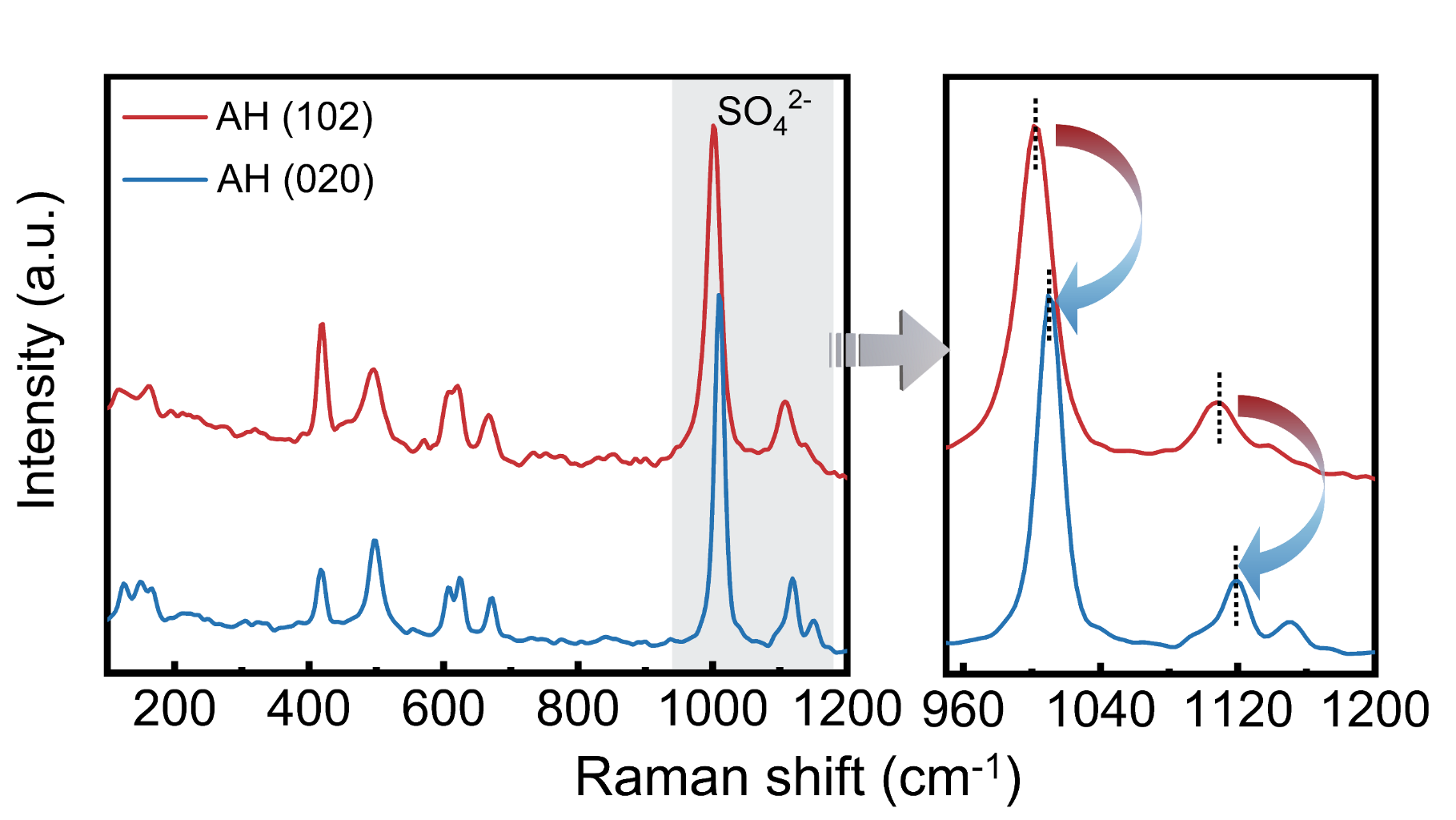


**Figure S14.** The Raman curves of AH particles. It shows the variation of the Raman spectrum in the low-shift region of all samples. The AH produces peaks at 1003 and 1116 cm^-1^ due to the ν1 symmetric stretch and ν3 asymmetric stretch modes of SO_4_ tetrahedra, respectively. In the case of sulfates of the two AH particles with similar structure, the wavenumber of the vibrational modes of the SO_4_ tetrahedra, increases with a decrease of the exposure ratio of the (102) facet. Different exposure ratios of (020) or (102) facet lead to distortion of the SO_4_ tetrahedra.^[21]^ As the S-O bond stretching force constant increases or decreases, the Raman peaks move to higher or lower wave numbers.^[22]^


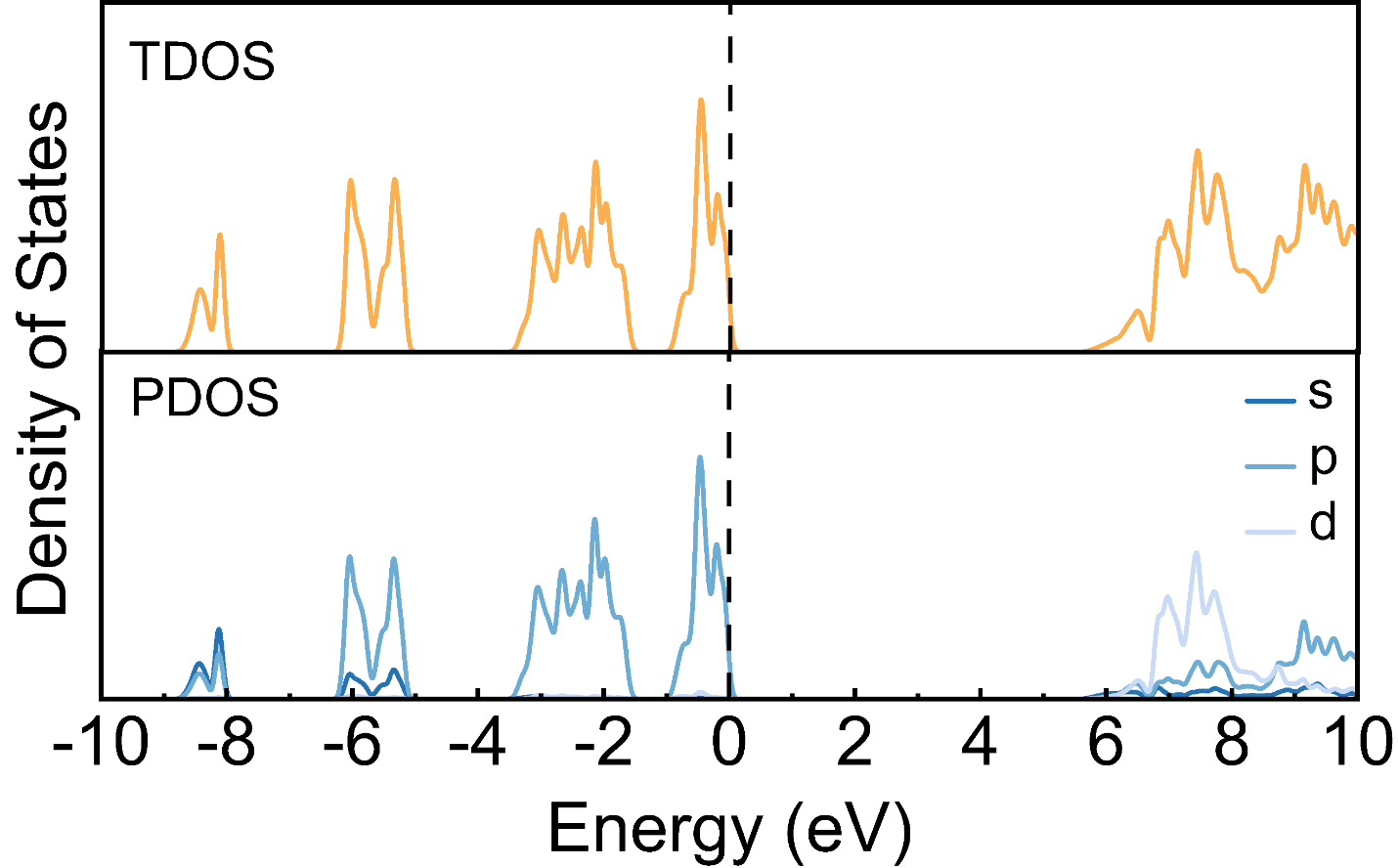


**Figure S15.** Total density of state (TDOS) and partial density of state (PDOS) for (020) facet. **Figure S15** and **S16** are the fits to the TDOS of the two crystal planes, respectively. In both calcium sulfate systems, there is a wider band gap below its Fermi energy level. This means that it is more difficult to move electrons into the conducting band and further participate in the reaction in calcium sulfate, presenting a clear insulator property. The valence band of both systems consists mainly of s and p orbitals, and the conduction band consists mainly of p and d orbitals, with a partial contribution from s orbitals.


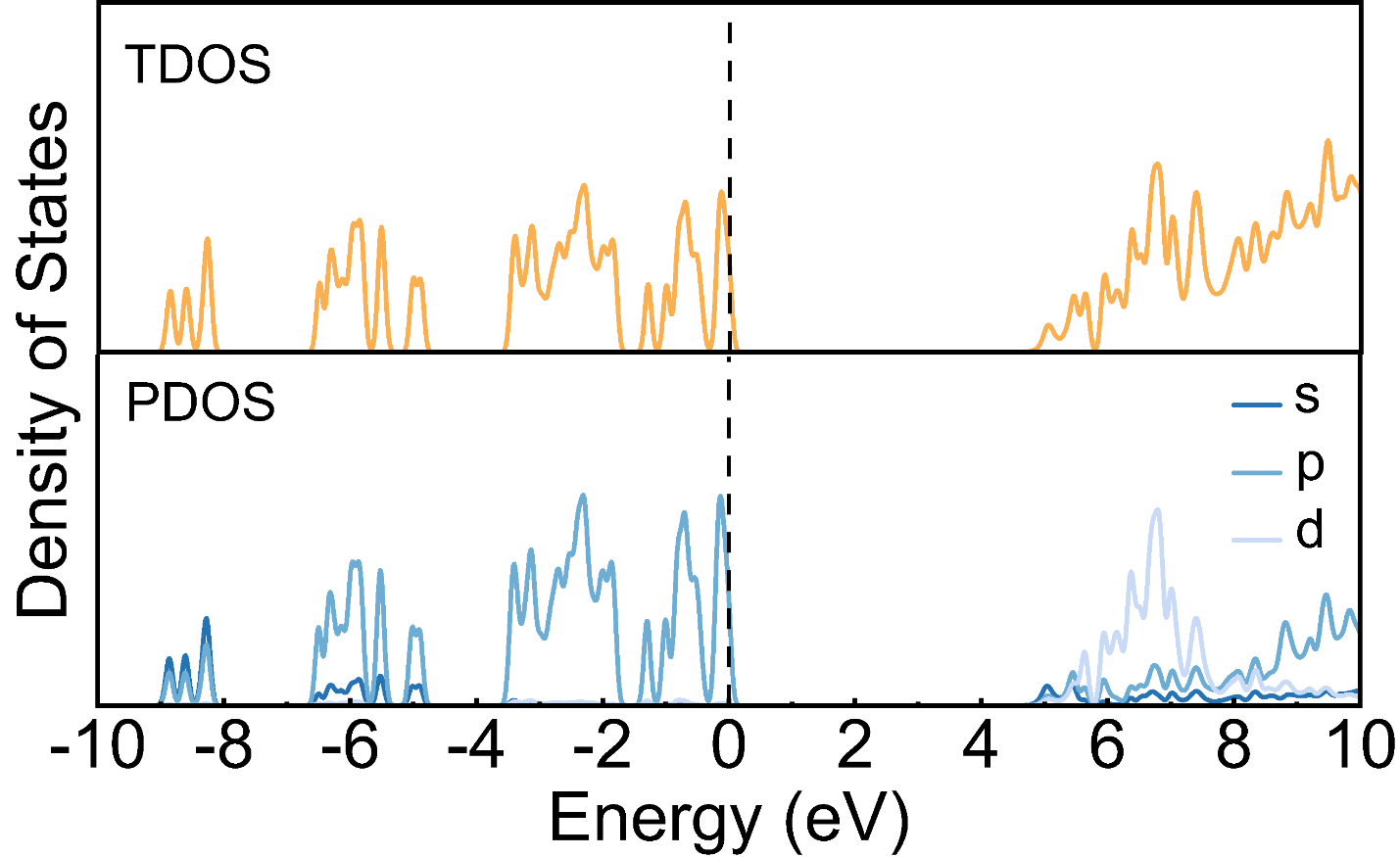


**Figure S16.** Total density of state (TDOS) and partial density of state (PDOS) for (102) facet.


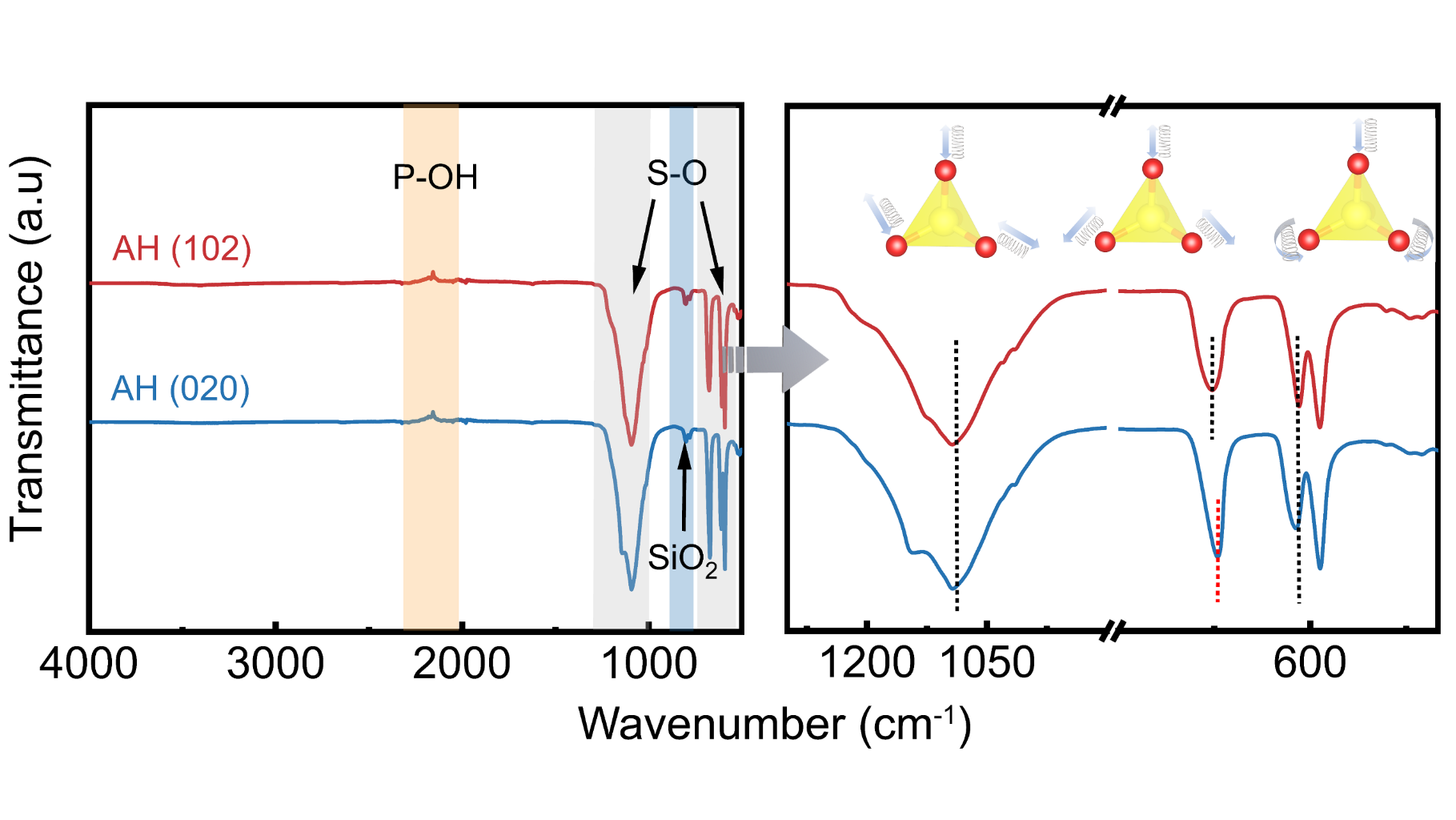


**Figure S17.** The FTIR curves of AH particles. The absorptions at ≈1100, 669, and ≈610 cm^-1^ are attributed to the asymmetric and symmetric S-O stretching vibrations of SO_4_ and the O-S-O bending vibrations of SO_4_. The band at 798 cm^-1^ is attributed to the vibration of Si-O-Si.^[23]^ The S-O vibrational absorption bands at approximately 669 cm^-1^ shifts to a lower wavenumber as the exposure ratio of the (102) facet decreases, and it shifts to a higher wavenumber as the exposure ratio of the (102) facet increases, which is attributed to the electron absorption induced effect of O atoms in CaSO_4_ on different facets


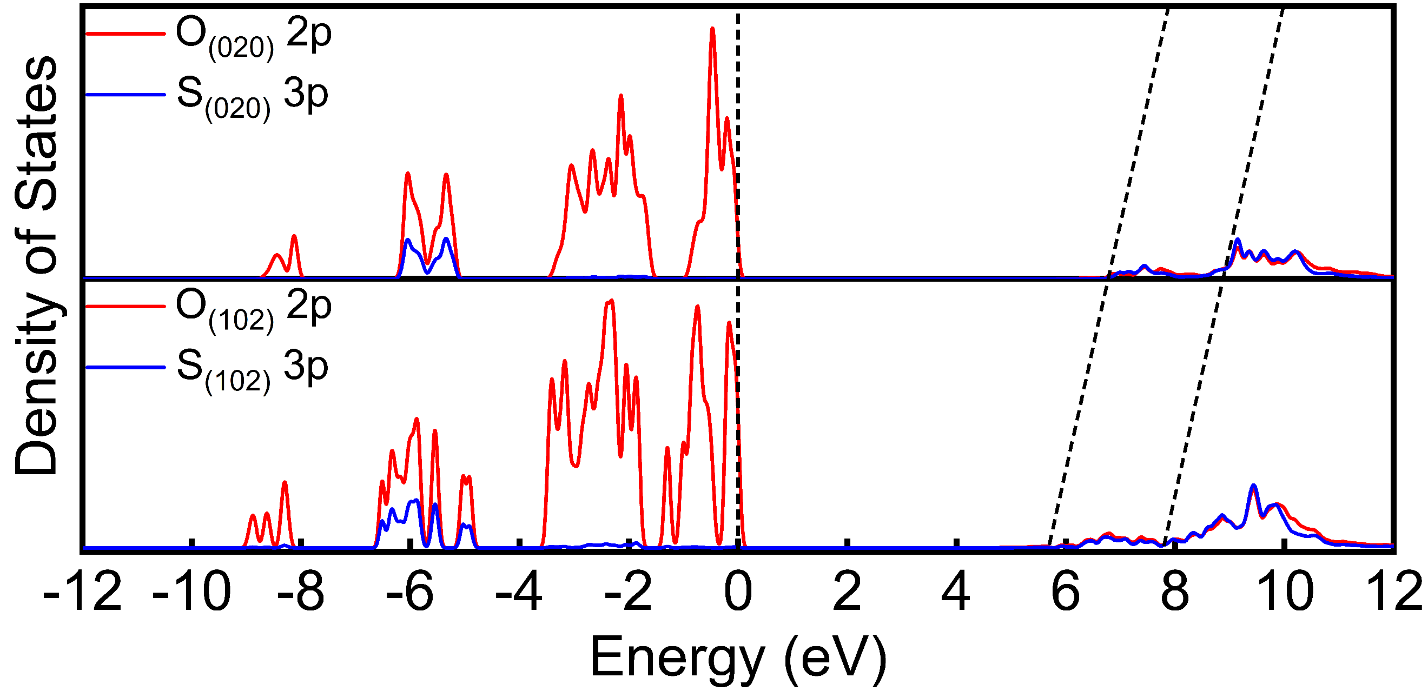


**Figure S18.** PDOS of O and S atoms in (020) and (102) facets.


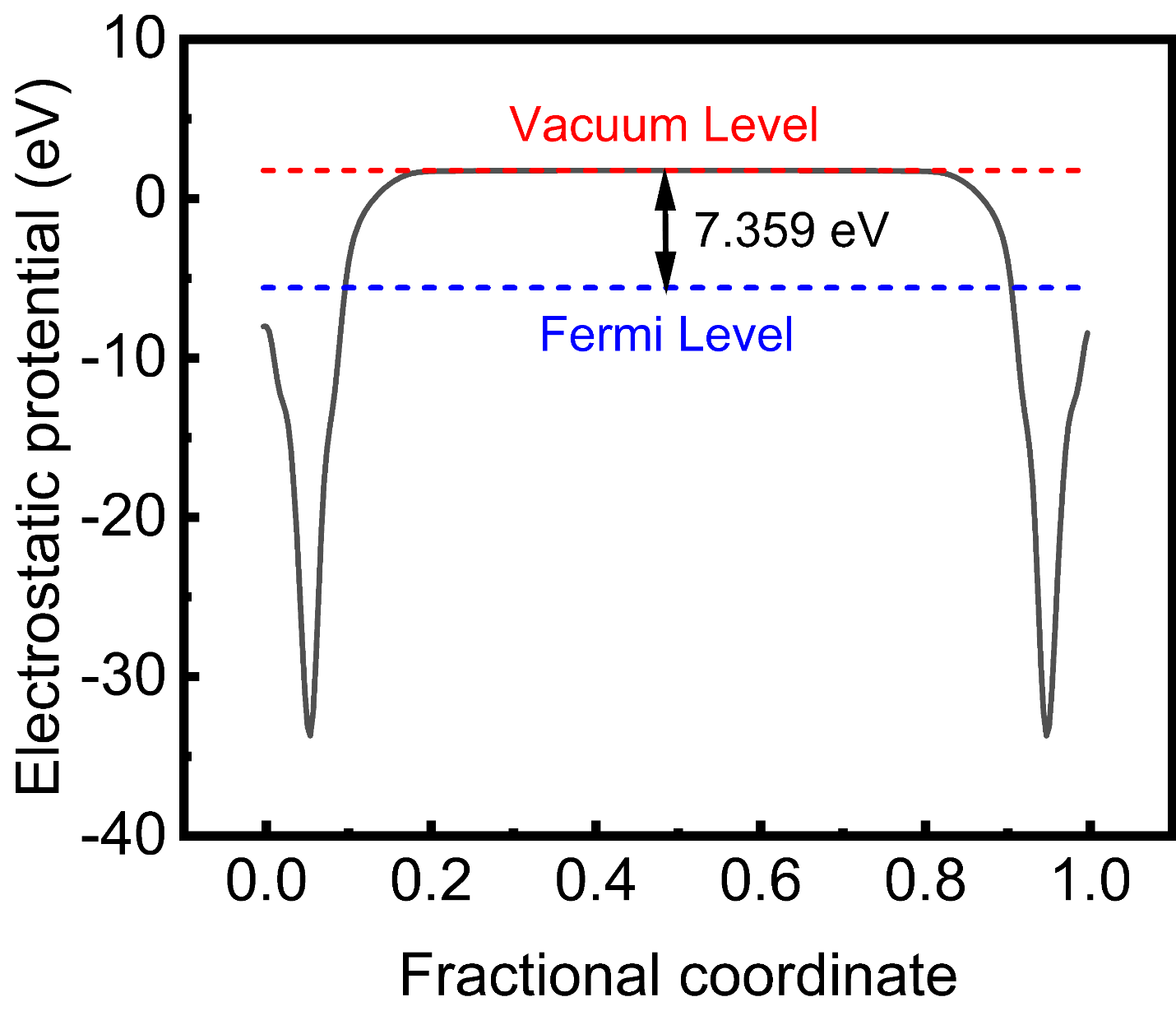


**Figure S19.** Calculated work functions (Φ) of (020) facet.


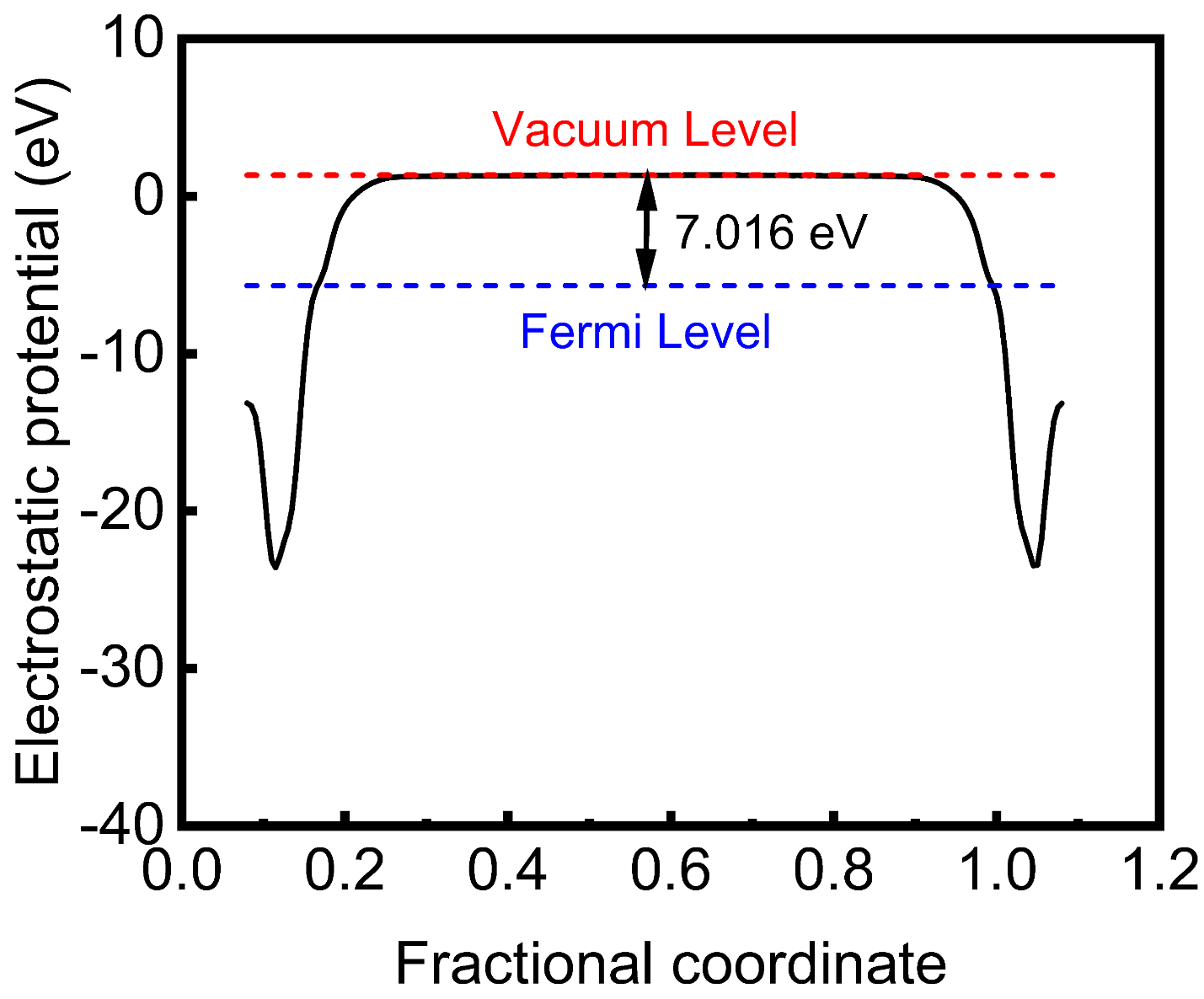


**Figure S20.** Calculated work functions (Φ) of (102) facet.


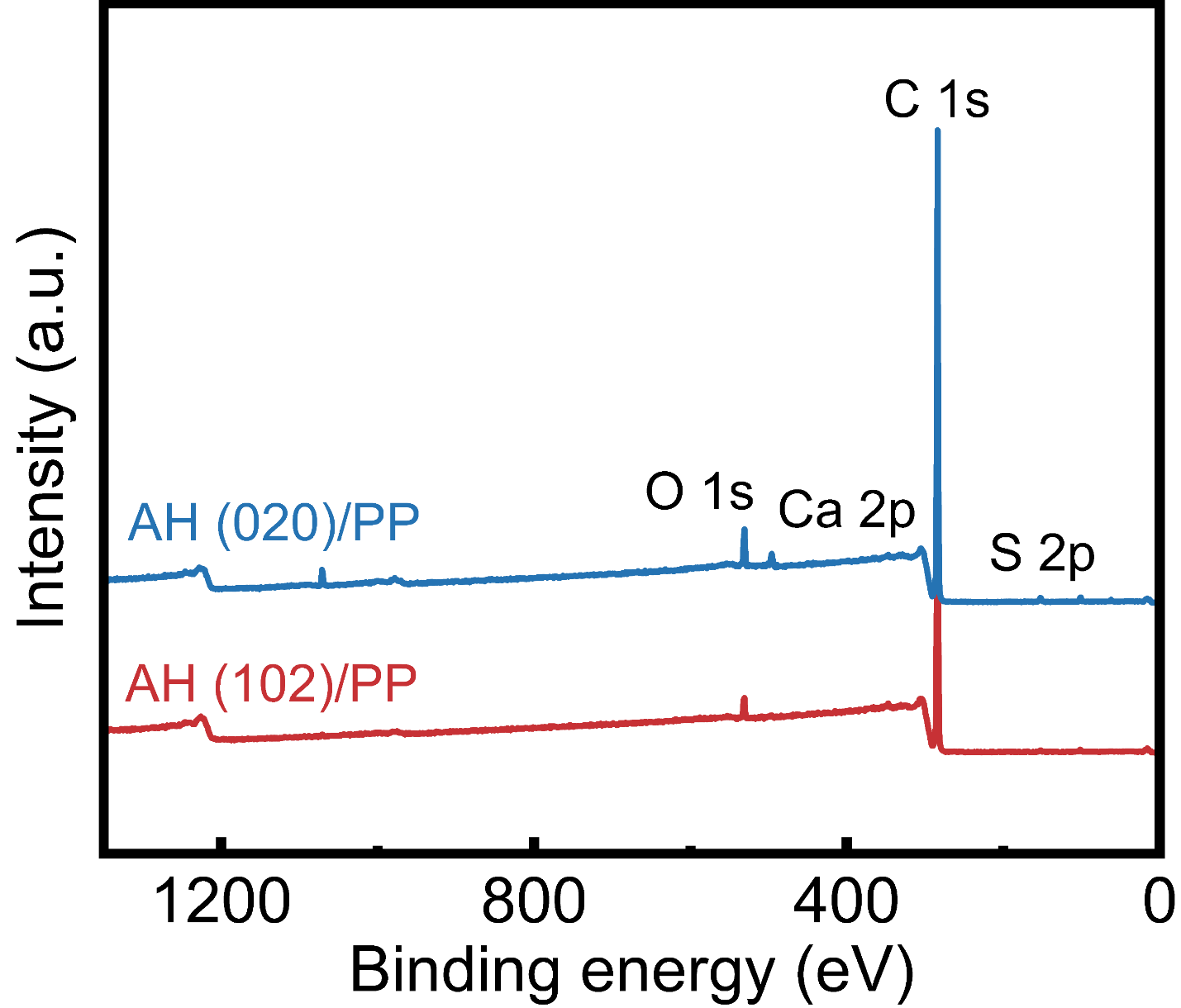


**Figure S****21.** The XPS patterns of the AH/PP composites. The wide-scan XPS spectra showed that the chemical composition of AH was Ca, S, O and that of the AH/PP composite was Ca, S, O, C, which agreed with the XRD, XRF and EDS result. The C1s peak presents at ≈284.80 eV.^[24]^ The photoelectron peak assigned to C 1s is significantly enhanced in the XPS spectra of AH/PP composites, indicating that the PP is well distributed on the surface of AH particles.


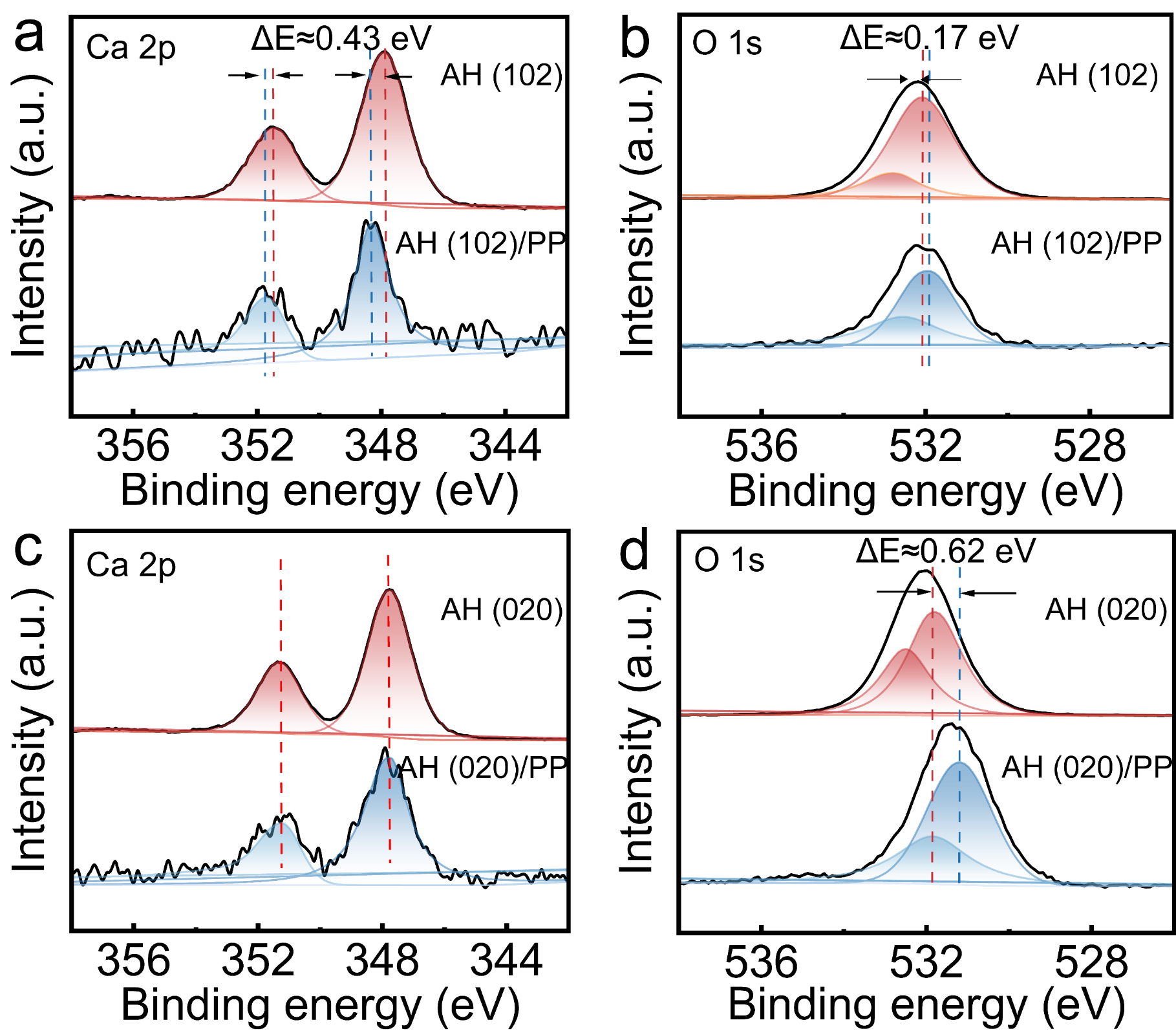


**Figure S22.** The XPS spectra of Ca 2p peak of the AH and AH/PP composites. The high-resolution XPS spectra of Ca 2p and O 1s. Ca 2p has two peaks centered at ≈351.48 eV (Ca 2p1) and ≈347.88 eV (Ca 2p3).^[25]^ With respect to O 1s spectra, the peak was composed of two contributions at ≈532.78 eV and≈532.08 eV, which represented Si-O and S-O.^[26]^ The binding energy of Ca 2p in AH before and after composite with PP did not change significantly with increasing exposure ratio of the (020) facet, indicating that PP has little effect on the chemical state of Ca atoms in the (020) facet. The binding energy of Ca 2p in AH increased with the increase of the exposure ratio of the (102) facet.


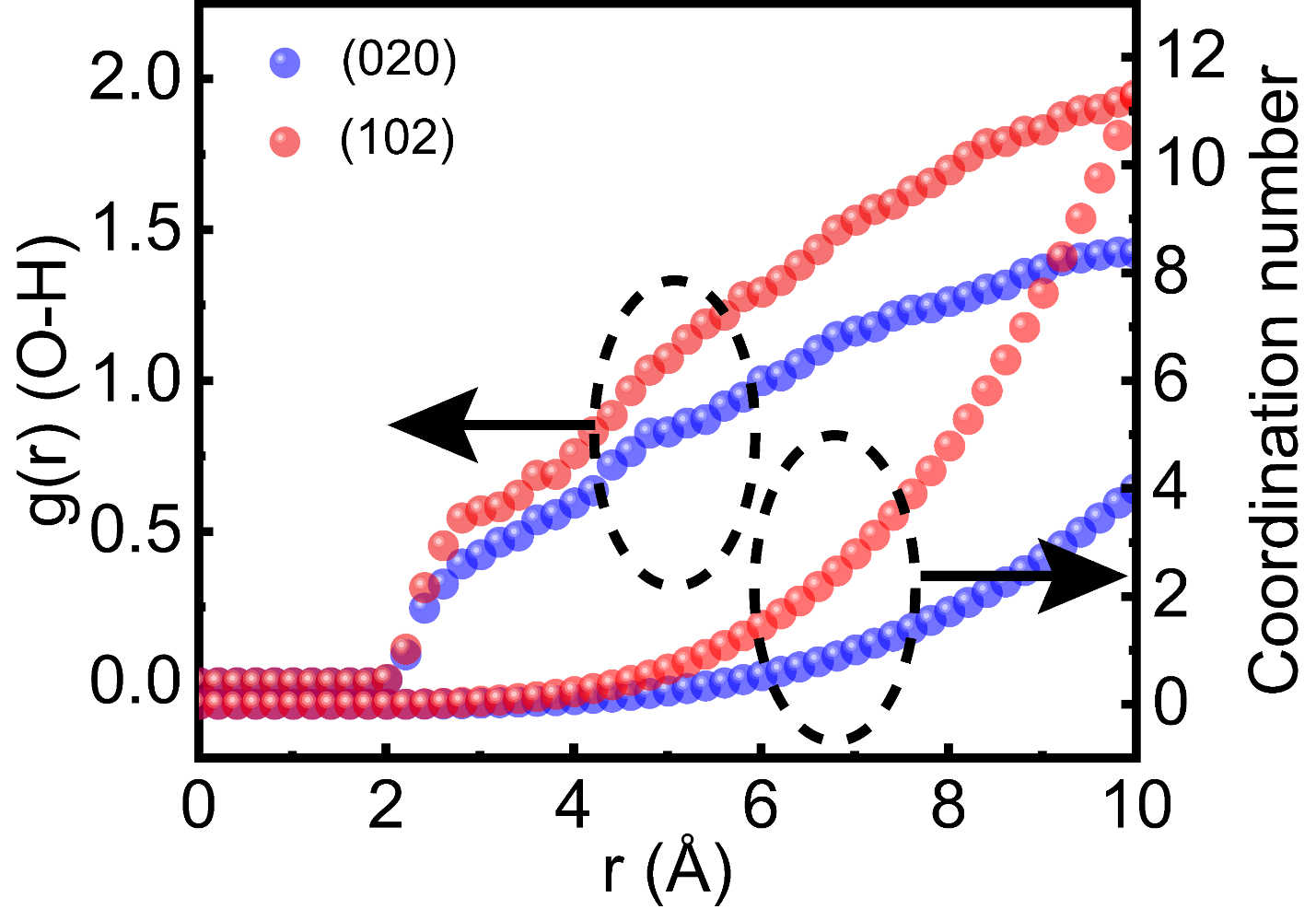


**Figure S23.** The RDF and C-N curves of O-H in AH-PP composites.


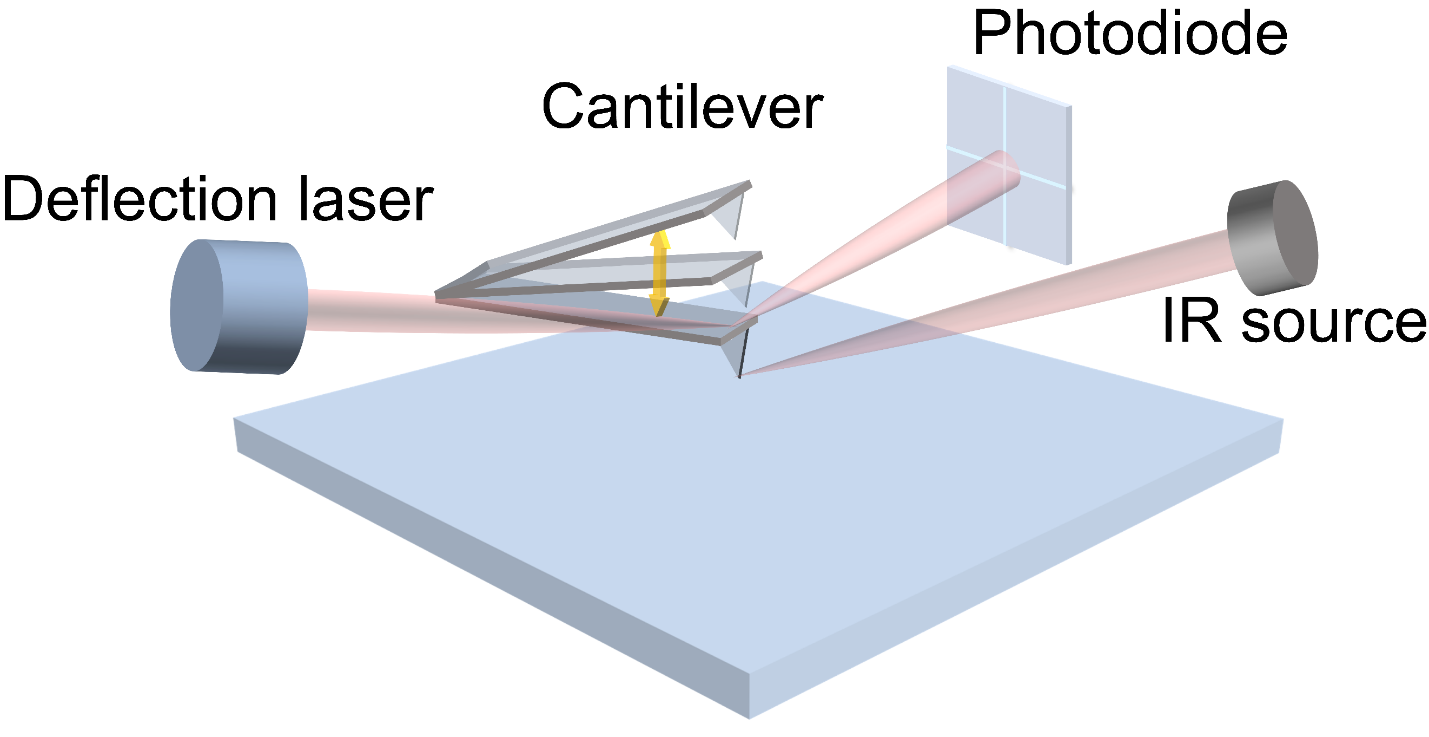


**Figure S24.** Schematic illustration of the AFM-IR measurement.


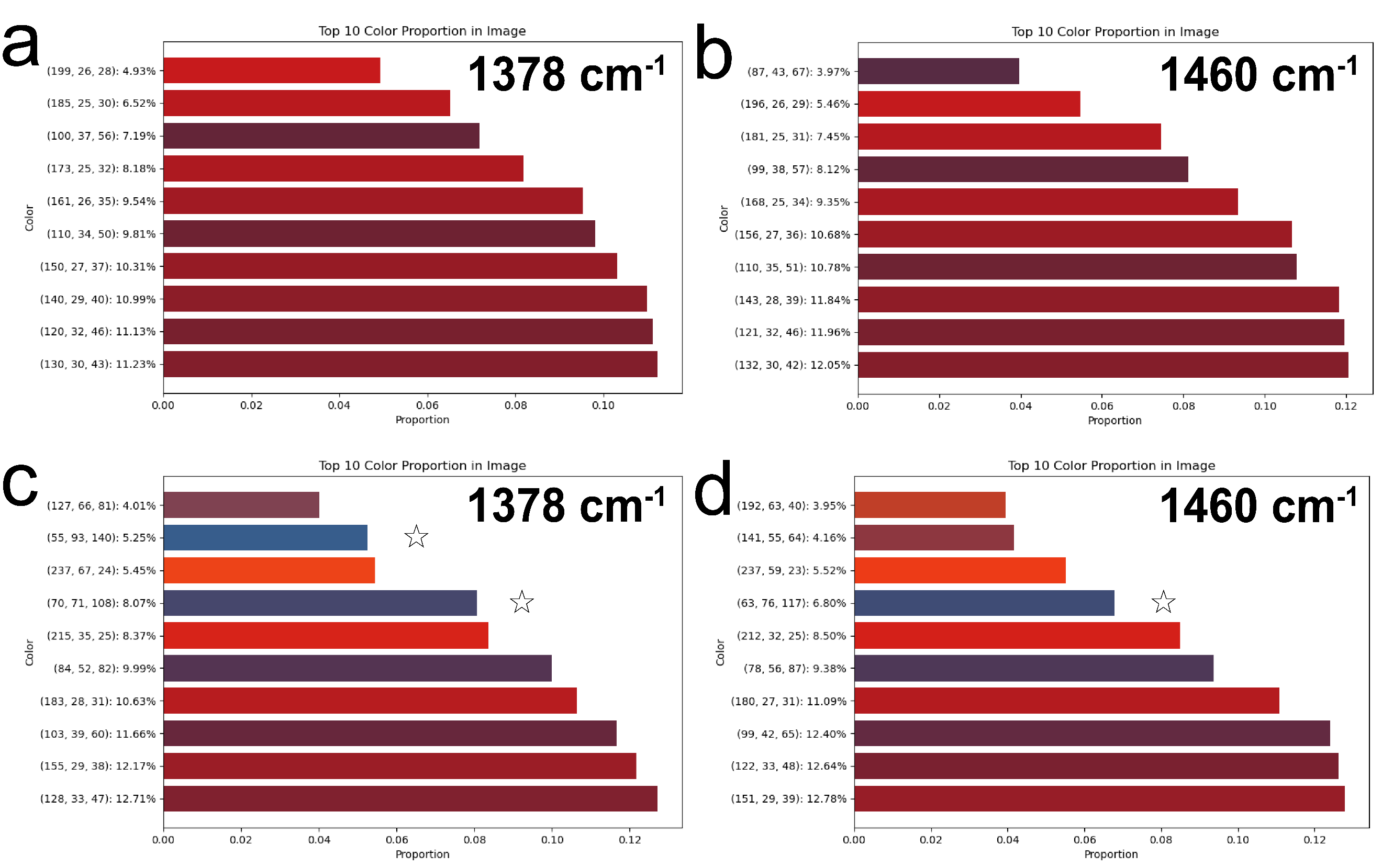


**Figure S25.** Color quantification of AFM-IR chemical maps using Python involves displaying the top 10 colors and their proportions: (a-b) AH (102)/PP and (c-d) AH (020)/PP composite.


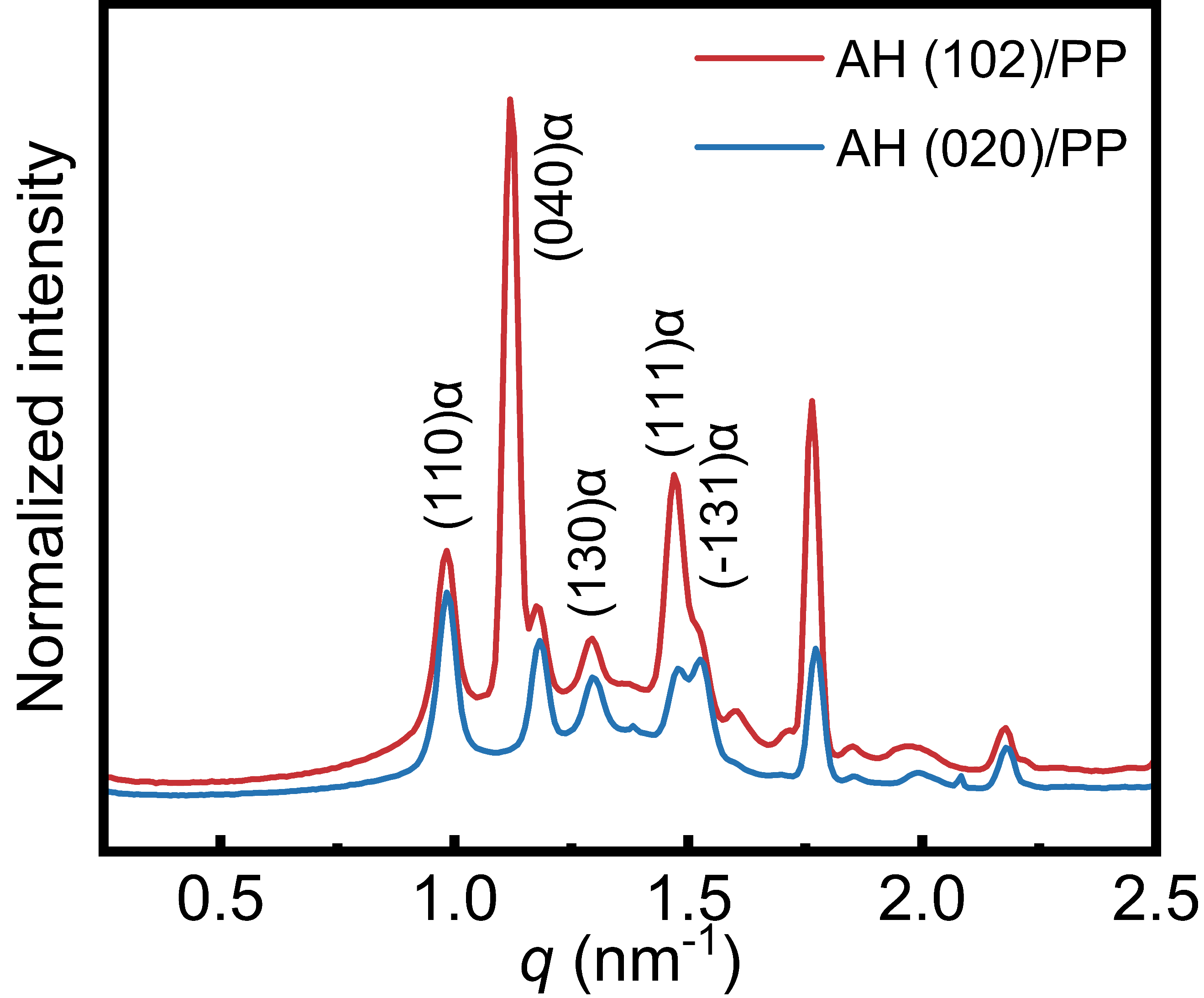


**Figure S26.** The 1D-WAXS curves integrated from 2D-WAXS patterns of the AH/PP composites.


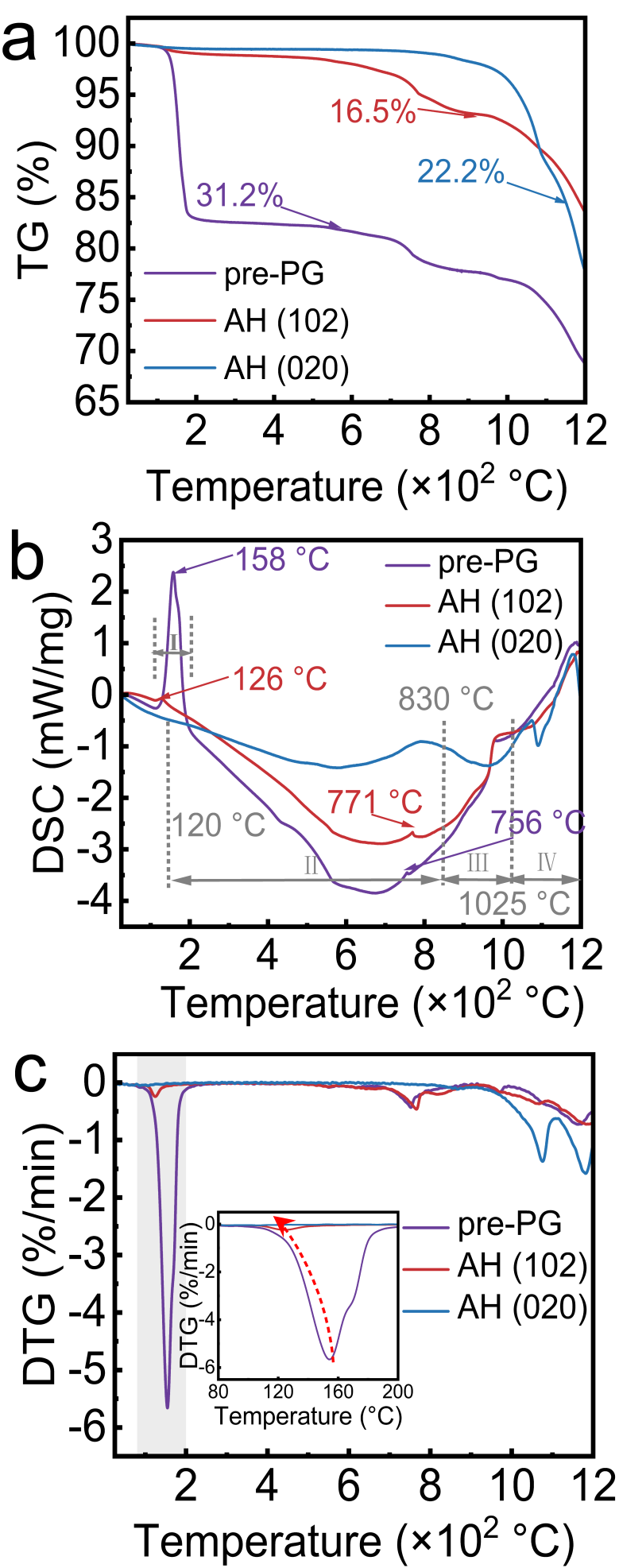


**Figure S27.** TG-DSC curves of pre-PG and AH particles. From 120-830 °C, the crystalline structure that transitioned from AH III to AH II in all samples (stage II in the DSC curves), eventually transform to AH I (stage III in the DSC curves),^[9]^ which may be accompanied by dehydration of portlandite and decarbonization of calcite. According to the TG curve, the quantity of the AH began to diminish at 1025 °C, which indicated the decomposition of AH (stage IV in the DSC curve), corresponding to previous studies,^[27]^ indicating that AH has good heat resistance and thermal stability.


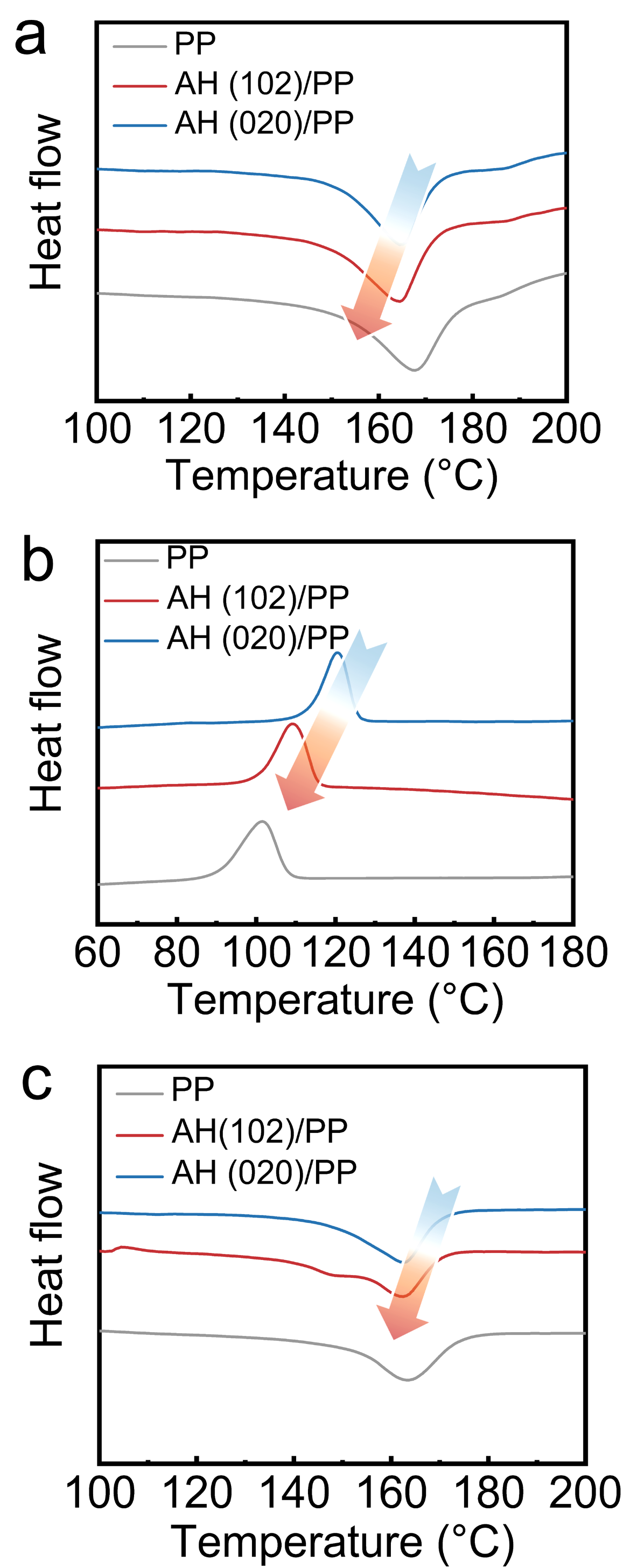


**Figure S28.** The DSC curve of the neat PP and AH/PP composites: (a) The first heating stage, (b) The first cooling stage and (c) The second heating stage.

The effect of the AH with different facet ratios on the crystallization behavior of PP was investigated by DSC test, as shown in **Figure S27**. The crystallinity $X_{c}$ of each sample was calculated using $X_{c}=\Delta H_{c}/\Delta H_{m}$, where $\Delta H_{c}$ is the experimental enthalpy and $\Delta H_{m}$ is the theoretical melting enthalpy of 100% crystallized PP, which is 209 J/g.^[28]^ The $\Delta H_{c}$, $X_{c}$, $T_{m}$ (two heating stages) and $T_{c}$ (one cooling stage) of all IPCs are shown in **Table S5**. All the PG and AH-containing composites present a significant increase in crystallinity compared to pure PP. In general, higher crystallinity results in better mechanical strength, which is also consistent with the results of subsequent mechanical property tests. The two fillers, pre-PG and AH, caused a slight decrease in the $T_{m}$ of the composites in comparison to PP. The pre-PG/PP composites showed a larger decrease of about 5.17 °C, however, the AH/PP composites showed very little change of about 3.17 °C. The addition of the pre-PG and AH to PP increased the $T_{c}$ of the composites by 17.33 °C and 19.0 °C, respectively. The increase in $T_{c}$ can be attributed to the pre-PG and AH due to the shear caused by different thermal expansion coefficients of filler and matrix during cooling.^[29]^ The pre-PG and AH act as foreign particles in the PP matrix which provide numerous nuclei during the cooling process thus increasing the $T_{c}$. At the same time, the changes in the crystallization behavior of the composites also validates our point above about the charge transport-induced molecular orientation and diffusion rate which in turn affects the crystallization behavior.


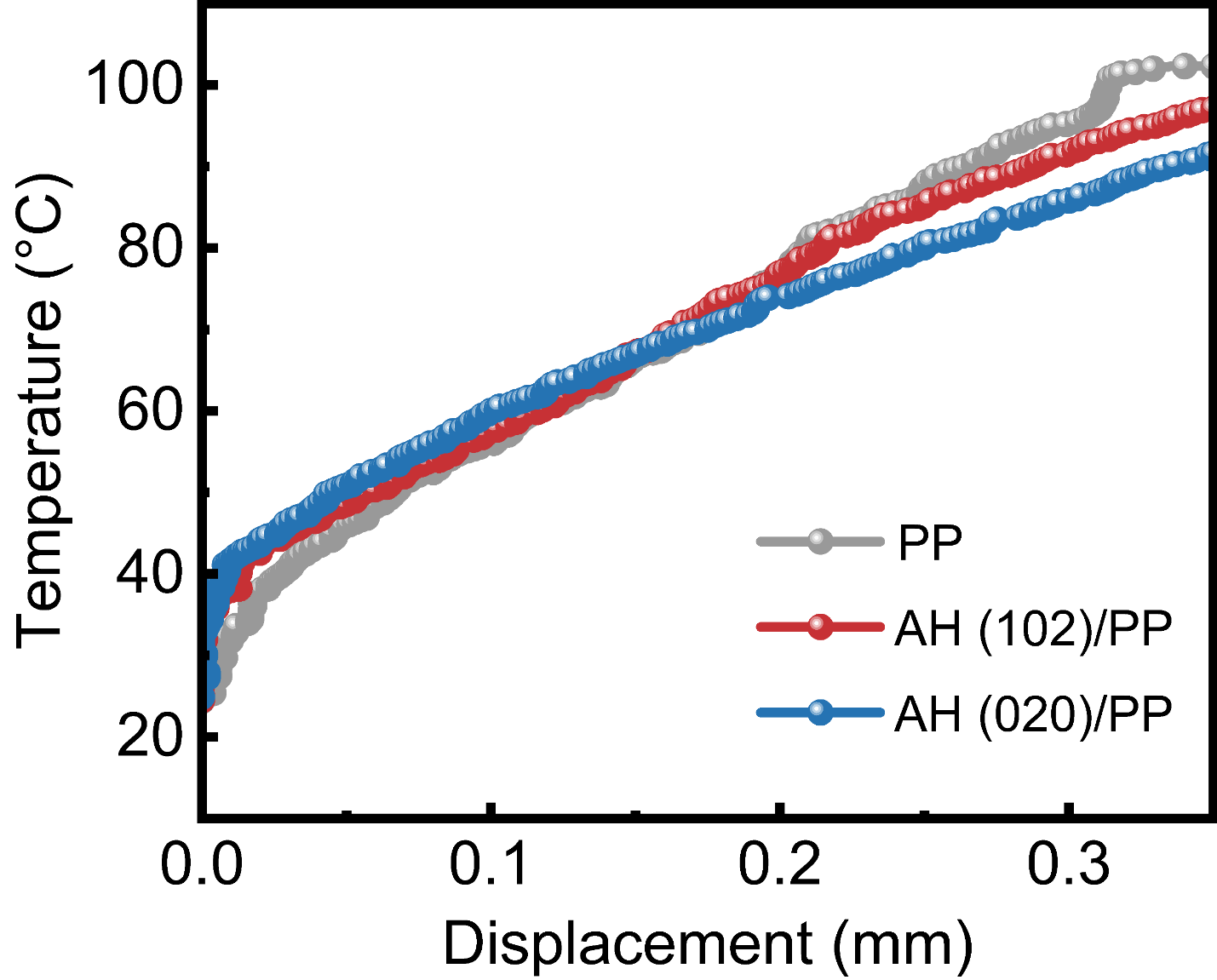


**Figure S29.** The HDT curve of the neat PP and AH/PP composites. Typically, the composites face challenges in terms of toughness and heat deflection temperature due to the presence of hard fillers. This significantly inhibits the utilization of composites in specific applications for industries such as automotive and electronics.^[30]^ We conducted heat deflection temperature tests on all AH/PP composites. Even with the addition of 10 wt.% AH, the HDT of the composites are similar to that of pure PP, with good dimensional stability and heat resistance.


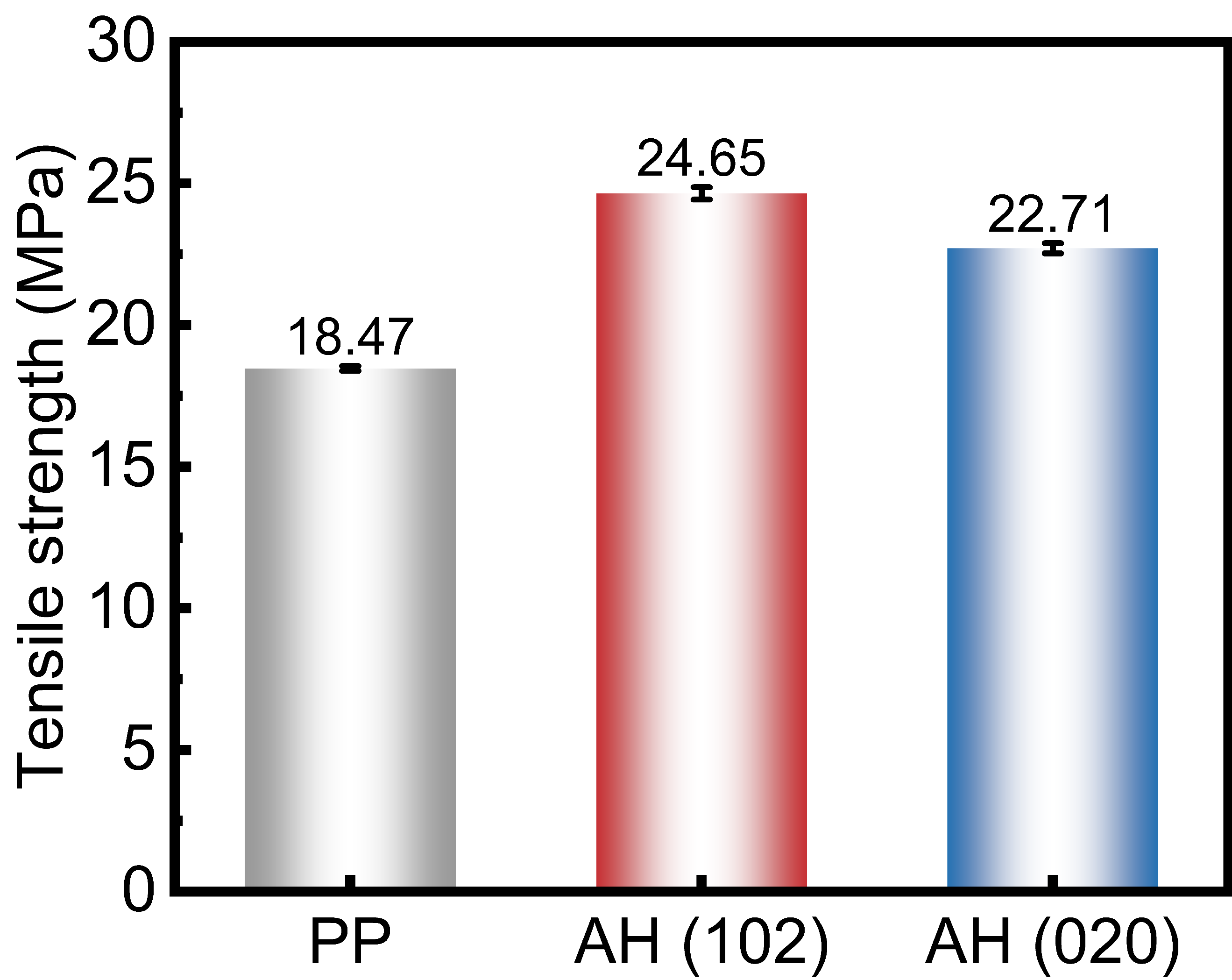


**Figure S30.** The tensile strength of the pure PP and AH/PP composites.


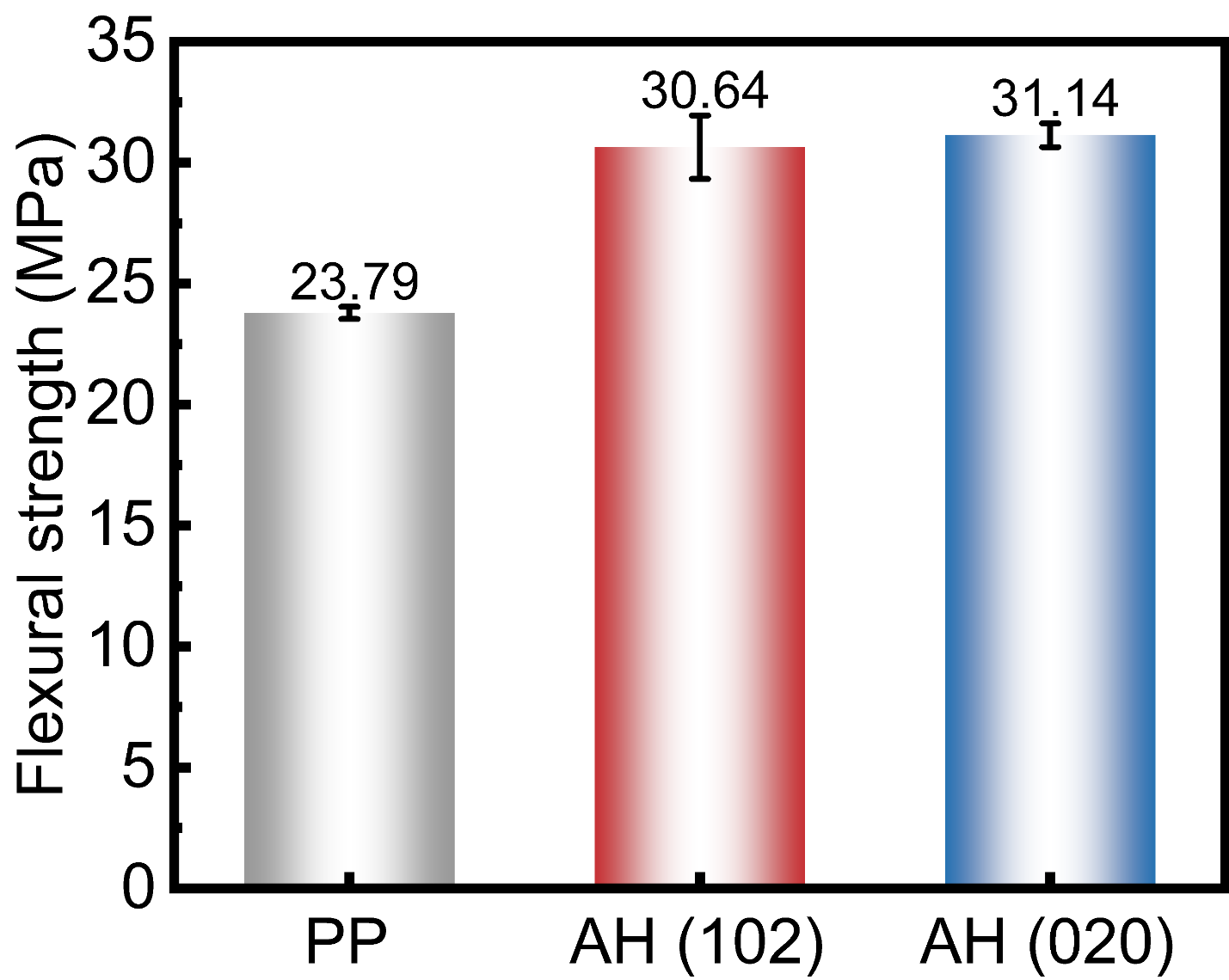


**Figure S31.** The flexural strength of the pure PP and AH/PP composites.


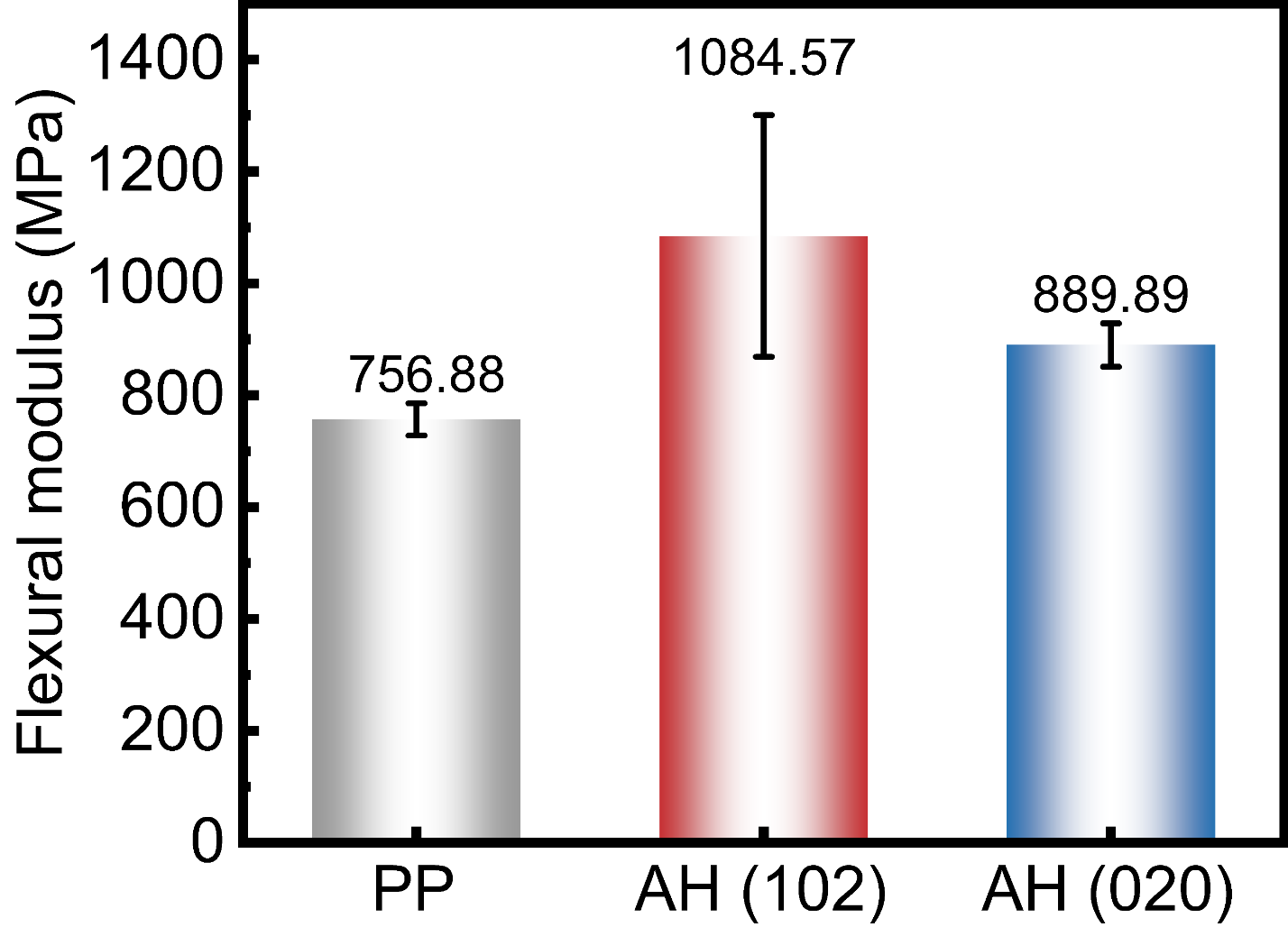


**Figure S32.** The flexural modulus of the pure PP and AH/PP composites.


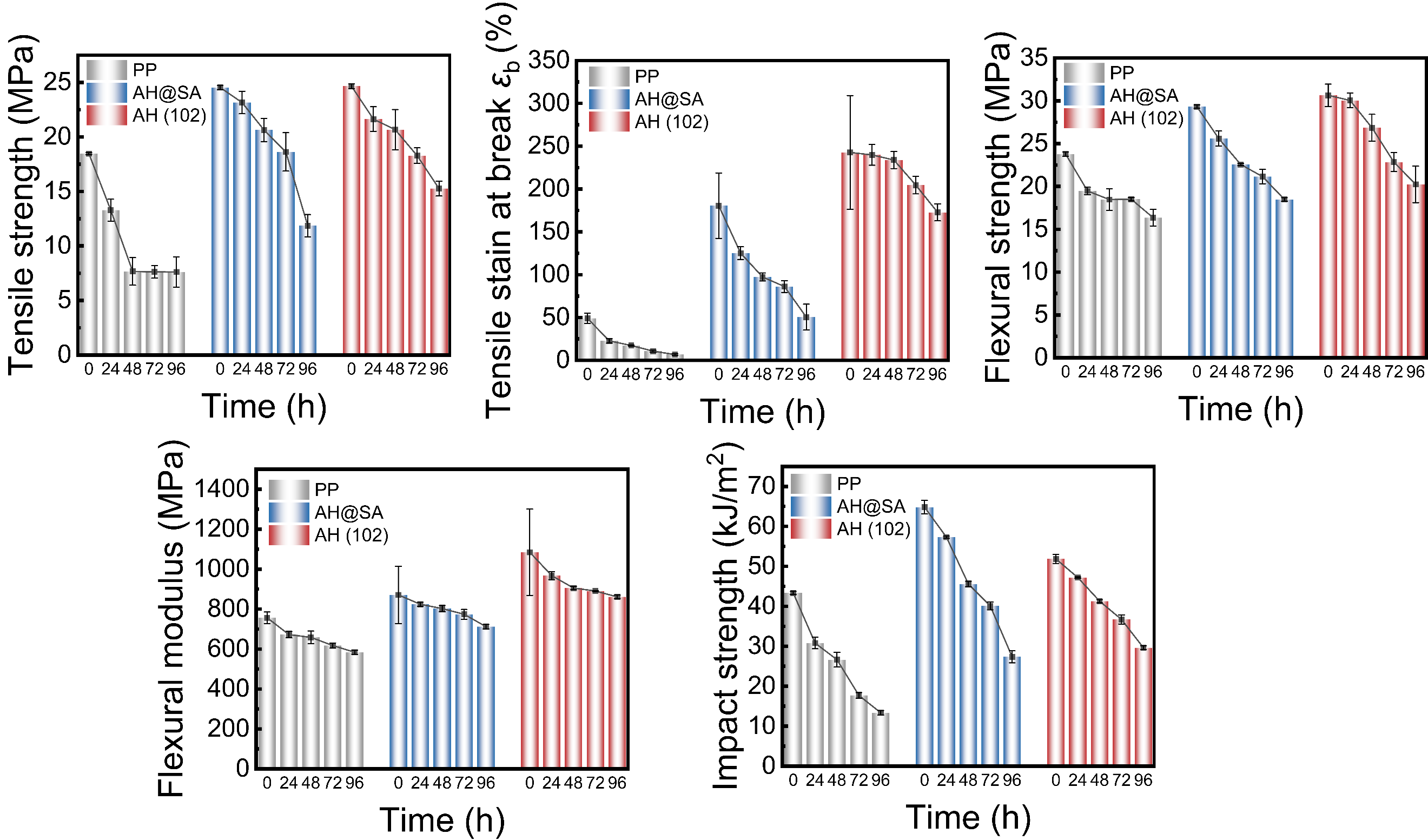


**Figure S33.** The mechanical properties of the pure PP, AH@SA and AH (102)/PP composites after UV aging treatment.


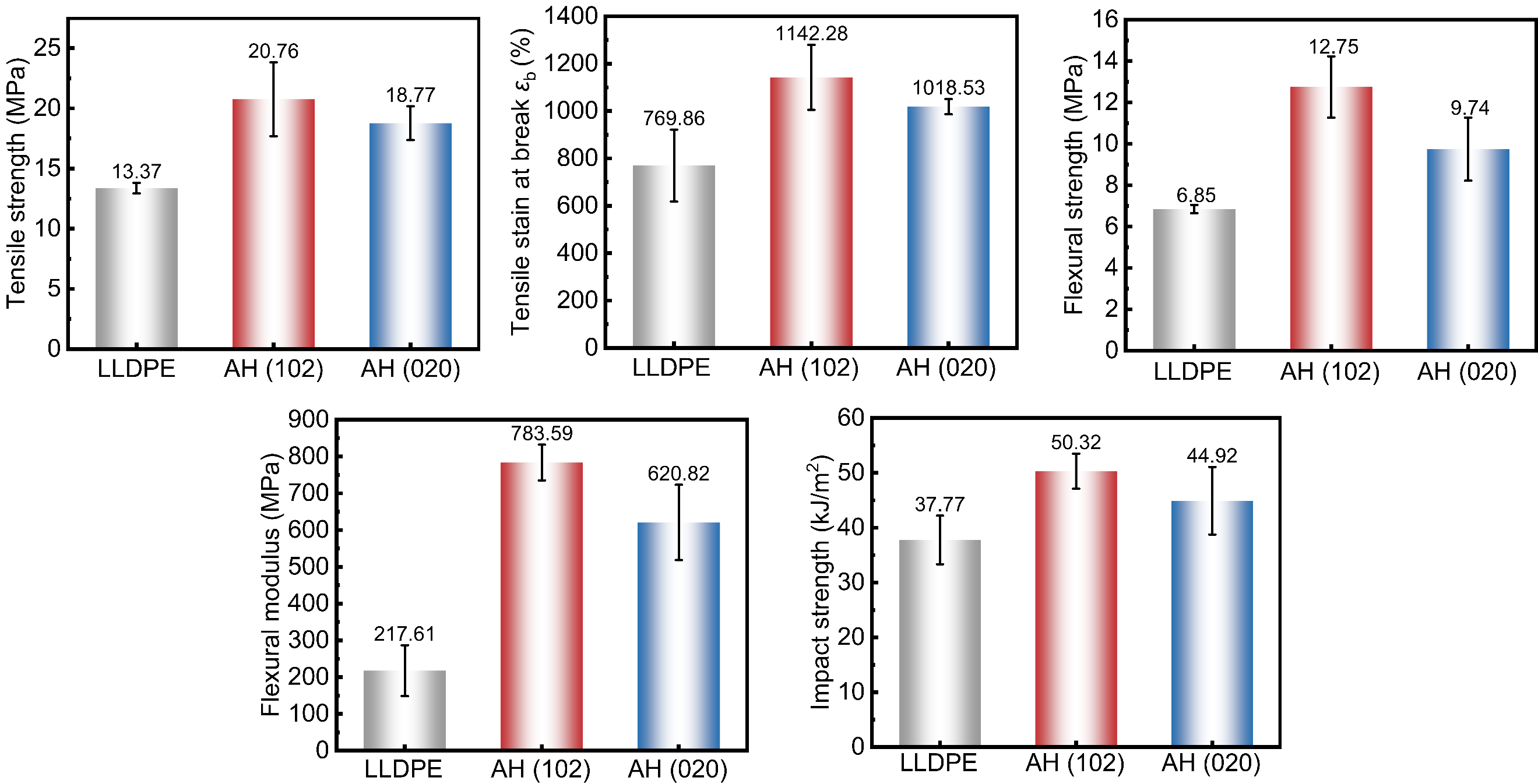


**Figure S34.** The mechanical properties of the pure LLDPE and AH/LLDPE composites.


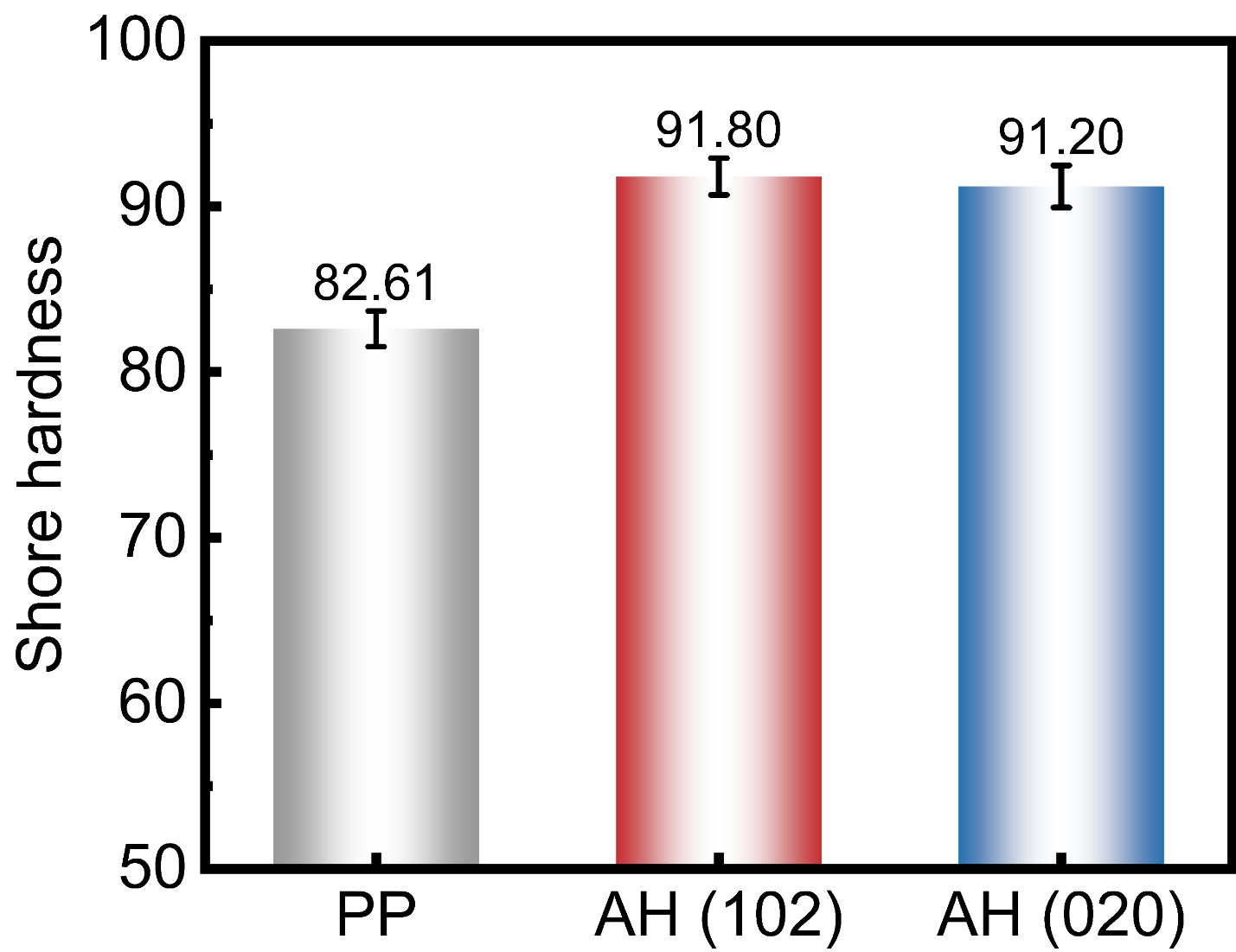


**Figure S35.** The shore hardness of the pure PP and AH/PP composites.


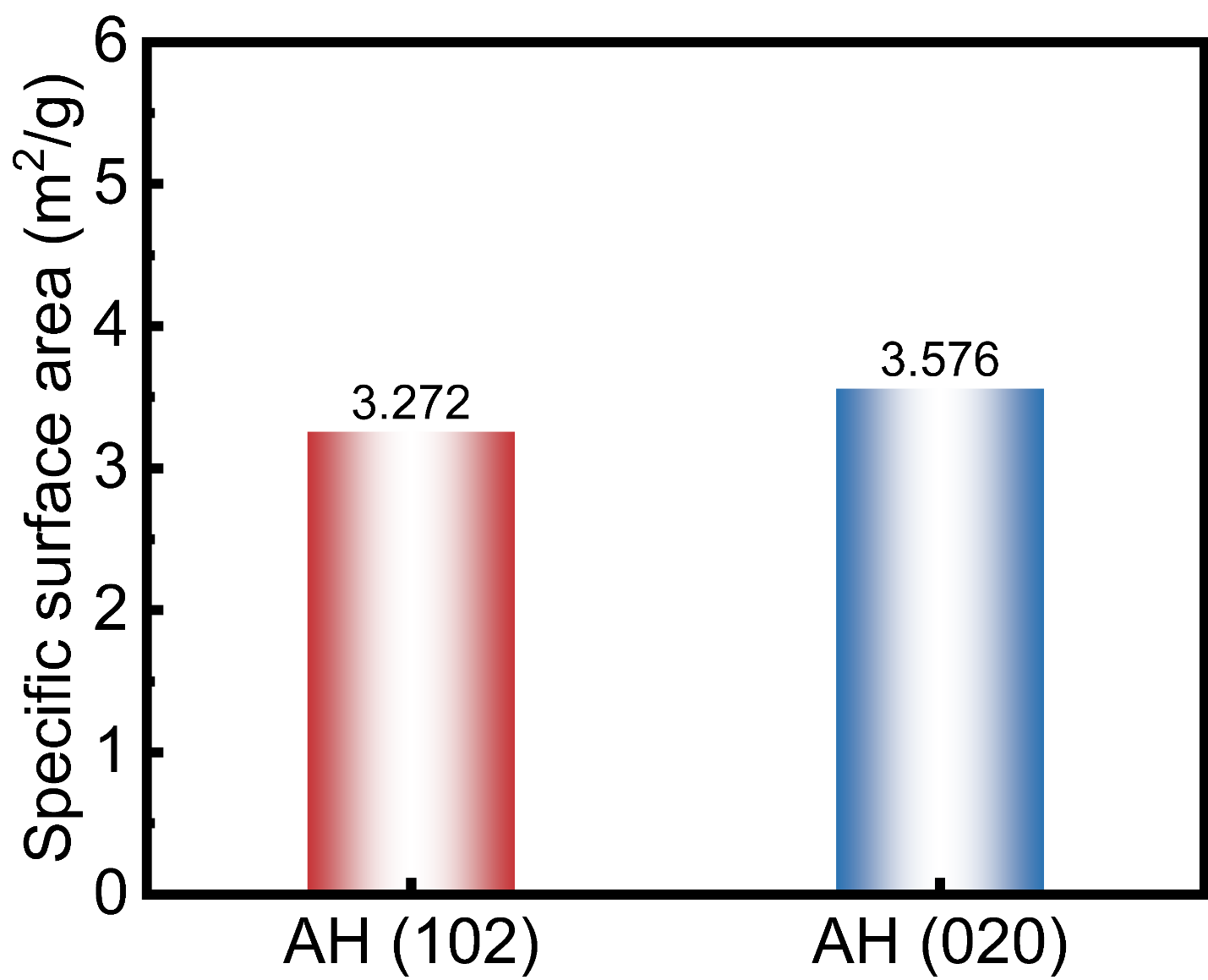


**Figure S36.** The specific surface area of AH particles.


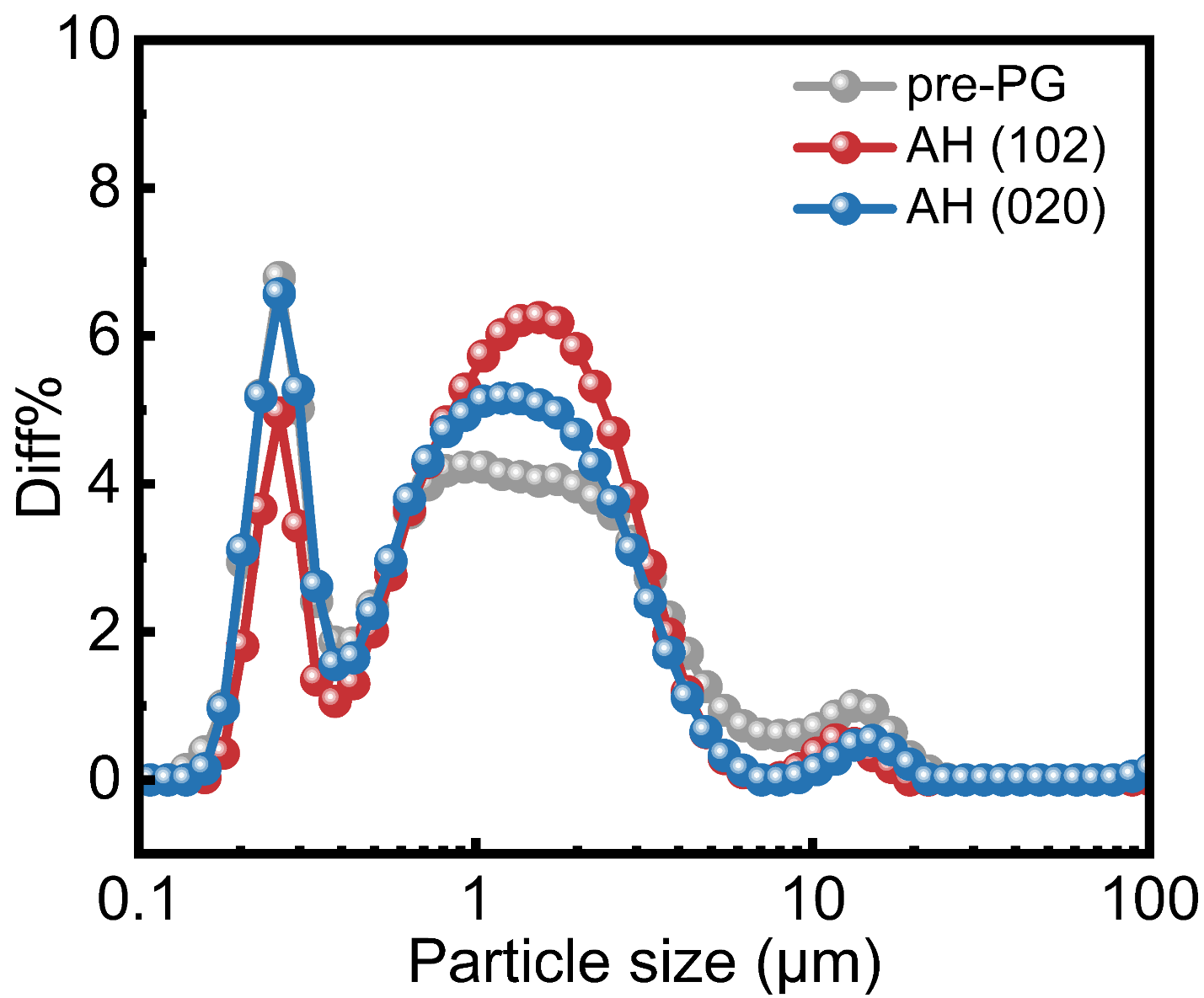


**Figure S37.** The particle size distribution of pre-PG and all AH particles.


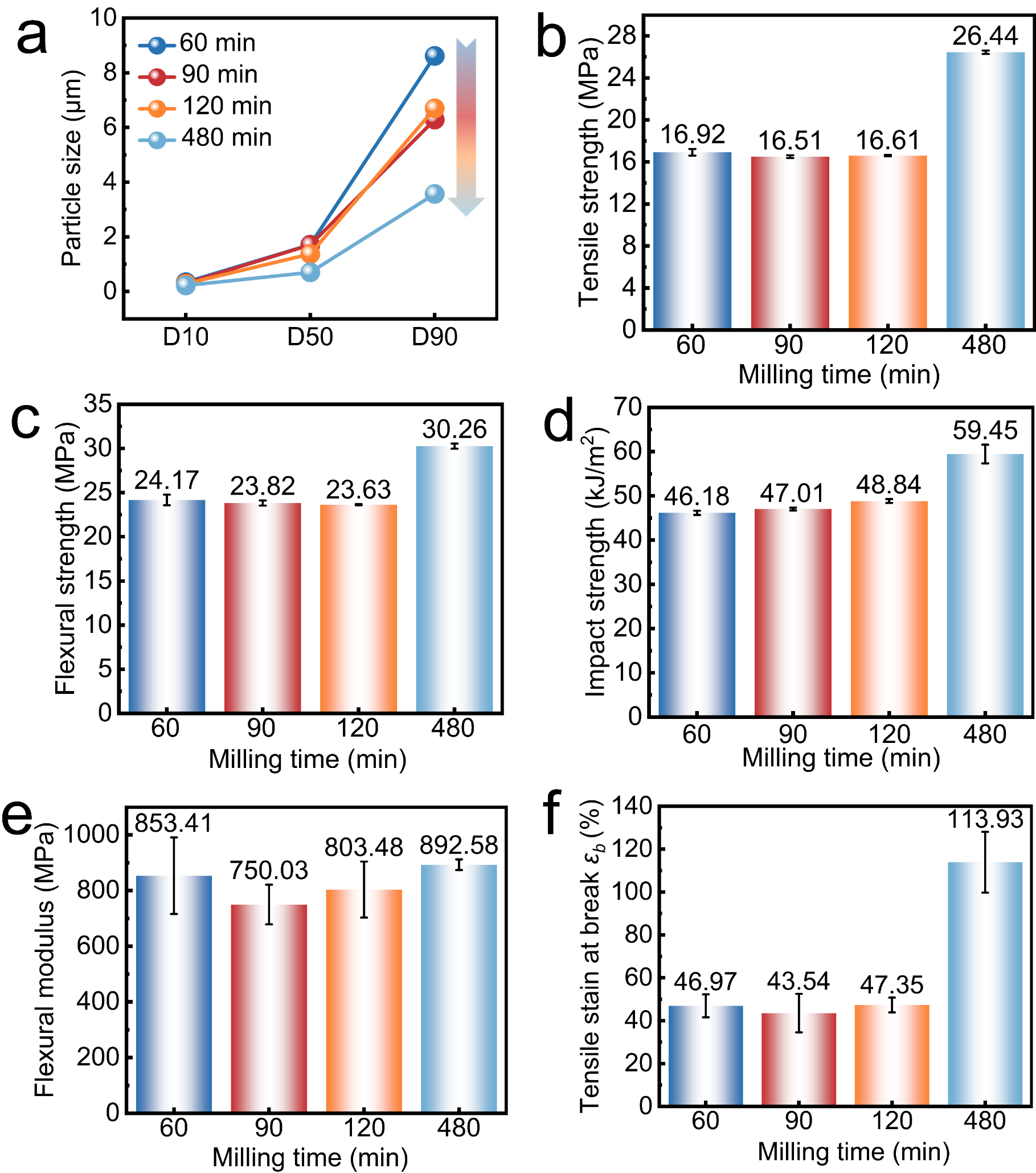


**Figure S38.** (a) The variation of particle size of the AH treated with different ball milling times, (b), (c), (d), (e), and (f) The tensile strength, flexural strength, impact strength, flexural modulus, and tensile strain at break ε_b_ of the IPCs corresponding to the PG treated with different ball milling times, respectively.


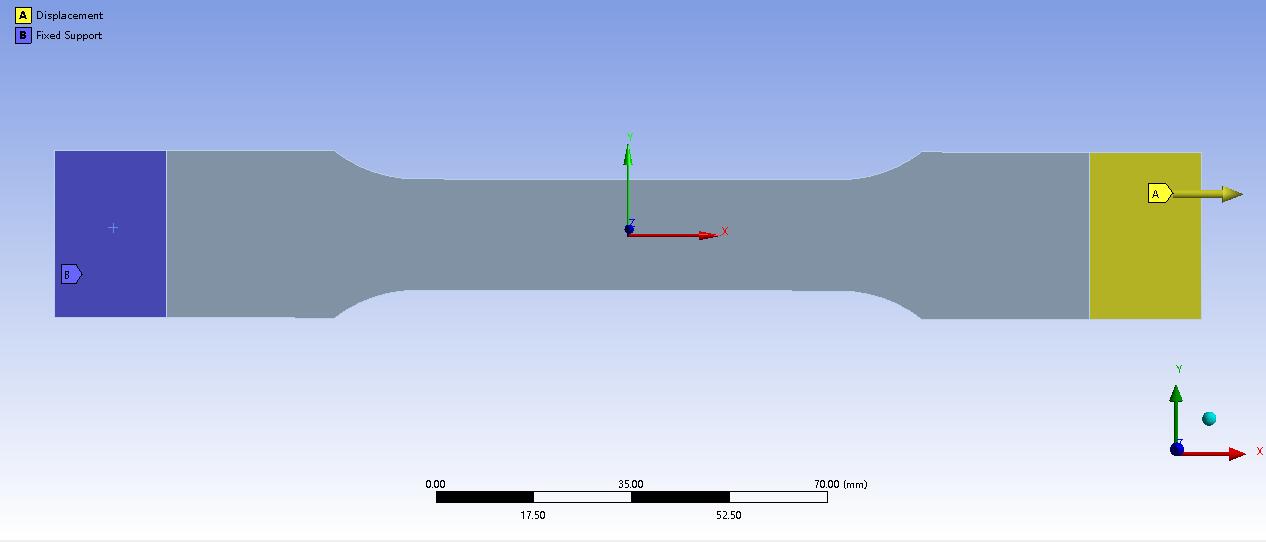


**Figure S39.** Diagram of the model with loaded boundary conditions.


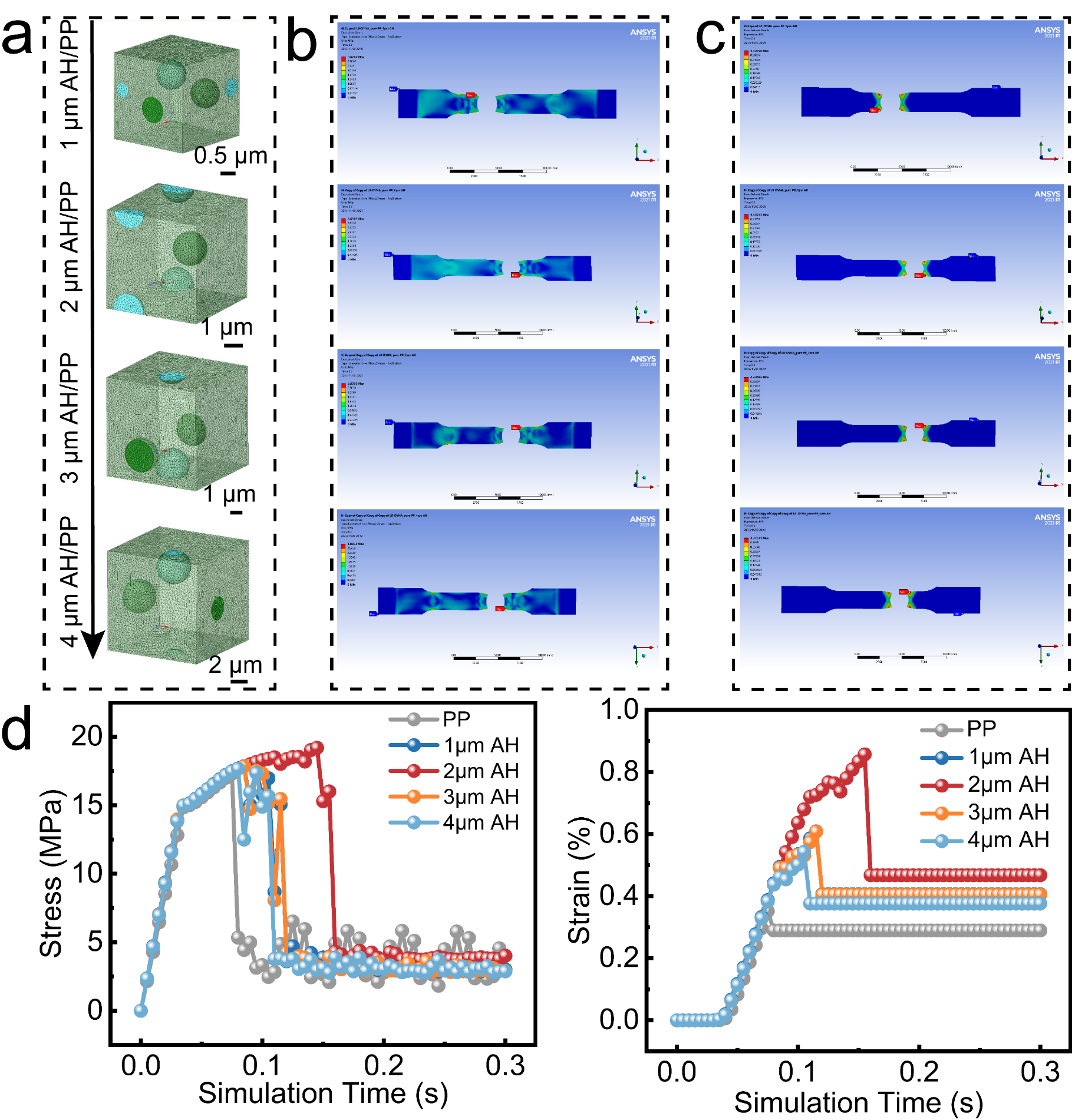


**Figure S40.** (a) The RVE model of the PP composites containing AH with different particle sizes, (b) The stress diagram of the composite materials in the state of tensile fracture, (c) The strain diagram of the composite materials in the state of tensile fracture and (d) The stress and strain curves of the composite materials as a function of simulation time.


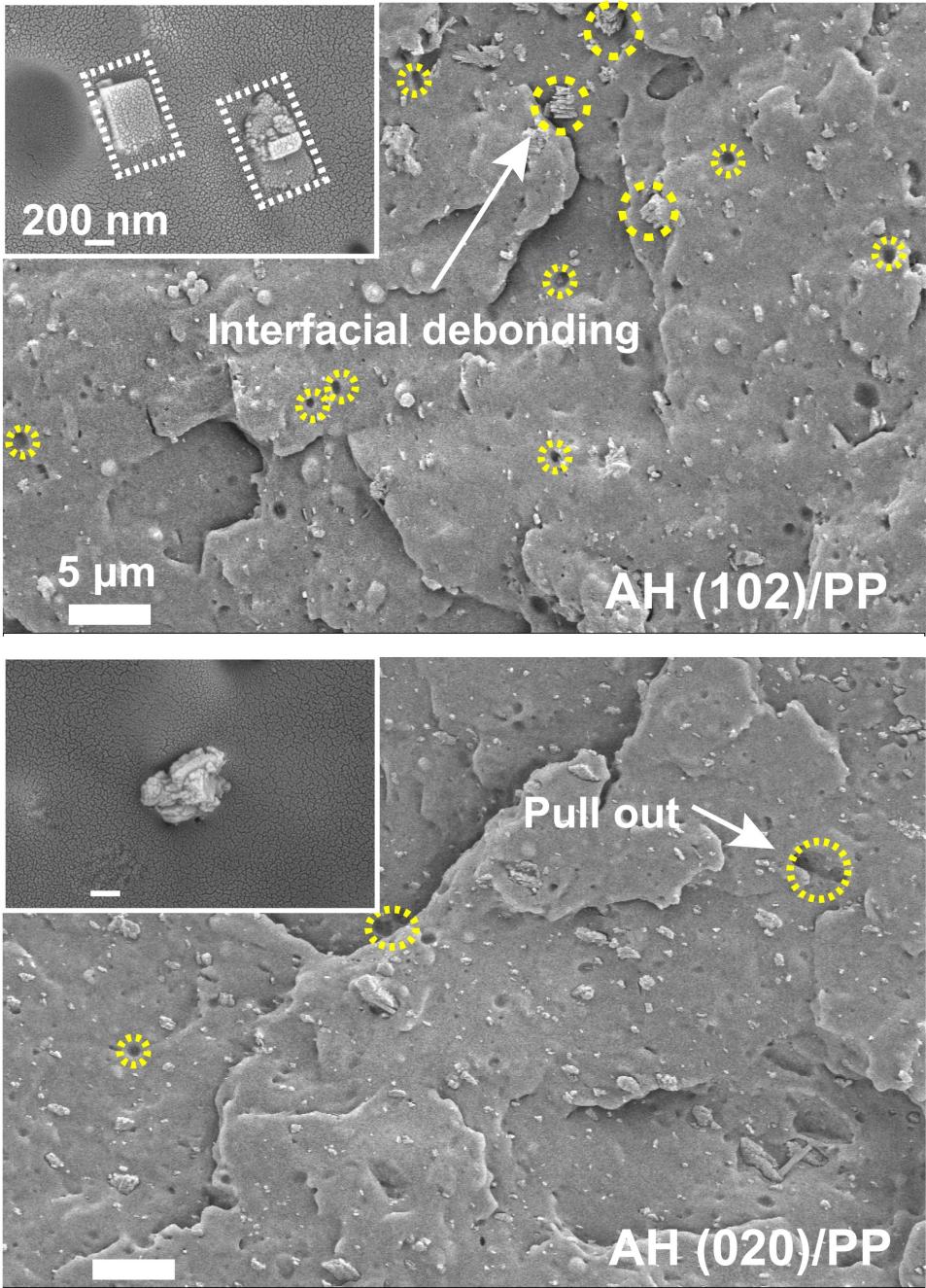


**Figure S41.** The fracture surfaces of the AH/PP composites at 2000X magnification.


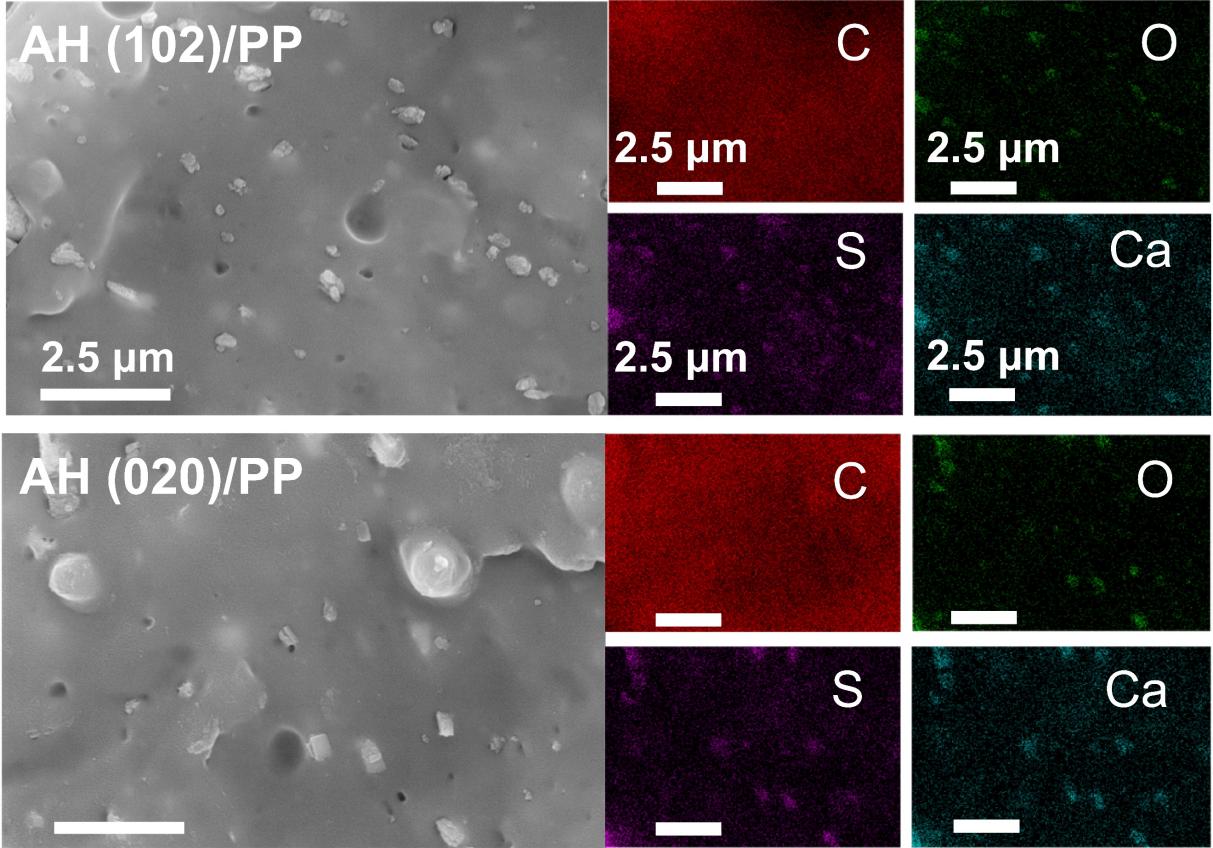


**Figure S42.** The SEM-EDS images of the AH/PP composites.


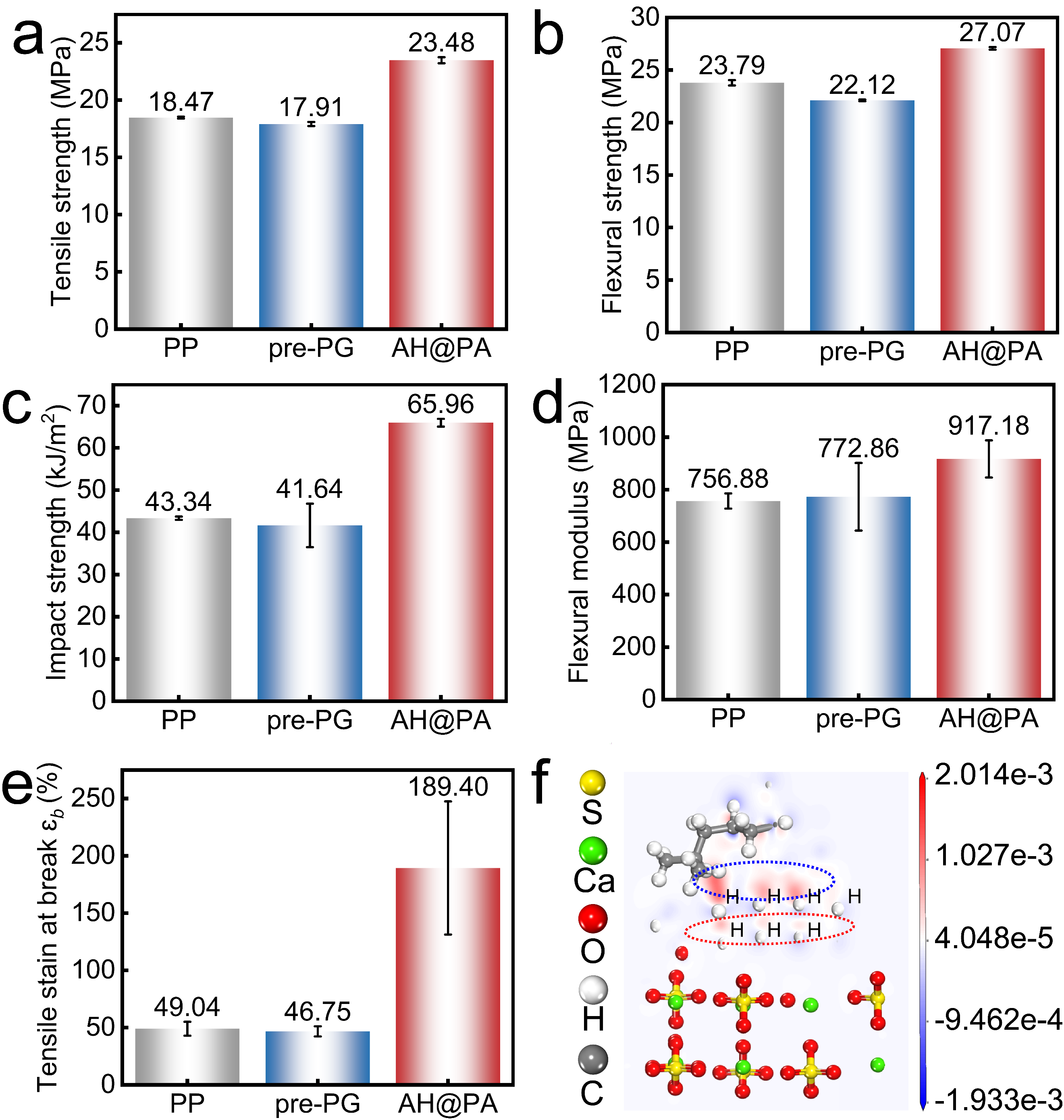


**Figure S43.** (a-e) The mechanical properties of the AH@PA/PP composites and (f) The EDD of the AH(020)@DA/PP and AH(102)@DA/PP composites of the slices in side views (blue and red colors represent charge depletion and accumulation, respectively).


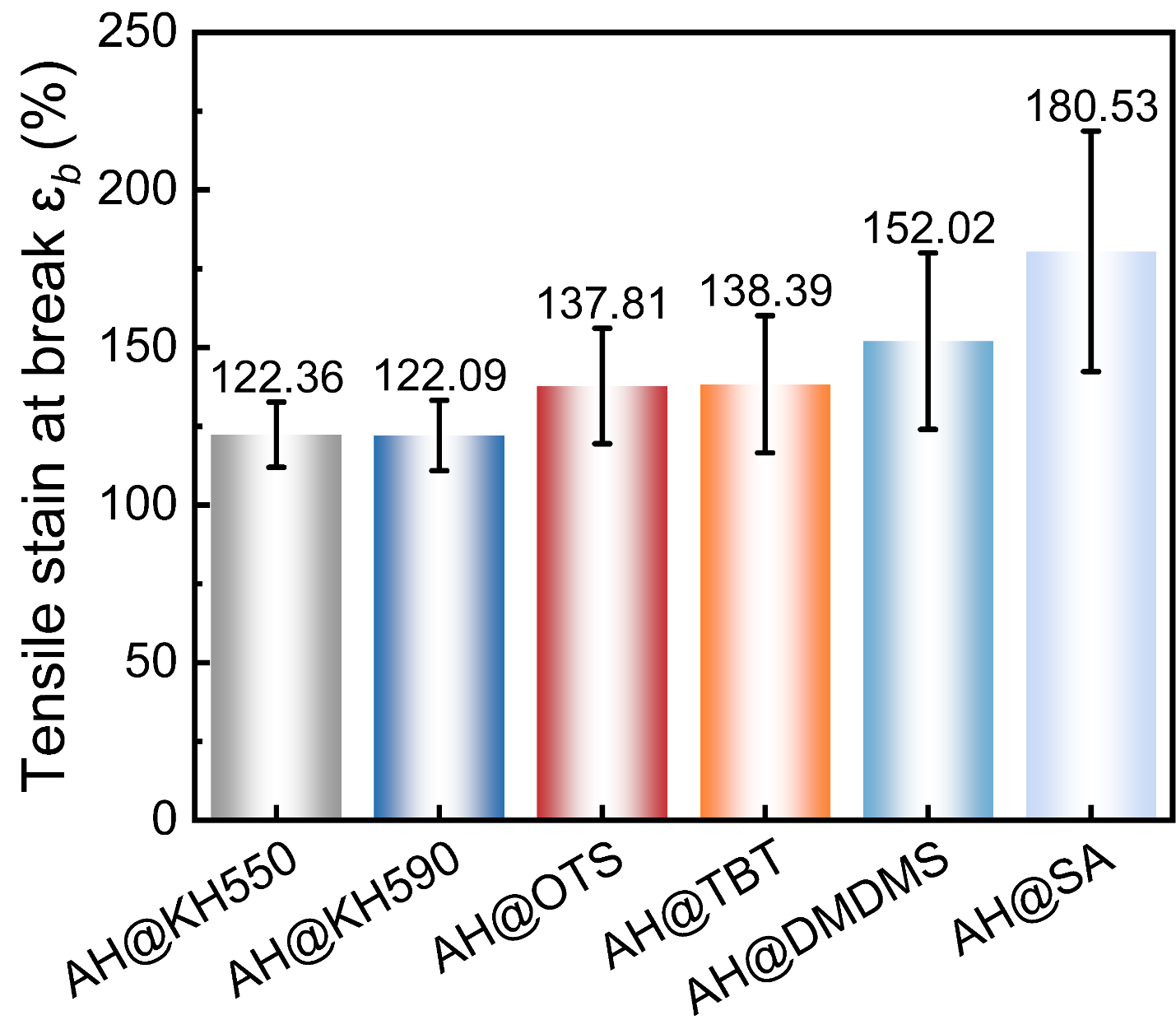


**Figure S44.** The tensile strains at break ε_b_ of AH/PP composites modified with coupling agent KH550, coupling agent KH590, trimethoxysilane (OTS), tetra-n-butyl titanate (TBT), dimethyldimethoxysilane (DMDMS) and stearic acid (SA), respectively. The above experiments show that even with organic modification, the tensile strains at break of the samples are lower than those of the unmodified AH/PP composites in this work.

1. **Supplementary Tables**

**Table S1.** The composites research works listed in **Figure 5g**.

| Reference | |
| --- | --- |
| Ref.S1^[31]^ | Ref.S19^[32]^ |
| Ref.S2^[33]^ | Ref.S20^[34]^ |
| Ref.S3^[35]^ | Ref.S21^[36]^ |
| Ref.S4^[37]^ | Ref.S22^[38]^ |
| Ref.S5^[39]^ | Ref.S23^[40]^ |
| Ref.S6^[41]^ | Ref.S24^[42]^ |
| Ref.S7^[43]^ | Ref.S25^[44]^ |
| Ref.S8^[45]^ | Ref.S26^[46]^ |
| Ref.S9^[47]^ | Ref.S27^[48]^ |
| Ref.S10^[49]^ | Ref.S28^[50]^ |
| Ref.S11^[51]^ | Ref.S29^[52]^ |
| Ref.S12^[53]^ | Ref.S30^[54]^ |
| Ref.S13^[55]^ | Ref.S31^[56]^ |
| Ref.S14^[57]^ | Ref.S32^[50]^ |
| Ref.S15^[58]^ | Ref.S33^[59]^ |
| Ref.S16^[60]^ | Ref.S34^[61]^ |
| Ref.S17^[62]^ | Ref.S35^[63]^ |
| Ref.S18^[64]^ |  |

**Table S2.** Chemical composition (wt.%) of raw materials.

| Material | SO_3_ | CaO | SiO_2_ | P_2_O_5_ | Fe_2_O_3_ | Al_2_O_3_ | K_2_O | Na_2_O | F |
| --- | --- | --- | --- | --- | --- | --- | --- | --- | --- |
| untreated-PG | 49.71 | 40.50 | 5.66 | 0.93 | 0.75 | 0.62 | 0.58 | 0.42 | 0.29 |
| Pre-PG | 47.10 | 41.05 | 7.27 | 0.85 | 1.05 | 0.69 | 0.53 | 0.18 | 0.42 |
| AH (102) | 48.26 | 40.17 | 7.46 | 0.78 | 0.98 | 0.74 | 0.47 | 0.38 | / |
| AH (020) | 46.47 | 41.79 | 7.47 | 0.85 | 1.13 | 0.73 | 0.54 | 0.17 | / |

**Table S3.** The E_int_ (kcal/mol) between the facets of PG and AH with PP molecule in the simulation time of 500 ps.

| Filler | Facet | Mean | Standard Deviation | Median |
| --- | --- | --- | --- | --- |
| PG | (020) | -48.51 | 3.99 | -48.93 |
|  | (-121) | -41.53 | 3.68 | -42.11 |
|  | (031) | -41.73 | 7.88 | -43.60 |
|  | (-141) | -44.36 | 5.90 | -45.59 |
|  | (002) | -30.93 | 5.07 | -31.08 |
| AH | (020) | -67.94 | 3.68 | -68.47 |
|  | (102) | -61.70 | 4.34 | -62.16 |
|  | (220) | -54.15 | 4.31 | -54.69 |
|  | (022) | -47.67 | 5.35 | -48.46 |
|  | (122) | -69.62 | 4.87 | -69.74 |

**Table S4.** The E_int_ (kcal/mol) between the facets of PG and AH with H_2_O molecule in the simulation time of 500 ps.

| Filler | Facet | Mean | Standard Deviation | Median |
| --- | --- | --- | --- | --- |
| PG | (020) | -40.41 | 3.39 | -40.38 |
|  | (-121) | -33.08 | 2.34 | -33.08 |
|  | (031) | -31.82 | 5.41 | -31.66 |
|  | (-141) | -29.79 | 3.13 | -29.89 |
|  | (002) | -25.43 | 4.21 | -25.58 |
| AH | (020) | -28.19 | 2.54 | -28.33 |
|  | (102) | -50.39 | 2.59 | -50.50 |
|  | (220) | -26.49 | 2.54 | -26.60 |
|  | (022) | -38.65 | 4.32 | -39.12 |
|  | (122) | -40.38 | 7.13 | -37.71 |

**Table S5.** The $\Delta H_{c}$, $X_{c}$, $T_{m}$ (two heating stages) and $T_{c}$ (one cooling stage) of all IPCs.

| Samples | $\Delta H_{c}$ (J/g) | $X_{c}$ (%) | $T_{m}$ (°C, heating Stage 1) | $T_{c}$ (°C, cooling Stage) | $T_{m}$ (°C, heating Stage 2) |
| --- | --- | --- | --- | --- | --- |
| PP | 49.93 | 23.89 | 167.67 | 101.50 | 163.50 |
| pre-PG/PP | 62.44 | 29.88 | 162.50 | 118.83 | 158.26 |
| AH (102)/PP | 64.49 | 30.86 | 164.50 | 109.17 | 162.50 |
| AH (020)/PP | 71.18 | 34.06 | 164.50 | 120.50 | 162.50 |

**Table S6.** The particle size of the pre-PG, AH particles and the difference Δ in particle size between adjacent these.

| Sample | D10 (μm) | Δ (μm) | D50 (μm) | Δ (μm) | D90 (μm) | Δ (μm) |
| --- | --- | --- | --- | --- | --- | --- |
| pre-PG | 0.231 | / | 0.960 | / | 4.002 | / |
| AH (102) | 0.255 | 0.024 | 1.136 | 0.176 | 2.817 | 1.185 |
| AH (020) | 0.232 | 0.023 | 0.927 | 0.209 | 2.744 | 0.073 |

**Table S7.** The particle size of the samples after ball milling and the difference Δ in particle size between adjacent ball milling times.

| Milling time (min) | D10 (μm) | Δ (μm) | D50 (μm) | Δ (μm) | D90 (μm) | Δ (μm) |
| --- | --- | --- | --- | --- | --- | --- |
| 60 | 0.3412 | / | 1.7110 | / | 8.6154 | / |
| 90 | 0.2978 | 0.0434 | 1.7044 | 0.0066 | 6.2854 | 2.33 |
| 120 | 0.2852 | 0.0126 | 1.3732 | 0.3312 | 6.6970 | 0.4116 |
| 480 | 0.2207 | 0.0645 | 0.6982 | 0.6750 | 3.5660 | 3.1310 |

**Table S8.** The parameters for AH/PP composite.

| Parameter | 1 μm AH/PP | 2 μm AH/PP | 3 μm AH/PP | 4 μm AH/PP |
| --- | --- | --- | --- | --- |
| Matrix | PP | | | |
| Particle | AH | | | |
| Particle diameter (μm) | 1 | 2 | 3 | 4 |
| Mesh maximum size (μm) | 0.1 | 0.2 | 0.3 | 0.4 |

1. **Supplementary References**

[1] a) D. J. Gao, D. A. Zhang, Y. Z. Peng, H. L. Diao, W. Q. Wang, *Constr Build Mater.* **2022**, 316, 126037; b) T. Chakraborty, A. Hens, S. Kulashrestha, N. C. Murmu, P. Banerjee, *Physica E.* **2015**, 69, 371.

[2] a) Q. Xu, J. Zhang, X. Li, D. M. van Duin, Y. Z. Hu, A. C. T. van Duin, T. B. Ma, *Acs Appl Mater Inter.* **2022**, 14, 6239; b) W. W. Chu, M. A. Webb, C. T. Deng, Y. J. Colón, Y. Kambe, S. Krishnan, P. F. Nealey, J. J. de Pablo, *Macromolecules* **2020**, 53, 2783.

[3] a) S. H. Kwon, H. S. Kang, B. J. Kim, H. I. Lee, J. M. Lee, J. Kim, S. G. Lee, *Sci. Rep.* **2023**, 13, 138; b) M. Rezayani, F. Sharif, H. Makki, *J Mater Chem A.* **2022**, 10, 18295.

[4] M. Farahani, Y. Jahani, A. Kakanejadifard, M. Ohshima, H. A. Shnawa, *Mater Today Commun.* **2022**, 31, 103529.

[5] X. W. Liu, X. H. Shen, H. Li, P. Li, L. B. Luo, H. M. Fan, X. M. Feng, W. H. Chen, X. P. Ai, H. X. Yang, Y. L. Cao, *Adv Energy Mater.* **2021**, 11, 2003905.

[6] X. L. Zhang, F. Niu, D. H. Liu, S. M. Yang, Y. M. Yang, Z. F. Tong, *Rsc Adv.* **2019**, 9, 32085.

[7] D. D. Ye, X. J. Lei, T. Li, Q. Y. Cheng, C. Y. Chang, L. B. Hu, L. N. Zhang, *Acs Nano.* **2019**, 13, 4843.

[8] a) H. Li, D. Ai, L. L. Ren, B. Yao, Z. B. Han, Z. H. Shen, J. J. Wang, L. Q. Chen, Q. Wang, *Adv. Mater.* **2019**, 31, 1900875; b) X. Y. Huang, B. Sun, Y. K. Zhu, S. T. Li, P. K. Jiang, *PrMS.* **2019**, 100, 187; c) S. H. Park, J. Hwang, G. S. Park, J. H. Ha, M. Zhang, D. Kim, D. J. Yun, S. Lee, S. H. Lee, *Nat Commun.* **2019**, 10, 2537; d) S. L. Liu, W. Y. Liu, D. L. Ba, Y. Z. Zhao, Y. H. Ye, Y. Y. Li, J. P. Liu, *Adv. Mater.* **2023**, 35, 2110423.

[9] A. M. Lopez-Buendia, B. Garcia-Banos, M. M. Urquiola, J. M. Catala-Civera, F. L. Penaranda-Foix, *PCCP.* **2020**, 22, 27713.

[10] S. Yoshimura, S. Yoshihara, T. Shirakashi, E. Sato, *Electrochimica Acta*. **1994**, 39, 589.

[11] M. Zhou, S. Guo, J. L. Li, X. B. Luo, Z. X. Liu, T. S. Zhang, X. X. Cao, M. Q. Long, B. G. Lu, A. Q. Pan, G. Z. Fang, J. Zhou, S. Q. Liang, *Adv. Mater.* **2021**, 33, 2100187.

[12] a) D. Q. Zhang, G. S. Li, X. F. Yang, J. C. Yu, *ChCom.* **2009**, 4381; b) J. A. Zhu, S. H. Wang, Z. F. Bian, S. H. Xie, C. L. Cai, J. G. Wang, H. G. Yang, H. X. Li, *Crystengcomm.* **2010**, 12, 2219.

[13] a) Q. Li, X. Li, W. Li, L. Zhong, C. Zhang, Q. Y. Fang, G. Chen, *Chem. Eng. J.* **2019**, 369, 26; b) J. Li, C. Zhang, Q. Li, T. Gao, S. Yu, P. Tan, Q. Fang, G. Chen, *ChEnS.* **2022**, 251, 117438.

[14] Y. Tsuru, Y. Shinzato, Y. Saito, M. Shimazu, M. Shiono, M. Morinaga, *J. Ceram. Soc. Jpn.* **2010**, 118, 241.

[15] I. A. Tchmutin, A. T. Ponomarenko, E. P. Krinichnaya, G. I. Kozub, O. N. Efimov, *Carbon.* **2003**, 41, 1391.

[16] A. K. Yono, R. I. Keda, S. Akagi, W. N. Ishiyasu, *J Miner Petrol Sci.* **2022**, 117.

[17] V. H. S. Souza, A. A. R. Santos, A. L. G. Costa, F. L. Santos, R. R. Magalhaes, *Comput. Electron. Agric.* **2018**, 150, 476.

[18] R. M. H. Khorasany, A. MohammadPanah, S. G. Hutton, *J Vib Acoust.* **2012**, 134, 061004.

[19] a) J. J. Liu, S. X. Qu, Z. G. Suo, W. Yang, *Natl Sci Rev* **2021**, 8; a) B. W. Liu, H. B. Zhao, Y. Z. Wang, *Adv. Mater.* **2022**, 34, 2107905.

[20] a) S. Kamalakannan, M. Prakash, M. M. Al-Mogren, G. Chambaud, M. Hochlaf, *J Phys Chem C.* **2019**, 123, 15087; b) C. L. Wei, K. Jiang, T. M. Fang, X. M. Liu, *Green Chem Eng.* **2021**, 2, 402; c) W. F. Feng, C. X. Zhang, J. X. Zhong, L. M. Ding, W. Q. Wu, *Chem. Commun.* **2020**, 56, 5006.

[21] S. J. Brotton, R. I. Kaiser, *J Phys Chem Lett.* **2013**, 4, 669.

[22] N. Buzgar, A. Buzatu, I. V. Sanislav, *An. Stiint. U. Al. I-Mat.* **2009**, 55, 5.

[23] S. G. Ranjbar, G. Roudini, F. Barahuie, *J Energy Storage.* **2020**, 27, 101168.

[24] D. Yang, A. Velamakanni, G. Bozoklu, S. Park, M. Stoller, R. D. Piner, S. Stankovich, I. Jung, D. A. Field, C. A. Ventrice, R. S. Ruoff, *Carbon.* **2009**, 47, 145.

[25] T. Z. Hong, Z. H. Lv, X. Liu, W. Li, X. Y. Nai, Y. P. Dong, *Mater Design.* **2016**, 107, 117.

[26] X. X. Zhao, J. H. Hu, X. Lai, X. G. Yang, X. H. Chen, X. H. Chen, *PoCom.* **2016**, 37, 2360.

[27] S. Wu, W. L. Wang, C. Z. Ren, X. L. Yao, Y. G. Yao, Q. S. Zhang, Z. F. Li, *Constr Build Mater.* **2019**, 228, 116676.

[28] X. G. Li, Q. Yang, K. Zhang, L. S. Pan, Y. H. Feng, Y. F. Jia, N. Xu, *J Clean Prod.* **2022**, 375, 134097.

[29] B. C. Liu, Y. B. Li, T. Fei, S. Han, C. B. Xia, Z. H. Shan, J. L. Jiang, *Chem. Eng. J.* **2020**, 385, 123829.

[30] a) A. Sodergard, M. Stolt, *Prog. Polym. Sci.* **2002**, 27, 1123; b) Z. C. Zhang, X. R. Gao, Z. J. Hu, Z. Yan, J. Z. Xu, L. Xu, G. J. Zhong, Z. M. Li, *Ind. Eng. Chem. Res.* **2016**, 55, 10896.

[31] J. Kim, D. Cho, *Polymers-Basel.* **2020**, 12, 2578.

[32] H. Demir, D. Balköse, S. Ülkü, *Polym. Degrad. Stab.* **2006**, 91, 1079.

[33] J. E. Stamhuis, *Polym. Compos.* **1984**, 5, 202.

[34] M. J. Zaini, M. Y. A. Fuad, Z. Ismail, M. S. Mansor, J. Mustafah, *Polym. Int.* **1996**, 40, 51.

[35] L. Moyo, S. S. Ray, W. Sebati, V. Ojijo, *J. Appl. Polym. Sci.* **2017**, 134, 45024.

[36] V. N. Hristov, M. Krumova, S. Vasileva, G. H. Michler, *J. Appl. Polym. Sci.* **2004**, 92, 1286.

[37] K. Wang, J. S. Wu, L. Ye, H. M. Zeng, *Compos Part a-Appl S.* **2003**, 34, 1199.

[38] F. Asyadi, M. Jawaid, A. Hassan, M. U. Wahit, *Polym-Plast Technol.* **2013**, 52, 727.

[39] P. V. C. Rao, *Constr Build Mater.* **2020**, 235, 117505.

[40] I. Svab, V. Musil, A. Pustak, I. Smit, *Polym. Compos.* **2009**, 30, 1091.

[41] M. Barczewski, K. Salasinska, J. Szulc, *Polym. Test.* **2019**, 75, 1.

[42] K. Leontiadis, D. S. Achilias, I. Tsivintzelis, *Polymers-Basel* **2023**, 15.

[43] Q. H. Mu, C. Wei, S. Y. Feng, *Polym. Compos.* **2009**, 30, 131.

[44] M. Kodal, E. Demirhan, *Polym. Compos.* **2013**, 34, 1396.

[45] E. Ko, T. Kim, J. Ahn, S. Park, S. Pak, M. Kim, H. Kim, *Fibers and Polymers.* **2021**, 22, 2163.

[46] Y. Q. Zhao, D. A. Schiraldi, *Polymer.* **2005**, 46, 11640.

[47] S. Debnath, R. Ranade, S. L. Wunder, J. McCool, K. Boberick, G. Baran, *Dent. Mater.* **2004**, 20, 677.

[48] C. M. Tai, R. K. Y. Li, *J. Appl. Polym. Sci.* **2001**, 80, 2718.

[49] S. Borysiak, *J. Appl. Polym. Sci.* **2013**, 127, 1309.

[50] G. X. Qiu, F. Raue, G. W. Ehrenstein, *J. Appl. Polym. Sci.* **2002**, 83, 3029.

[51] F. Gapsari, A. Purnowidodo, P. H. Setyarini, S. Hidayatullah, Suteja, H. Izzuddin, R. Subagyo, S. M. Rangappa, S. Siengchin, *Polym. Compos.* **2022**, 43, 1147.

[52] E. Chiellini, P. Cinelli, F. Chiellini, S. H. Imam, *Macromol Biosci.* **2004**, 4, 218.

[53] A. K. Nurdina, M. Mariatti, P. Samayamutthirian, *J. Appl. Polym. Sci.* **2011**, 120, 857.

[54] C. Spagnol, E. H. Fragal, M. A. Witt, H. D. M. Follmann, R. Silva, A. F. Rubira, *Carbohydr. Polym.* **2018**, 191, 25.

[55] G. L. Wang, D. M. Zhang, G. P. Wan, B. Li, G. Q. Zhao, *Polymer.* **2019**, 181.

[56] O. Balkan, H. Demirer, *Polym. Compos.* **2010**, 31, 1285.

[57] S. Erdogan, U. Huner, *J Wuhan Univ Technol.* **2018**, 33, 1298.

[58] P. Zhu, J. Chen, C. F. Wu, *Polym. Compos.* **2009**, 30, 391.

[59] P. Eteläaho, S. Haveri, R. Järvelä, *Polym. Compos.* **2011**, 32, 464.

[60] H. Y. Liu, C. Y. Liu, S. G. Peng, B. L. Pan, C. Lu, *Carbohydr. Polym.* **2018**, 182, 52.

[61] M. Pei, X. Y. Shang, Y. H. Xu, D. H. Zhang, *Polym. Compos.* **2023**, 44, 7040.

[62] Z. T. Yao, T. Chen, H. Y. Li, M. S. Xia, Y. Ye, H. Zheng, *J. Hazard. Mater.* **2013**, 262, 212.

[63] F. Özmihçi, D. Balköse, S. Ülkü, *J. Appl. Polym. Sci.* **2001**, 82, 2913.

[64] S. J. Liu, Y. Li, L. Li, *Carbohydr. Polym.* **2017**, 160, 62.
